# Author Correction: Octyl itaconate enhances VSVΔ51 oncolytic virotherapy by multitarget inhibition of antiviral and inflammatory pathways

**DOI:** 10.1038/s41467-024-55044-w

**Published:** 2024-12-19

**Authors:** Naziia Kurmasheva, Aida Said, Boaz Wong, Priscilla Kinderman, Xiaoying Han, Anna H. F. Rahimic, Alena Kress, Madalina E. Carter-Timofte, Emilia Holm, Demi van der Horst, Christoph F. Kollmann, Zhenlong Liu, Chen Wang, Huy-Dung Hoang, Elina Kovalenko, Maria Chrysopoulou, Krishna Sundar Twayana, Rasmus N. Ottosen, Esben B. Svenningsen, Fabio Begnini, Anders E. Kiib, Florian E. H. Kromm, Hauke J. Weiss, Daniele Di Carlo, Michela Muscolini, Maureen Higgins, Mirte van der Heijden, Rozanne Arulanandam, Angelina Bardoul, Tong Tong, Attila Ozsvar, Wen-Hsien Hou, Vivien R. Schack, Christian K. Holm, Yunan Zheng, Melanie Ruzek, Joanna Kalucka, Laureano de la Vega, Walid A. M. Elgaher, Anders R. Korshoej, Rongtuan Lin, John Hiscott, Thomas B. Poulsen, Luke A. O’Neill, Dominic G. Roy, Markus M. Rinschen, Nadine van Montfoort, Jean-Simon Diallo, Henner F. Farin, Tommy Alain, David Olagnier

**Affiliations:** 1 Department of Biomedicine, Aarhus University, 8000 Aarhus C, Denmark; 2 Department of Biochemistry Microbiology and Immunology, University of Ottawa, Ottawa, ON K1H 8M5 Canada; 3 Children’s Hospital of Eastern Ontario Research Institute, Ottawa, ON K1H 8L1 Canada; 4 Ottawa Hospital Research Insitute, Ottawa, ON K1H 8L6 Canada; 5 Department of Gastroenterology and Hepatology, Leiden University Medical Center, Leiden, The Netherlands; 6 Lady Davis Institute, Jewish General Hospital and Department of Medicine, McGill University, Montreal, QC H3T 1E2 Canada; 7 Georg-Speyer-Haus, Institute for Tumor Biology and Experimental Therapy, Frankfurt am Main, Germany; 8 Frankfurt Cancer Institute, Goethe University, Frankfurt am Main, Germany; 9 Faculty of Biological Sciences, Goethe University, 60438 Frankfurt am Main, Germany; 10 Department of Chemistry, Aarhus University, 8000 Aarhus C, Denmark; 11 School of Biochemistry and Immunology, Trinity College Dublin, Trinity Biomedical Sciences Institute, Dublin 2, Ireland; 12 Pasteur Laboratories, Istituto Pasteur Italia - Fondazione Cenci Bolognetti, Rome, 00161 Italy; 13 Jacqui Wood Cancer Centre, Division of Cellular Medicine, School of Medicine, University of Dundee, Dundee, UK; 14 Cancer Axis, CHUM Research Centre, Montreal, Canada; 15 Department of Microbiology, Infectious Diseases and Immunology, Faculty of Medicine, University of Montreal, Montreal, Canada; 16 Institut du Cancer de Montréal, Montreal, QC Canada; 17 Department of Neurosurgery, Aarhus University Hospital, 8200 Aarhus N, Denmark; 18 Department of Clinical Medicine, Aarhus University, 8200 Aarhus N, Denmark; 19 DCCC Brain Tumor Center, Copenhagen University Hospital, Copenhagen, Denmark; 20 Small Molecule Therapeutics & Platform Technologies, AbbVie Inc., 1 North Waukegon Road, North Chicago, IL 60064 USA; 21 AbbVie, Bioresearch Center, 100 Research Drive, Worcester, MA 01608 USA; 22 Steno Diabetes Center Aarhus, Aarhus University Hospital, Aarhus, Denmark; 23 Department of Drug Design and Optimization, Helmholtz Institute for Pharmaceutical Research Saarland (HIPS), Helmholtz Centre for Infection Research (HZI), Saarland University, E8.1, 66123 Saarbrücken, Germany; 24 III. Department of Medicine and Hamburg Center for Kidney Health, Hamburg, Germany; 25 Aarhus Institute of Advanced Studies, Aarhus University, Aarhus, Denmark; 26 German Cancer Consortium (DKTK), Frankfurt/Mainz partner site and German Cancer Research Center (DKFZ), 69120 Heidelberg, Germany

**Keywords:** Cancer immunotherapy, RIG-I-like receptors, Viral host response

Correction to: *Nature Communications* 10.1038/s41467-024-48422-x, published online 15 May 2024

The original version of this Article omitted from the author list the 28th author Rozanne Arulanandam, who is from the ‘Ottawa Hospital Research Insitute, Ottawa, ON, K1H 8L6, Canada’. Consequently, the following was added to the Author Contributions: ‘R.A. performed the revision experiments on cell lines shown in Figures 1 and 2, in particular, the virus titration and GFP measurements of virus infection in CT26wt and 76-9 with 4OI.’

The original version of this Article omitted funding details to R.L. The following was added to the Acknowledgements: ‘This research was supported by grants to R.L. from the Canadian Institutes of Health Research (CIHR) (PJT-169663).’

These have been corrected in both the PDF and HTML versions of the Article.

