# Supplementary material for: Octyl itaconate enhances VSVΔ51 oncolytic virotherapy by multitarget inhibition of antiviral and inflammatory pathways
Source: Nat Commun. 2024 May 15;15:4096. doi: 10.1038/s41467-024-48422-x (PMC11096414; doi:10.1038/s41467-024-48422-x)
Supplement: Supplementary file 1 — Supplementary Information [file 41467_2024_48422_MOESM1_ESM.pdf]

Octyl-Itaconate Enhances VSVΔ51 Oncolytic Virotherapy by Multitarget Inhibition of Antiviral and Inflammatory Pathways

Naziia Kurmasheva *et al.*

This PDF file includes:

- Supplementary Figures 1-19
- Supplementary Tables 1-6
- Supplementary Methods (Copies of NMR spectra)

Supplementary Figures 1-19

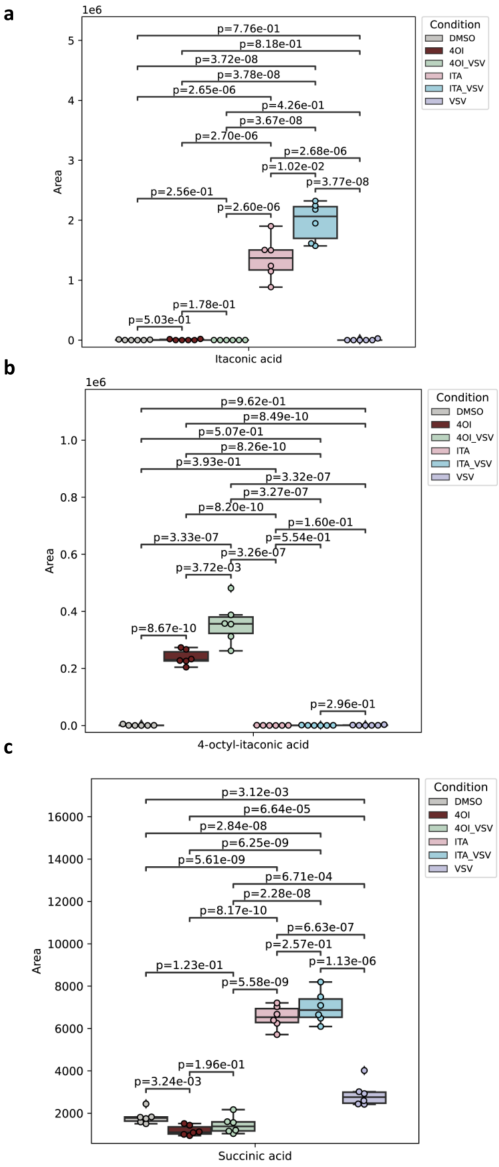

Figure S1. Modulation of metabolites production in 4-OI *vs* itaconate-treated and VSVΔ51-infected cancer cells

a-c 786-O cells were either pre-treated with 4-OI (75 µM) or itaconate (10 mM) for 24 hrs and subsequently infected with VSVΔ51 (MOI 0.01) for 17 hours. Metabolites were measured by LC mass spectrometry. Data come from one experiment performed in sextuplicate. Data are depicted as: each box shows the interquartile range (IQR) extending between [Q1, Q3], the line inside the box indicates the median value, the "whiskers" extend to points that lie within 1.5 IQRs of Q1 and Q3. Statistics indicate significance by two-tailed Student's t-test for **a-c**. Source data are provided as a Source Data file.

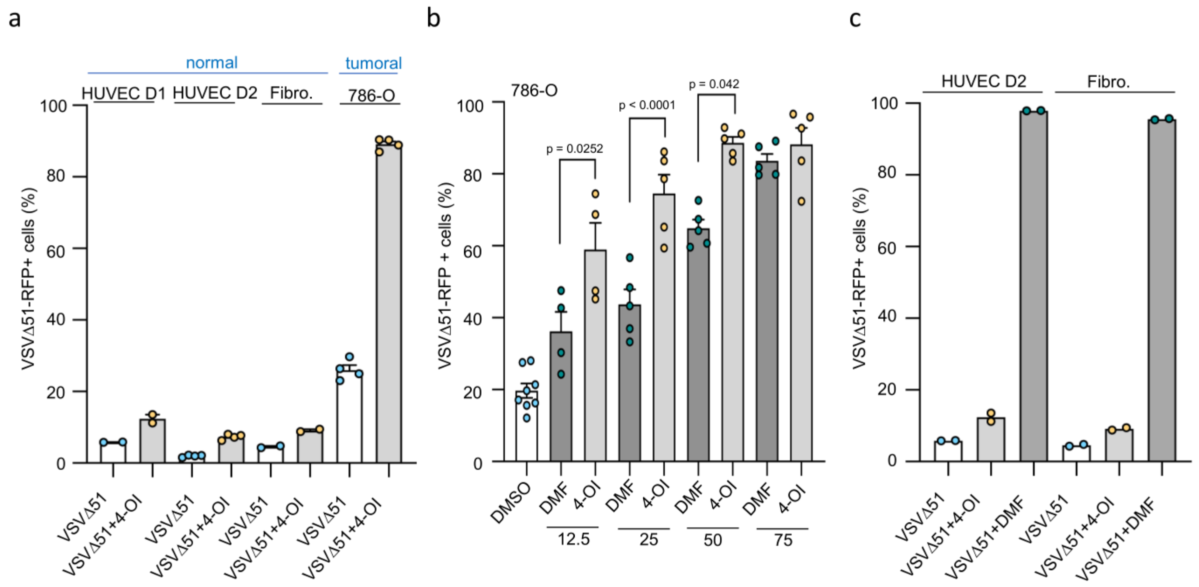

Figure S2. 4-OI outperforms the OV boosting capacity of dimethyl fumarate in vitro and does not enhance VSVΔ51 infection in non-tumoral cells

a Primary HUVECs from two different donors (D1 and D2), primary human fibroblasts and tumoral 786-O cells were pre-treated with 4-OI (125 µM) for 24 hours and subsequently infected with VSVΔ51 (MOI 0.01) for 17 hours. The number of infected RFP+ cells was quantified by flow cytometry.

b 786-O cells were pre-treated with the indicated concentrations of 4-OI or dimethyl fumarate (DMF) for 24 hours and subsequently infected with VSVΔ51 (MOI 0.01) for 17 hours. The number of infected RFP+ cells was quantified by flow cytometry.

c Primary HUVECs from donor 2 (D2), and primary human fibroblasts were pre-treated with 4-OI (125 µM) or DMF (100 µM) for 24 hours and subsequently infected with VSVΔ51 (MOI 0.01) for 17 hours. The number of infected RFP+ cells was quantified by flow cytometry. Data are depicted as means ± SEM from two experiments performed in duplicate in **a** and from two experiments performed in triplicate and quadruplicate in **b**. Data are depicted as means ± SEM from one experiment performed in duplicate in **c**. Statistics indicate significance by one-way ANOVA for **b**. Source data are provided as a Source Data file.

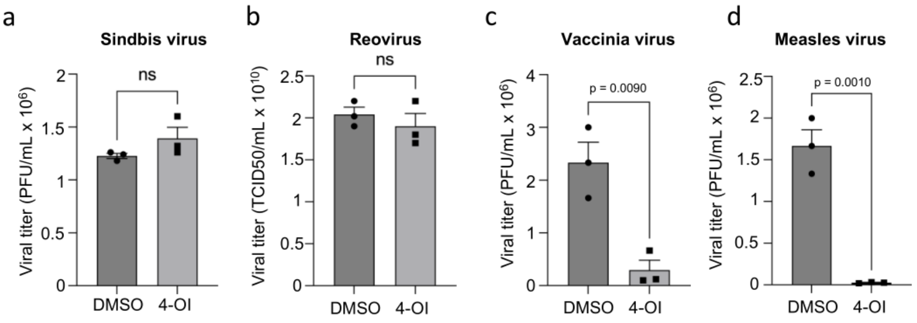

Figure S3. 4-OI restricts infection of oncolytic Vaccinia and Measles in cancer cells and has no effect on Sindbis and Reovirus infection

a 786-O cells were pre-treated with 4-OI (100 µM) for 24 hours and subsequently infected with Sindbis virus (MOI 10) for 48 hrs. Viral titers were determined in the supernatants of infected cells by plaque assay.

b-d PANC1 cells were pre-treated with 4-OI (125 µM) for 24 hours and subsequently infected with Reovirus (10^6 TCID50/ml), vaccinia virus (MOI 0.01) and Measles virus (MOI 0.1) for 48 hrs. Viral titers were determined in the supernatants of infected cells by plaque assay or TCID50. Data are depicted as means ± SEM from one experiment performed in triplicate in **a-d**. Statistics indicate significance by two-tailed Student's t-test for **a-d**. Source data are provided as a Source Data file.

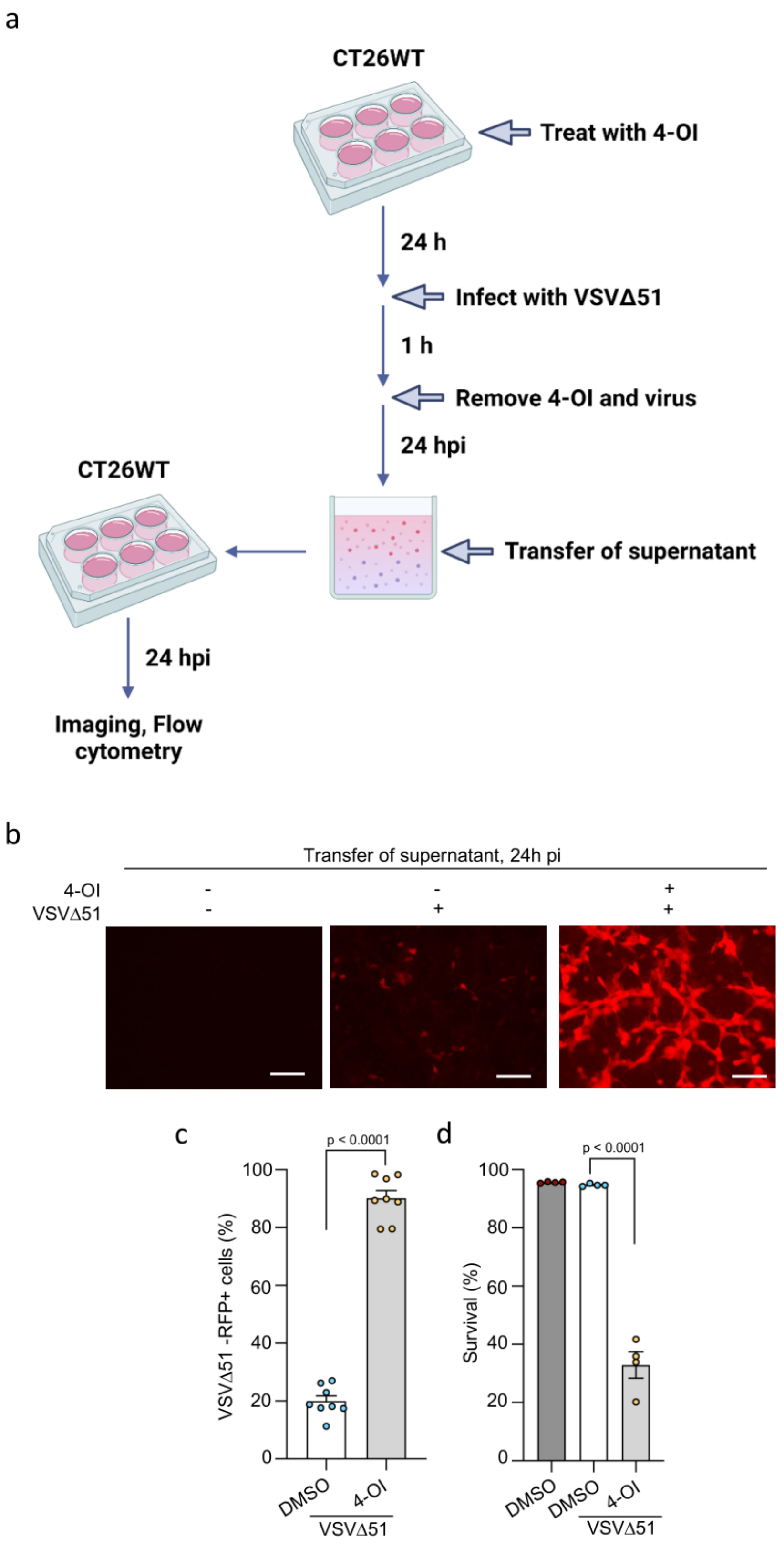
a
CT26WT
Treat with 4-OI
24 h
Infect with VSVΔ51
1 h
Remove 4-OI and virus
24 hpi
CT26WT
Transfer of supernatant
24 hpi
Imaging, Flow cytometry
b
Transfer of supernatant, 24h pi
4-OI - - +
VSVΔ51 - + +
c
p < 0.0001
VSVΔ51 -RFP+ cells (%)
100
80
60
40
20
0
DMSO
4-OI
VSVΔ51
d
p < 0.0001
Survival (%)
100
80
60
40
20
0
DMSO
DMSO
4-OI
VSVΔ51

Figure on previous page

Figure S4. Transfer of supernatants from virus-infected and 4-OI-treated cells can promote OV infection in bystander untouched cancer cells

a Schematic representing the layout of the supernatant transfer experiment. CT26WT cells were treated with 4-OI (125 μM) for 24hrs following 1h incubation with VSVΔ51 (MOI 0.01). The supernatants containing virus were washed off and replaced by fresh medium (not containing virus or 4-OI). 24 hrs later these media were transferred to freshly plated CT26WT cells for another 24 hrs. Infectivity and survival were assessed 24 hrs after incubation with the supernatants.

b-d Supernatants containing virus from 4-OI- or DMSO-stimulated CT26WT cells was transferred to bystander untouched cells for 24 hours. Infectivity was assessed by fluorescence microscopy (**b**) (Scale bars, 100 μm) or flow cytometry of RFP+ cells (**c**).

Data are depicted as means ± SEM from two experiments performed in quadruplicate in **c** and from two experiments performed in duplicate in **d**. Pictures in are representative of one experiment performed three times in **c**. Statistics indicate significance by two-tailed Student's t-test for **c** and one-way ANOVA in **d**. Panel **a** has been created using BioRender.com. Source data are provided as a Source Data file.

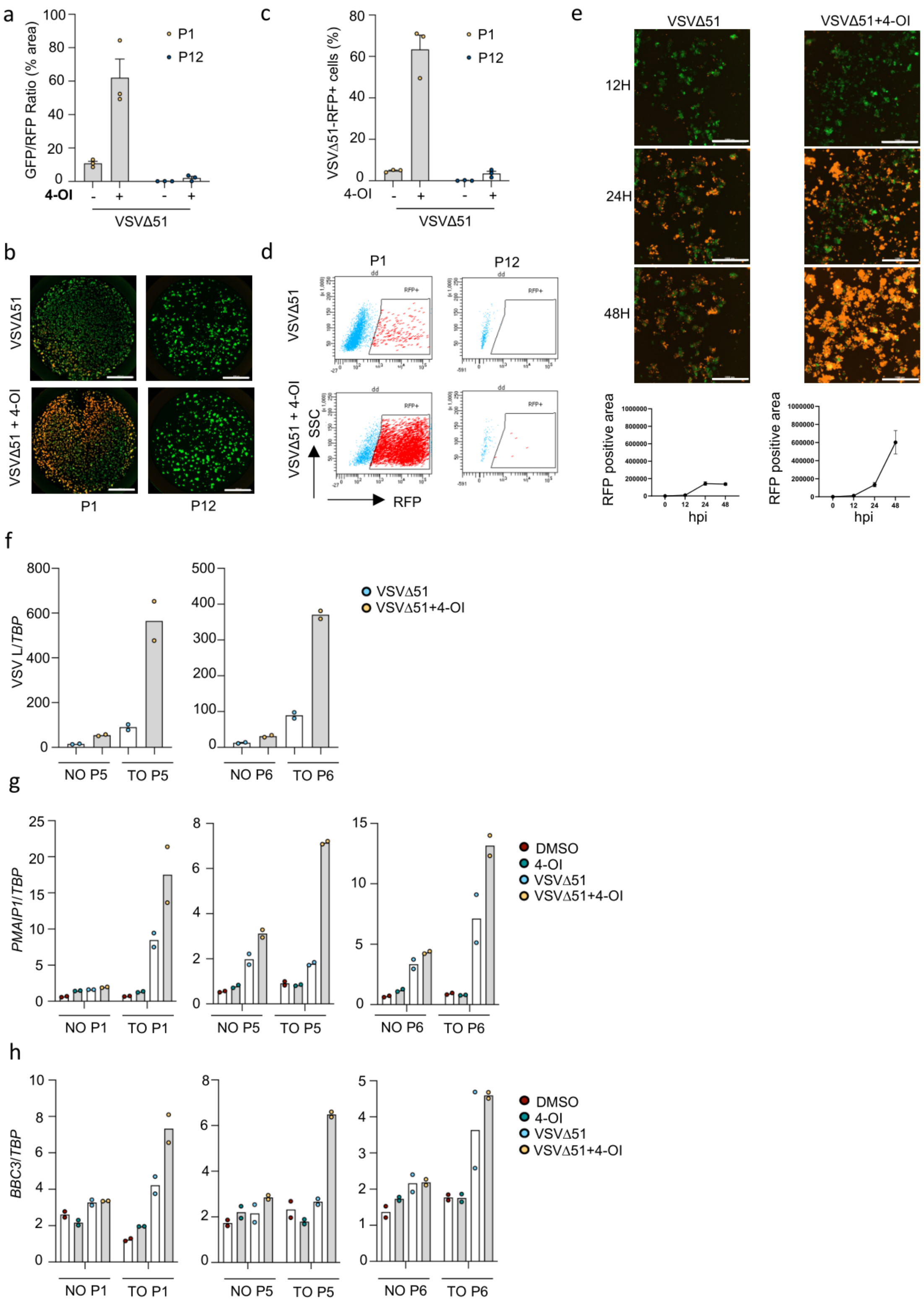
a
GFP/RFP Ratio (% area)
P1
P12
4-OI
VSVΔ51
b
VSVΔ51
VSVΔ51 + 4-OI
P1
P12
c
VSVΔ51-RFP+ cells (%)
P1
P12
4-OI
VSVΔ51
d
P1
P12
VSVΔ51
VSVΔ51 + 4-OI
SSC
RFP
RFP+
e
VSVΔ51
VSVΔ51+4-OI
12H
24H
48H
RFP positive area
hpi
f
VSV L/TBP
VSVΔ51
VSVΔ51+4-OI
NO P5
TO P5
NO P6
TO P6
g
PMAIP1/TBP
DMSO
4-OI
VSVΔ51
VSVΔ51+4-OI
NO P1
TO P1
NO P5
TO P5
NO P6
TO P6
h
BBC3/TBP
DMSO
4-OI
VSVΔ51
VSVΔ51+4-OI
NO P1
TO P1
NO P5
TO P5
NO P6
TO P6

Figure on previous page

Figure S5. 4-OI increases oncolytic infectivity and killing by VSVΔ51 in 3D patient-derived colon cancer tumoroids

a-b Fluorescence area quantification (48-well plate) to evaluate the infectivity of VSVΔ51-RFP infection (1×10^6 pfu/well) solely, and in combination with 4-OI (125 μM), within colon cancer organoids derived from two patients (P1 and P12) 48 hours post infection. To assess for total organoid area, organoids were either transduced with GFP or stained with calcein green. Scale bars, 3000 μm.

c-d Flow cytometry counts of infected RFP-positive cells from enzymatically digested colon tumoroids in patient 1 and 12 (P1 and P12) at 48 hours post infection with VSVΔ51 infection (1×10^6 pfu/well) in the presence or not of 4-OI (125 μM).

e RFP area signal from VSVΔ51-RFP (1×10^6 pfu/well) to evaluate virus infection solely or in combination with 4-OI (125 μM), within colon cancer organoids derived from one patient (P1) at different time post infection. Scale bars, 1000 μm.

f qPCR analysis of VSV L gene expression within colon NO (normal) and TO (tumoral) organoids from patient 5 (P5) and patient 6 (P6), one day post infection with VSVΔ51 (1×10^6 pfu/well) in the presence or not of 4-OI (125 μM)

g-h qPCR analysis of NOXA (*PMAIP1*) (g) and PUMA (*BBC3*) (h) gene expression within colon NO (normal) and TO (tumoral) organoids from different patients (P1, P5 and P6), one day post infection with VSVΔ51 ($1\text{x}10^6$ pfu/well) in the presence or not of 4-OI (125 μM). Data are depicted as means ± SEM from one experiment performed in biological triplicates from two patients in **a,b**, from one experiment performed in biological triplicates from two patients in **c-d**, from one experiment on one patient in **e**, from one experiment performed in biological duplicates in two to three patients in **f-h**. Source data are provided as a Source Data file.

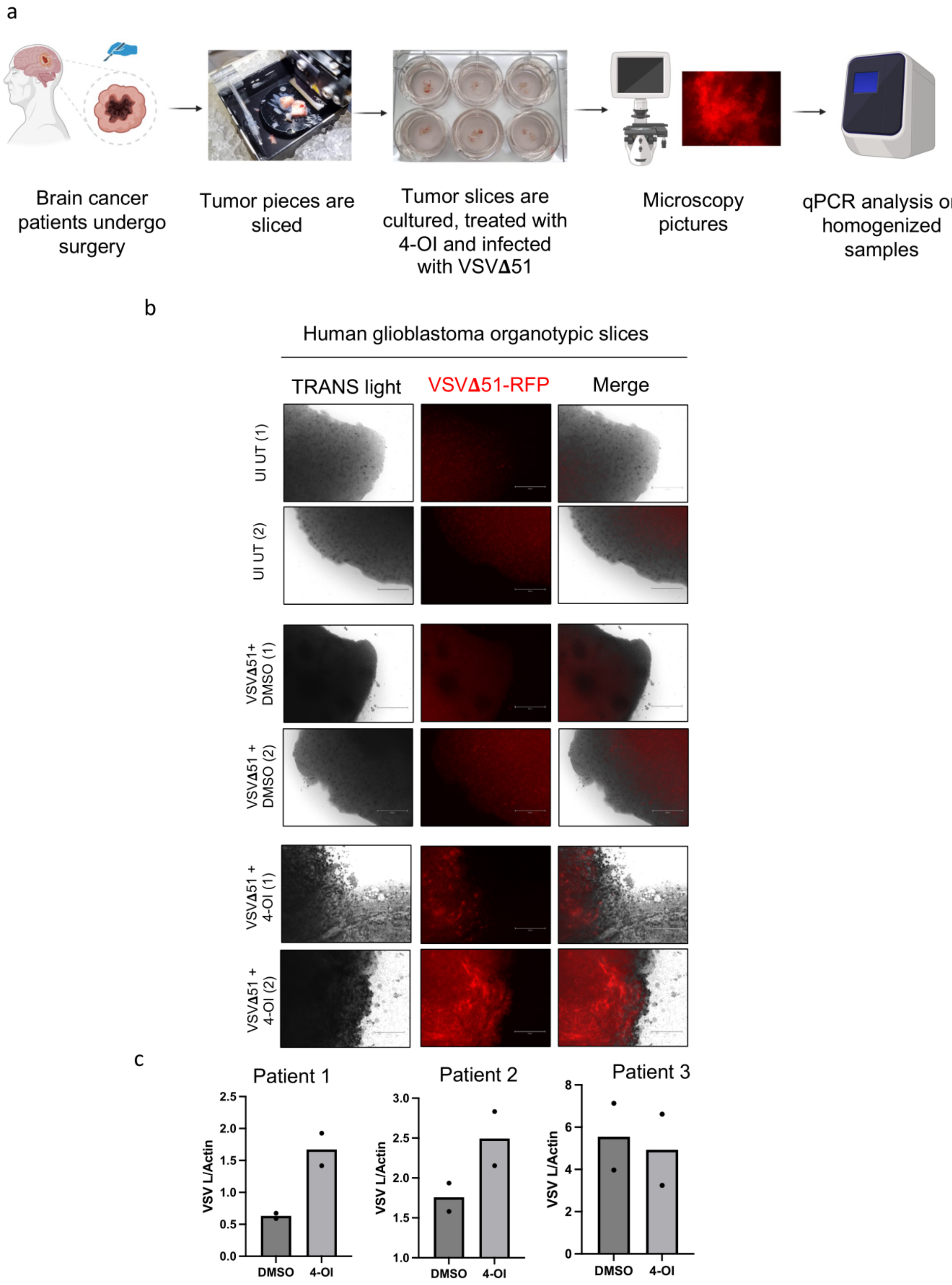
a
Brain cancer patients undergo surgery
Tumor pieces are sliced
Tumor slices are cultured, treated with 4-OI and infected with VSVΔ51
Microscopy pictures
qPCR analysis on homogenized samples
b
Human glioblastoma organotypic slices
TRANS light
VSVΔ51-RFP
Merge
UI UT (1)
UI UT (2)
VSVΔ51+ DMSO (1)
VSVΔ51 + DMSO (2)
VSVΔ51 + 4-OI (1)
VSVΔ51 + 4-OI (2)
c
Patient 1
Patient 2
Patient 3
VSV L/Actin
DMSO
4-OI
0.0
0.5
1.0
1.5
2.0
2.5
3.0
0
2
4
6
8

Figure on previous page

Figure S6. 4-OI increases VSVΔ51 in patient-derived organotypic brain cancer slices
a Schematic highlighting the procedure of the experiment.
b Organotypic glioblastoma slices were infected with VSVΔ51-RFP (4×10^6 PFU) in the presence of 4-OI (125 μM) for 20 hrs. Infectivity of the slices was assessed by fluorescence imaging. Scale bars, 300 μm.
c qPCR analysis of VSV L gene expression within the tumor slices from four patients; 20 hours post infection with VSVΔ51 (4×10^6 PFU) in the presence or not of 4-OI (125 μM) (P1), 40 hours post infection with VSVΔ51 (8×10^6 PFU) in the presence or not of 4-OI (125 μM) (P2-P3). Data are from one experiment performed on three different patients (**Table S2**). For the qPCR, experiments were done in biological duplicates and display the means. Panel **a** has been created using BioRender.com. Source data are provided as a Source Data file.

a

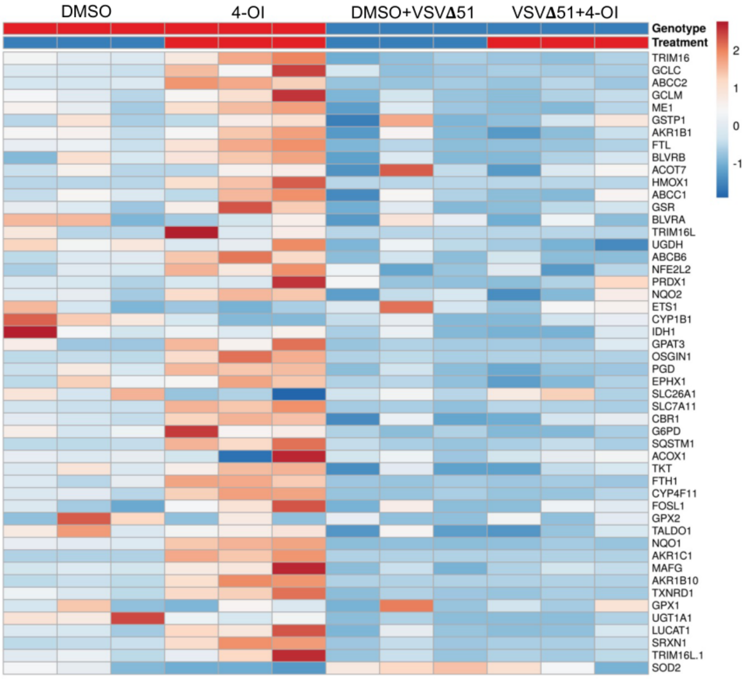
DMSO
4-OI
DMSO+VSVΔ51
VSVΔ51+4-OI
Genotype
Treatment
2
1
0
-1
TRIM16
GCLC
ABCC2
GCLM
ME1
GSTP1
AKR1B1
FTL
BLVRB
ACOT7
HMOX1
ABCC1
GSR
BLVRA
TRIM16L
UGDH
ABCB6
NFE2L2
PRDX1
NQO2
ETS1
CYP1B1
IDH1
GPAT3
OSGIN1
PGD
EPHX1
SLC26A1
SLC7A11
CBR1
G6PD
SQSTM1
ACOX1
TKT
FTH1
CYP4F11
FOSL1
GPX2
TALDO1
NQO1
AKR1C1
MAFG
AKR1B10
TXNRD1
GPX1
UGT1A1
LUCAT1
SRXN1
TRIM16L.1
SOD2

b

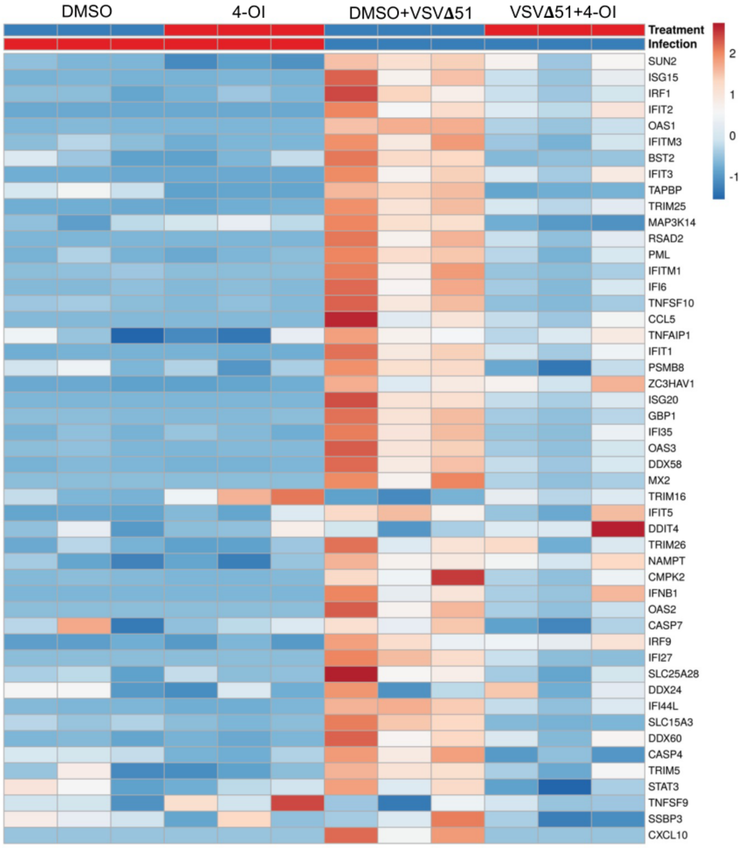
DMSO
4-OI
DMSO+VSVΔ51
VSVΔ51+4-OI
Treatment
Infection
2
1
0
-1
SUN2
ISG15
IRF1
IFIT2
OAS1
IFITM3
BST2
IFIT3
TAPBP
TRIM25
MAP3K14
RSAD2
PML
IFITM1
IFI6
TNFSF10
CCL5
TNFAIP1
IFIT1
PSMB8
ZC3HAV1
ISG20
GBP1
IFI35
OAS3
DDX58
MX2
TRIM16
IFIT5
DDIT4
TRIM26
NAMPT
CMPK2
IFNB1
OAS2
CASP7
IRF9
IFI27
SLC25A28
DDX24
IFI44L
SLC15A3
DDX60
CASP4
TRIM5
STAT3
TNFSF9
SSBP3
CXCL10

Figure on previous page

Figure S7. Heatmap of differentially expressed antiviral and NRF2-regulated genes in 4-OI-stimulated NRF2 KO cancer cells

a-b 786-O cells were pre-treated with 4-OI (75 μM) for 24 hours and subsequently infected with VSVΔ51 (MOI 0.01) for 17 hours. RNA was analyzed using RNA sequencing. Heat map showing the expression of differentially expressed NRF2-regulated genes (**a**) and interferon stimulated genes (ISGs) (**b**) across all experimental conditions. Data are from one experiment performed in triplicate.

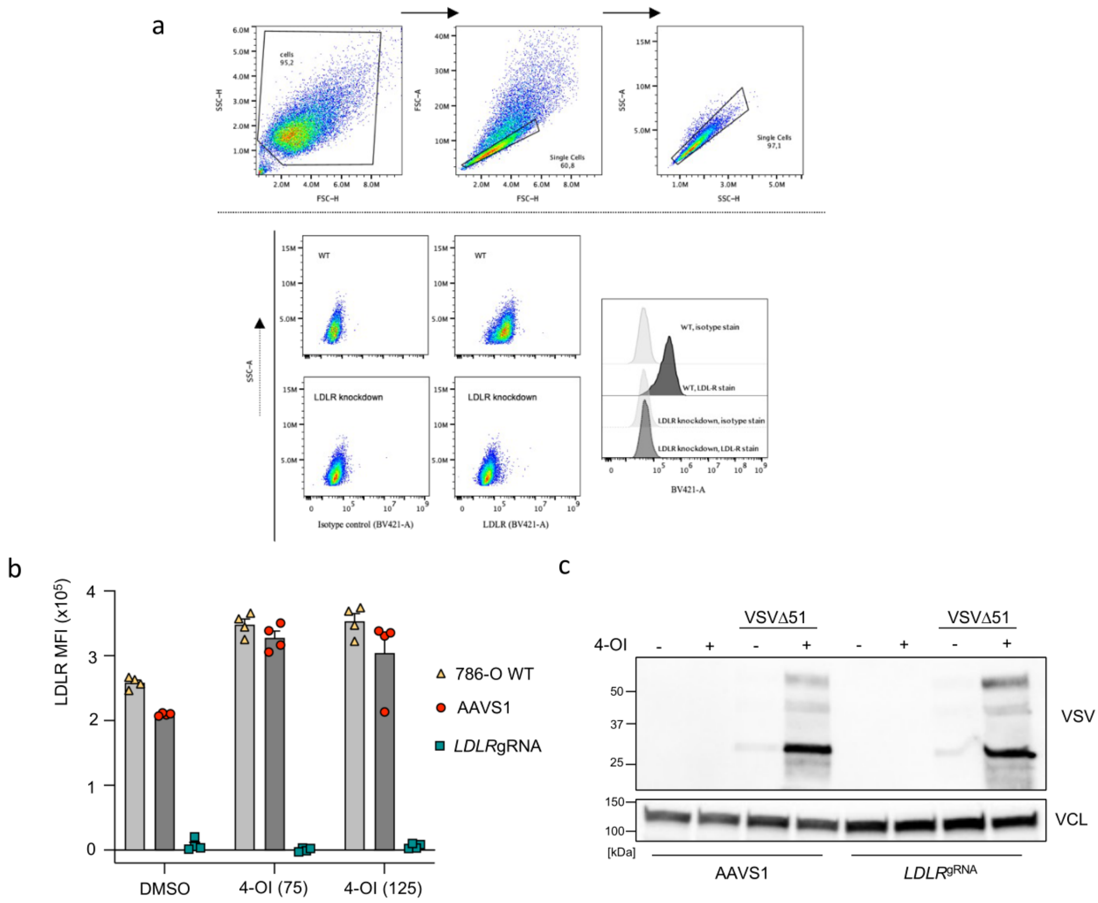

Figure S8. 4-OI does not promote OV infectivity in cancer cells via the modulation of LDL Receptor Surface Levels

a Representative flow cytometry plots showing the gating strategy used to determine the LDLR expression. Total cells were selected using SSC-H/FSC-H gating, followed by FSC-A/FSC-H and SSC-A/SSC-H to select for single cells, which were used to measure the BV421 fluorescence upon staining with LDLR-BV421 antibody or the respective BV421-conjugated isotype control.

b 786-O cells were transiently KO for LDL Receptor (LDLR) (*LDLR*^{gRNA}) using CRISPR/Cas9 gene editing, treated with a control gRNA sequence (AAVS1) or simply electroporated (786-O WT). Following CRISPR/Cas9 gene editing, cells were treated with 4-OI (75 or 125 µM) for 24 hours and LDLR surface levels was assessed by flow cytometry.

c 786-O cells transiently KO for LDL Receptor (LDLR) (*LDLR*^{gRNA}) using CRISPR/Cas9 gene editing were pre-treated with 4-OI (75 µM) for 24 hours and subsequently infected with VSVΔ51 (MOI 0.01) for 17 hours. Western blot was performed on cell lysates for VSV proteins. Vinculin (VCL) was used as a housekeeping control. Data are depicted as means ± SEM from two experiments performed in duplicate in **a**-**b**. WB is representative of one experiment performed twice in **c**. Source data are provided as a Source Data file.

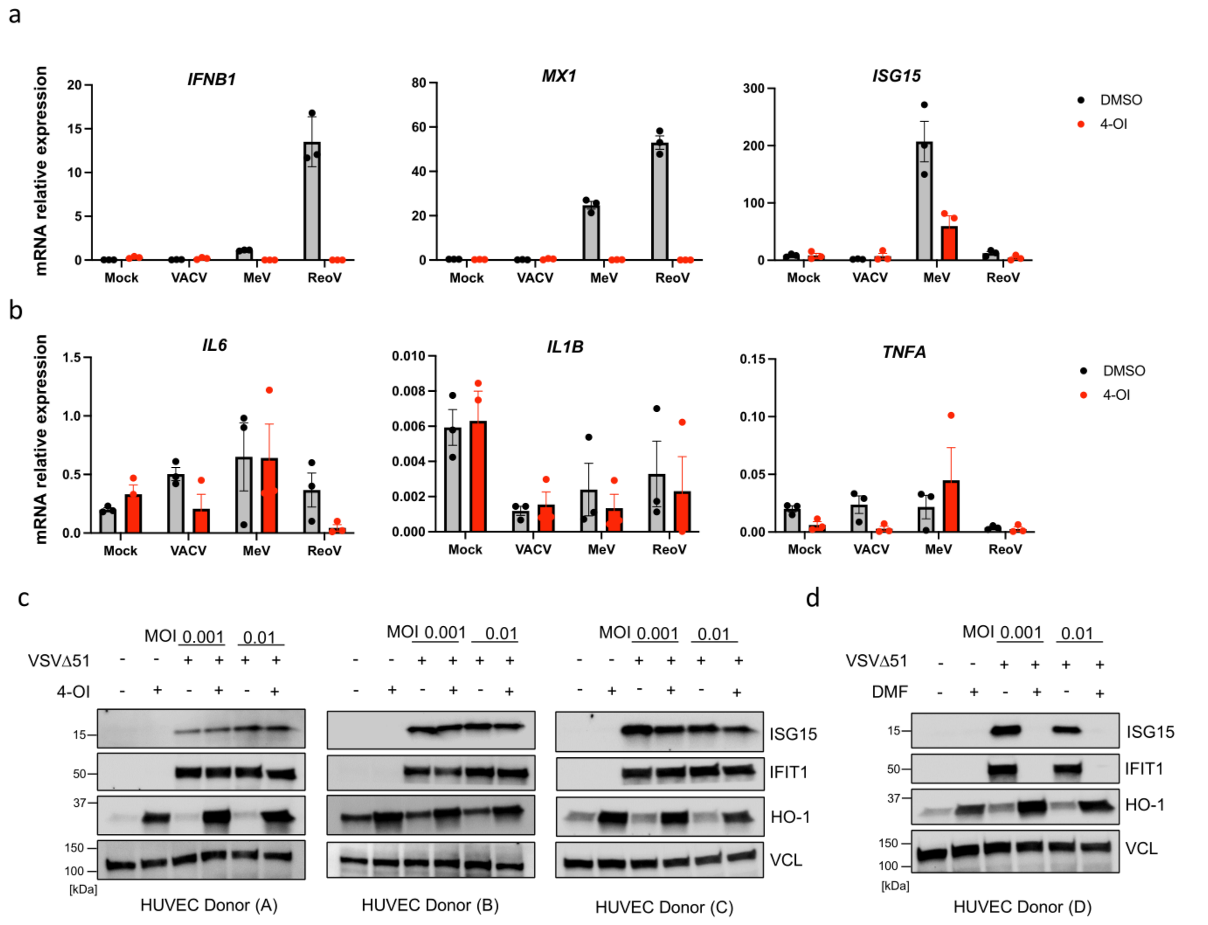

Figure S9. Modulation of antiviral immune responses by 4-OI in tumoral and normal cells

a-b PANC1 cells were pre-treated with 4-OI (125 μM) for 24 hours and subsequently infected with Reovirus (10^6 TCID50/ml), vaccinia virus (MOI 0.01) and Measles virus (MOI 0.1) for 48 hrs. Antiviral and inflammatory gene levels were determined by qPCR.

c-d Primary HUVECs from four different donors (Donor A to Donor D) were pre-treated with 4-OI (125 μM) (c) or DMF (100 μM) (d) for 24 hours and subsequently infected with VSVΔ51 (MOI 0.001 or MOI 0.01) for 17 hours. Antiviral immune responses were assessed by immunoblotting of IFIT1 and ISG15. HO-1 served as a positive control of NRF2 induction and VCL was using as an invariant loading control. Data are depicted as means ± SEM from one experiment performed in triplicates in **a**-**b**. Vertical stacks of bands are not derived from the same membrane in **c** and **d**. Source data are provided as a Source Data file.

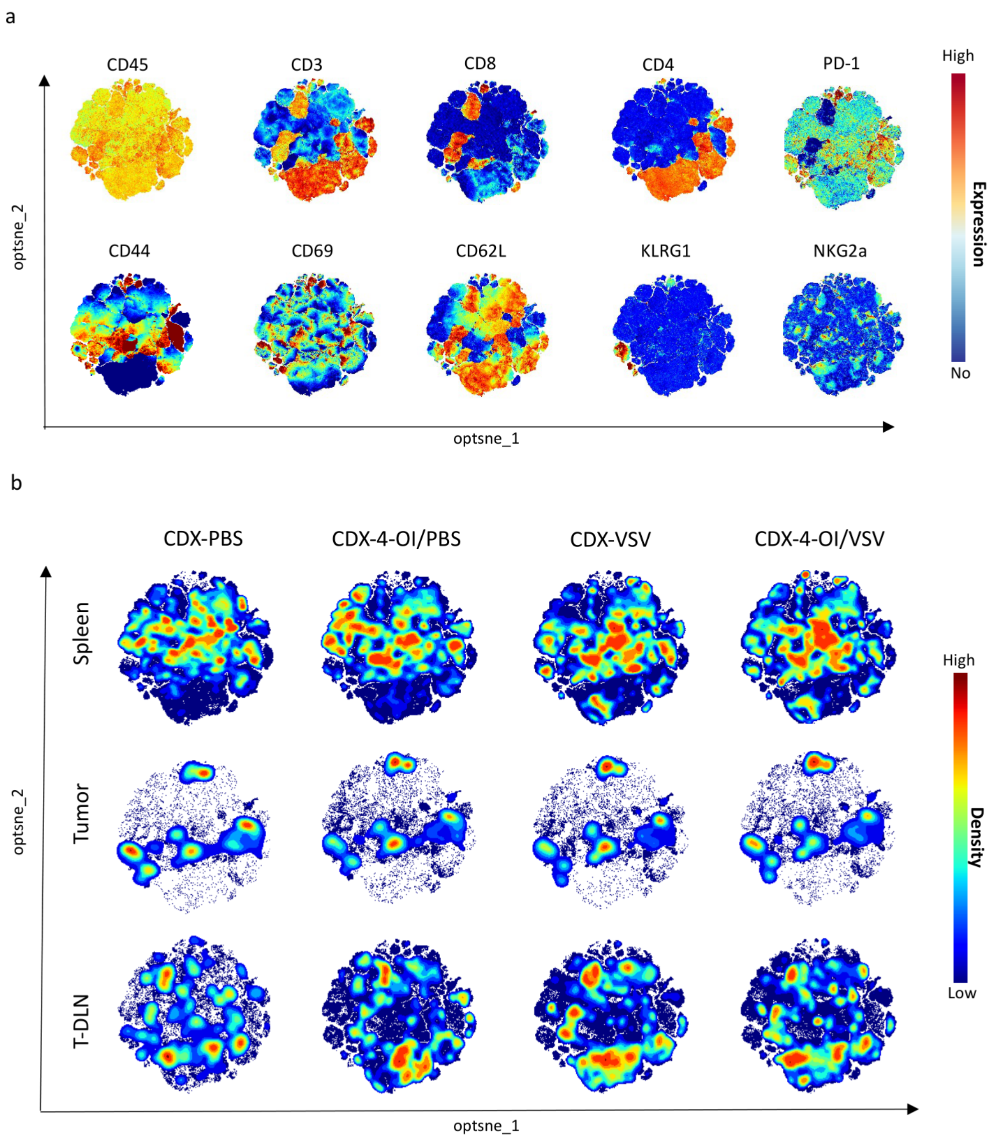

Figure S10. 4-OI and/or VSVΔ51 does not affect the T-cell landscape within the tumor and lymphoid organs

Flow cytometry data of single cell suspensions isolated from tumor, spleen or tumor-draining lymph node (T-DLN) isolated five days after the last VSVΔ51 injection. Data was analyzed using OMIQ software.

a Expression intensity profile of T-cell markers on clustered live, CD45+ gated samples of all treatment groups and organs (n=57) to distinguish regional expression of single T-cell markers in clusters. Relative expression is indicated by color where red indicates high expression and blue represents no expression within the cluster.

b OptSNE cluster plots of live, CD45+ gated samples displayed per organ and treatment group (n=3 mice in CDX-PBS group; n=5 mice in CDX-4-OI/PBS group, n=6 mice in CDX-VSV, and n=6 mice in CDX-4-OI/VSV groups). Cell density is indicated by color where red indicates high density and blue indicates low density within the cluster. 3 individual samples were lost during acquisition: 1 spleen sample in CDX-4-OI/VSV group (n=5), 1 tumor sample in CDX/VSV group (n=5), and 1 T-DLN sample in CDX/VSV group (n=5).

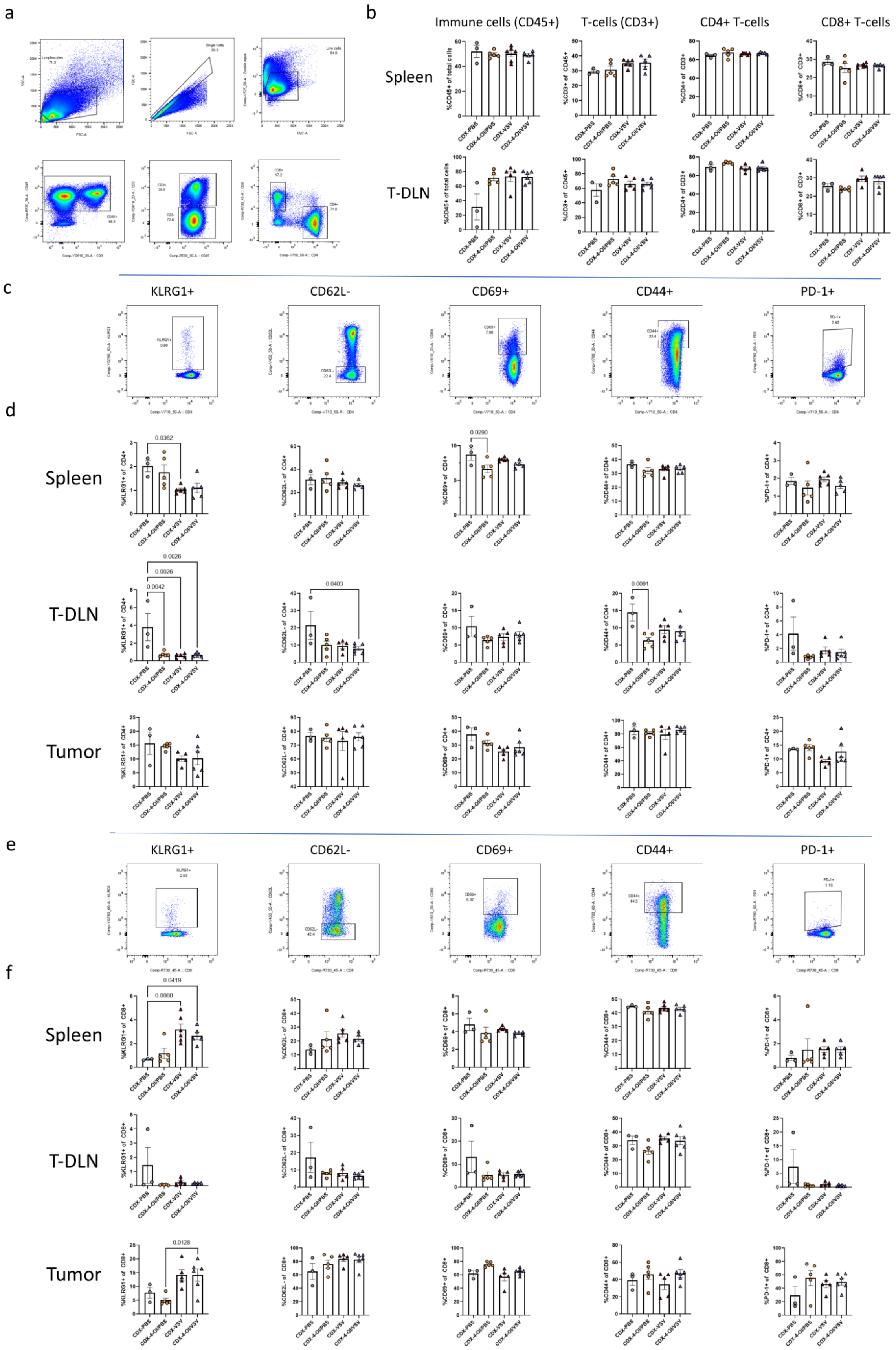

a
b
Immune cells (CD45+)
T-cells (CD3+)
CD4+ T-cells
CD8+ T-cells
Spleen
T-DLN
c
KLRG1+
CD62L-
CD69+
CD44+
PD-1+
d
Spleen
T-DLN
Tumor
0.0362
0.0290
0.0026
0.0026
0.0042
0.0403
0.0091
e
KLRG1+
CD62L-
CD69+
CD44+
PD-1+
f
Spleen
T-DLN
Tumor
0.0419
0.0060
0.0128
CDX-PBS
CDX-4-OI/PBS
CDX-VSV
CDX-4-OI/VSV

Figure on previous page

Figure S11. 4-OI and/or VSVΔ51 do not affect T-cell density and phenotype in tumor and lymphoid organs

Flow cytometry analysis of single cell suspensions isolated from tumor, spleen or tumor-draining lymph node (T-DLN) five days after the last VSVΔ51 injection. Data was analyzed using FlowJo software.

a Representative gating strategy for T-cell populations showed for spleen starting from total cells.

b Mean percentage of indicated T-cell population compared between treatment groups (n=3-6 animals per group). Each dot represents one mouse. n=3 mice in CDX-PBS group; n=5 mice in CDX-4-OI/PBS group, n=6 mice in CDX-VSV, and n=6 mice in CDX-4-OI/VSV groups. 3 individual samples were lost during acquisition: 1 spleen sample in CDX-4-OI/VSV group (n=5), 1 tumor sample in CDX/VSV group (n=5), and 1 T-DLN sample in CDX/VSV group (n=5).

c Representative gating strategy for activation markers on CD4+ T-cells showed for spleen.

d Mean percentage of CD4+ T-cells expressing different activation markers in spleen, T-DLN and tumor, compared between treatment groups (n=3-6 animals per group).

e Representative gating strategy for activation markers on CD8+ T-cells showed for spleen.

f Mean percentage of CD8+ T-cells expressing different activation markers in spleen, T-DLN and tumor, compared between treatment groups (n=3-6 animals per group).

P-value is calculated with one-way ANOVA followed by Šídák's multiple comparisons test. No difference found, unless P-value indicated. Source data are provided as a Source Data file.

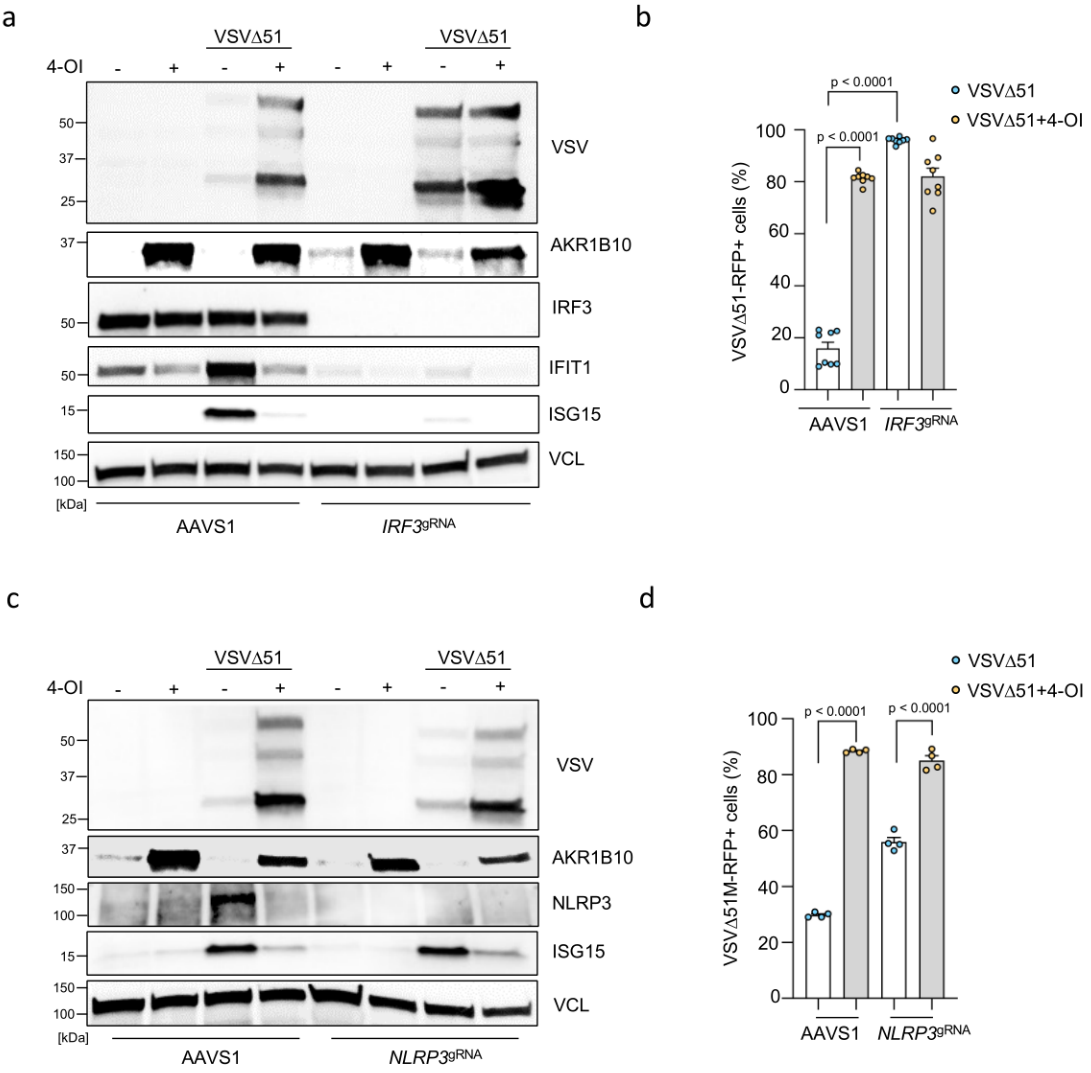

Figure S12. 4-OI does not promote OV infectivity in cancer cells via the alkylation of NLRP3

a-b 786-O cells transiently KO for IRF3 (*IRF3*^{gRNA}) using CRISPR/Cas9 gene editing were pre-treated with 4-OI (75 µM) for 24 hours and subsequently infected with VSVΔ51 (MOI 0.01) for 17 hours. Western blot was performed on cell lysates for VSV proteins and antiviral markers (a). Vinculin (VCL) was used as a housekeeping control. The number of infected RFP+ cells was quantified by flow cytometry (b).

c-d 786-O cells transiently KO for NLRP3 (*NLRP3*^{gRNA}) using CRISPR/Cas9 gene editing were pre-treated with 4-OI (75 µM) for 24 hours and subsequently infected with VSVΔ51 (MOI 0.01) for 17 hours. Western blot was performed on cell lysates for VSV proteins and antiviral markers (c). Vinculin (VCL) was used as a housekeeping control. The number of infected RFP+ cells was quantified by flow cytometry (d).

Data are depicted as means ± SEM from two experiments performed in quadruplicate in **a** and duplicate in **b**. WB is representative of one experiment performed twice in **a** and **b**. Statistics indicate significance by one-way ANOVA in **b** and **d**. Vertical stacks of bands are not derived from the same membrane in **a** and **c.** Source data are provided as a Source Data file.

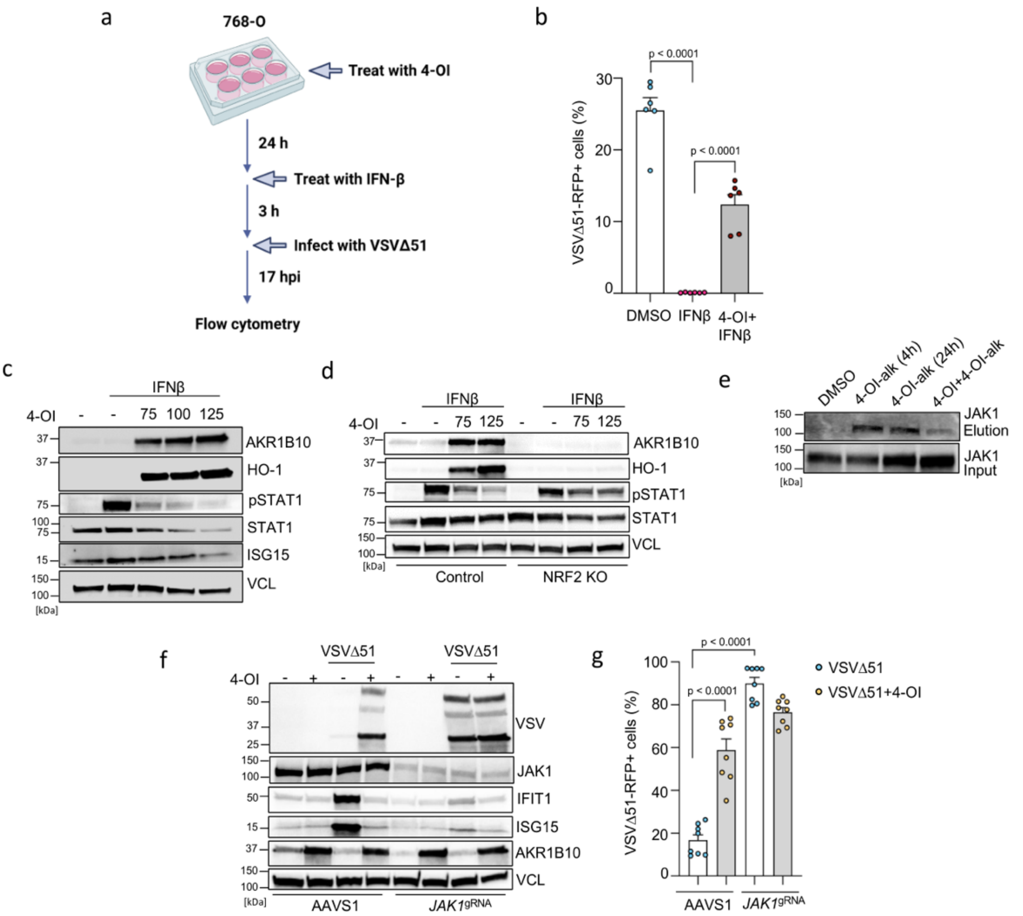

Figure S13. 4-OI enhances VSVΔ51 infectivity in cancer cell via the impairment of type IFN signaling through JAK1 direct modification

a-b 786-O cells were treated with 4-OI (75 µM) for 24 hours prior to IFNβ stimulation (200 U/mL) for 3 hours. Cells were subsequently infected with VSVΔ51 (MOI 0.01) for 17 hours (a) and infectivity was assessed by flow cytometry of infected RFP+ cells.

c-d 786-O cells (c) or control and NRF2 KO 786-O cells (d) were treated with 4-OI at the indicated concentrations for 24 hours prior to IFNβ stimulation (250 U/mL) for 30min. Cell lysates were immunoblotted for antiviral proteins and NRF2-related markers. Vinculin (VCL) was used as the invariant housekeeping control.

e 786-O cells were treated with 75 µM of alkynated 4-OI (4-OI-alk) for 4 or 24 hours in the presence or not of 4-OI (75 µM). The samples before and after enrichment were analyzed by anti-JAK1 immunoblotting.

f-g 786-O cells transiently KO for JAK1 (*JAK1*^{gRNA}) using CRISPR/Cas9 gene editing were pre-treated with 4-OI (75 µM) for 24 hours and subsequently infected with VSVΔ51 (MOI 0.01) for 17 hours. Western blot was performed on cell lysates for VSV proteins and antiviral markers. Vinculin (VCL) was used as a housekeeping control (f). The number of infected RFP+ cells was quantified by flow cytometry (g).

Data are depicted as means ± SEM from two experiments performed in triplicate in **b**. WB is representative of one experiment performed twice in **c-f**. Data are depicted as means ± SEM from two experiments performed in quadruplicate in **g**. Statistics indicate significance by one-way ANOVA in **b** and **g**.Vertical stacks of bands are not derived from the same membrane in **c, d** and **f**. Source data are provided as a Source Data file.

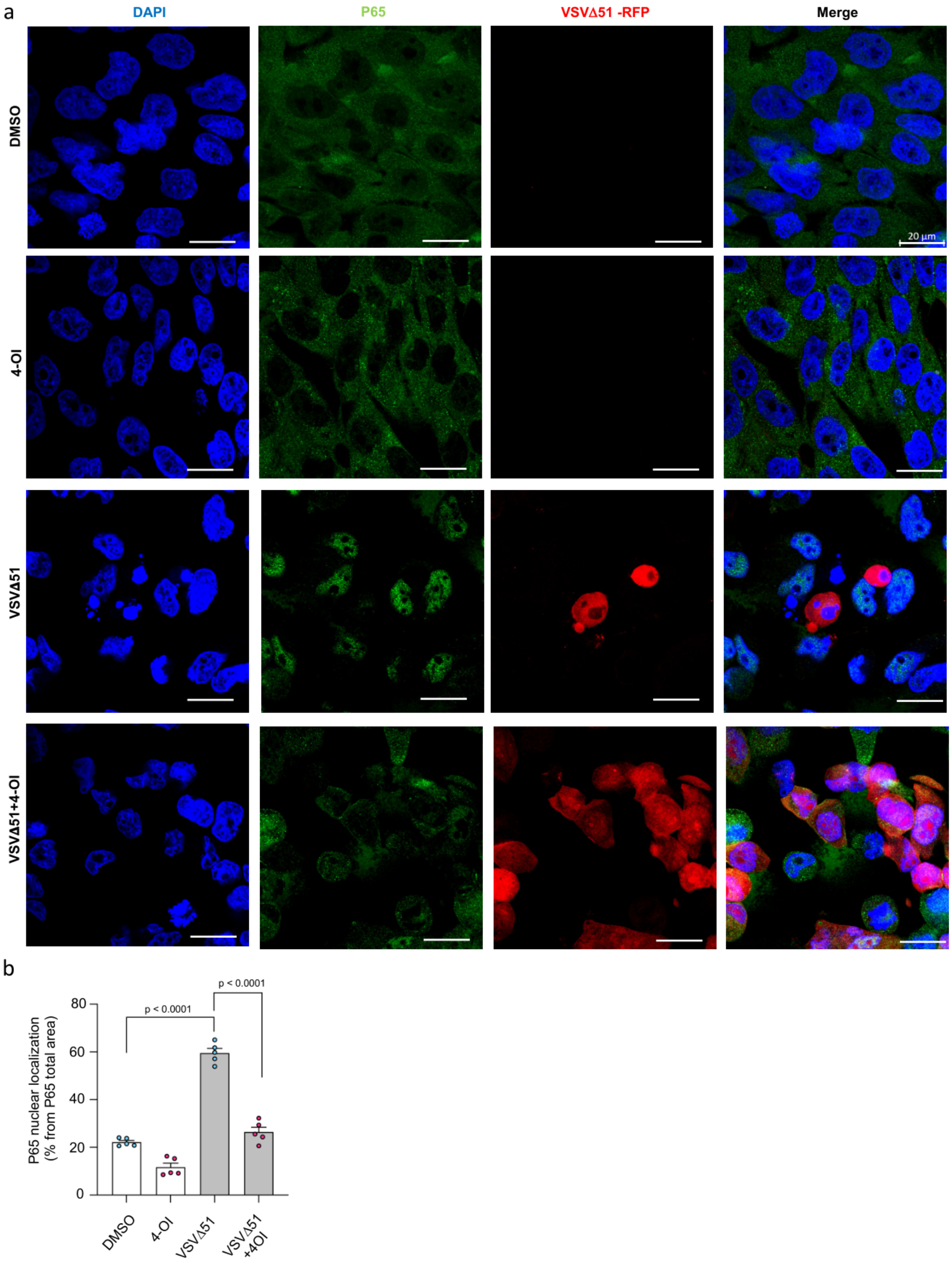

Figure S14. 4-OI prevents the nuclear translocation of RELA upon VSVΔ51 infection
a 786-O cells were pre-treated with 4-OI (75 µM) for 24 hours and subsequently infected with VSVΔ51 (MOI 0.01) for 17 hours. NF-κB p65 (RELA) nuclear translocation was assessed by fluorescence microscopy. Nuclei are stained with DAPI. Scale bars, 20 µm.
b p65 area within nuclear fraction was quantified using Image J software.
The data are representative of one experiment which has been repeated twice in **a**. Data are depicted as means ± SEM from one experiment performed in five replicates in **b**. Statistics indicate significance by one-way ANOVA in **b**. Source data are provided as a Source Data file.

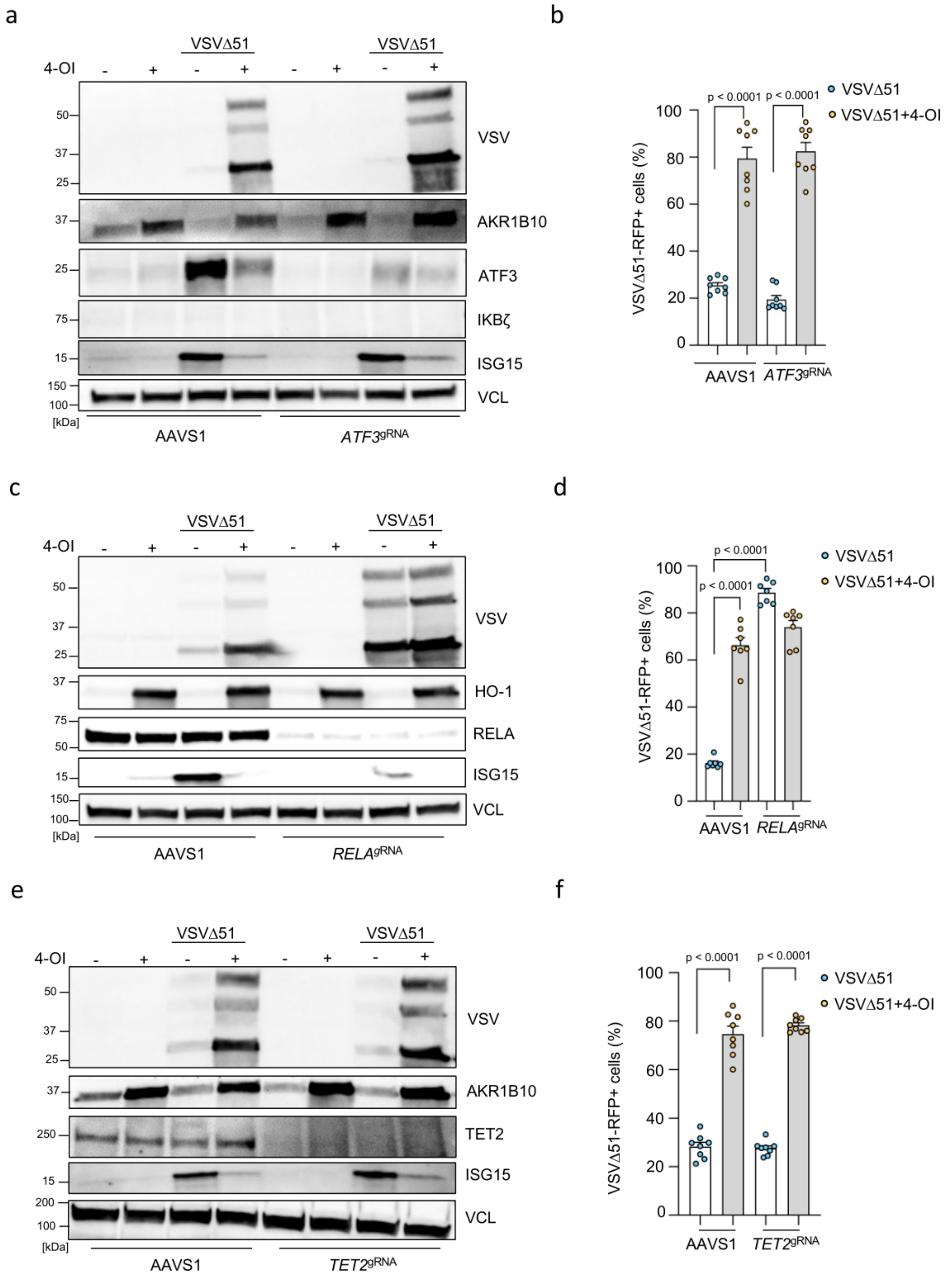
a
b
c
d
e
f
VSVΔ51
4-OI
VSV
AKR1B10
ATF3
IKBζ
ISG15
VCL
HO-1
RELA
TET2
[kDa]
AAVS1
ATF3gRNA
RELAgRNA
TET2gRNA
VSVΔ51-RFP+ cells (%)
p < 0.0001
VSVΔ51+4-OI

Figure on previous page

Figure S15. 4-OI does not promote OV infectivity in cancer cells via the alkylation of TET2 or the modulation of the ATF3-IKb ζ axis

a-b 786-O cells transiently KO for ATF3 (*ATF3*^{gRNA}) using CRISPR/Cas9 gene editing were pre-treated with 4-OI (75 µM) for 24 hours and subsequently infected with VSVΔ51 (MOI 0.01) for 17 hours. Western blot was performed on cell lysates for VSV proteins and antiviral markers (a). Vinculin (VCL) was used as a housekeeping control. The number of infected RFP+ cells was quantified by flow cytometry (b).

c-d 786-O cells transiently KO for p65/RELA (*RELA*^{gRNA}) using CRISPR/Cas9 gene editing were pre-treated with 4-OI (75 µM) for 24 hours and subsequently infected with VSVΔ51 (MOI 0.01) for 17 hours. Western blot was performed on cell lysates for VSV proteins and antiviral markers (c). Vinculin (VCL) was used as a housekeeping control. The number of infected RFP+ cells was quantified by flow cytometry (d).

e-f 786-O cells transiently KO for TET2 (*TET2*^{gRNA}) using CRISPR/Cas9 gene editing were pre-treated with 4-OI (75 µM) for 24 hours and subsequently infected with VSVΔ51 (MOI 0.01) for 17 hours. Western blot was performed on cell lysates for VSV proteins and antiviral markers (e). Vinculin (VCL) was used as a housekeeping control. The number of infected RFP+ cells was quantified by flow cytometry (f).

Data are depicted as means ± SEM from two experiments performed in quadruplicate in **b**, from two experiments in triplicate and quadruplicate in **d**, from two experiments in quadruplicate in **f**. WB is representative of one experiment performed twice in **a, c** and **e**. Statistics indicate significance by one-way ANOVA in **b, d** and **f**.Vertical stacks of bands are not derived from the same membrane in **a, c** and **e.** Source data are provided as a Source Data file.

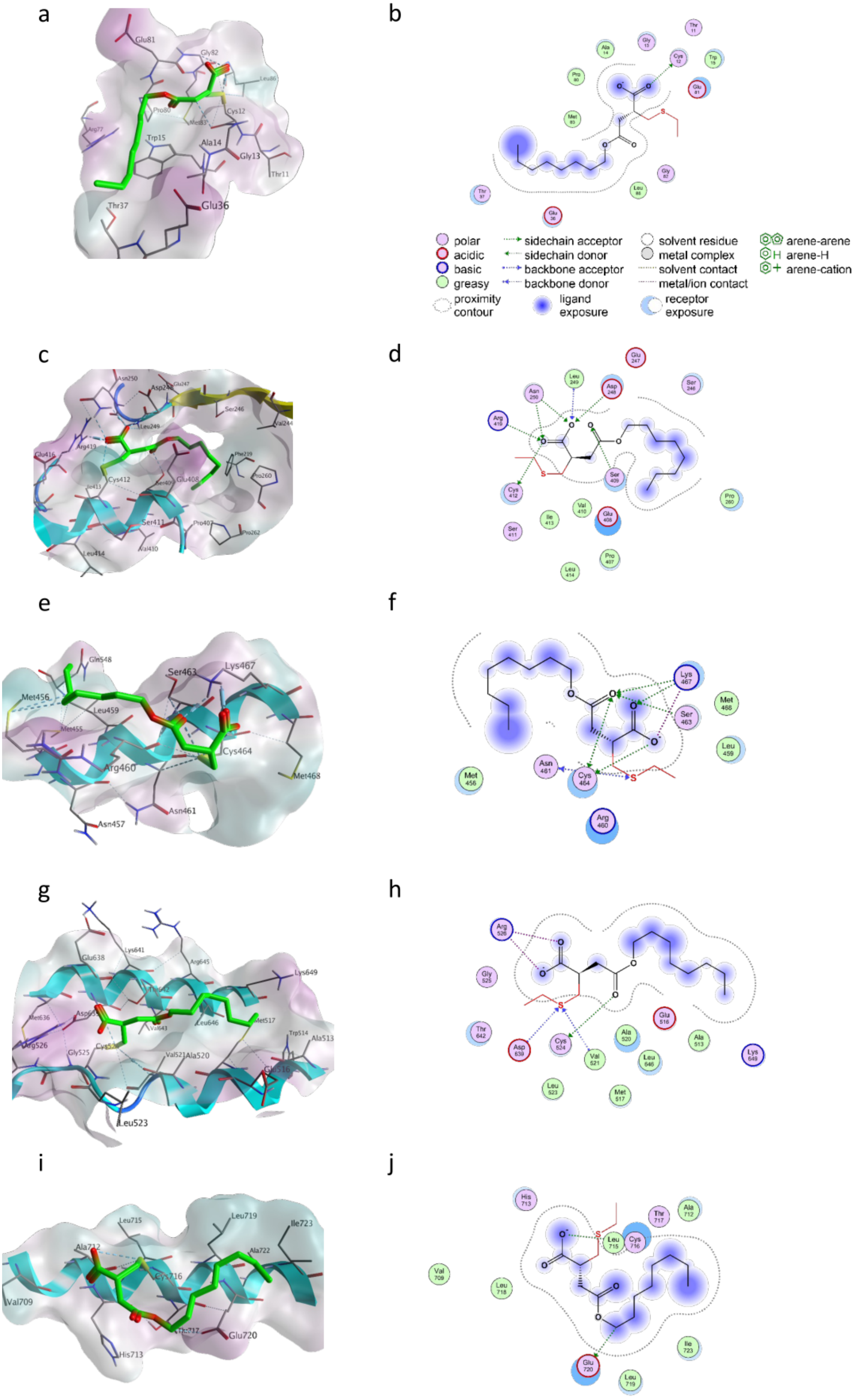
a
b
c
d
e
f
g
h
i
j
Glu81
Gly82
Pro80
Cys12
Met83
Trp15
Ala14
Gly13
Thr11
Thr37
Glu36
polar
acidic
basic
greasy
proximity contour
sidechain acceptor
sidechain donor
backbone acceptor
backbone donor
ligand exposure
solvent residue
metal complex
solvent contact
metal/ion contact
receptor exposure
arene-arene
arene-H
arene-cation
Asn250
Asp248
Glu247
Ser246
Leu249
Arg419
Glu416
Phe219
Cys412
Pro260
Glu408
Ser411
Pro407
Leu414
Val410
Gln548
Ser463
Lys467
Met456
Leu459
Met455
Cys464
Arg460
Met468
Asn457
Asn461
Glu638
Lys641
Arg645
Lys649
Met636
Leu646
Met517
Trp514
Ala513
Gly525
Val521
Ala520
Leu523
Leu715
Leu719
Ile723
Ala712
Ala722
Cys716
Val709
Glu720
His713

Figure on previous page

Figure S16. Potential covalent binding modes of 4-OI to cysteine residues in human IKKβ
a-j Potential covalent binding modes of 4-OI (green) targeting Cys12 (a), Cys412 (c), Cys464 (e), Cys524 (g) and Cys716 (i) in the human IKKβ. The corresponding 2D ligand–protein interactions are displayed in (b), (d), (f), (g) and (j), respectively. Lipophilicity protein surface: lipophilic (cyan), hydrophilic (violet), neutral (white), α-helices (cyan), β-sheets (yellow), loops (cyan).

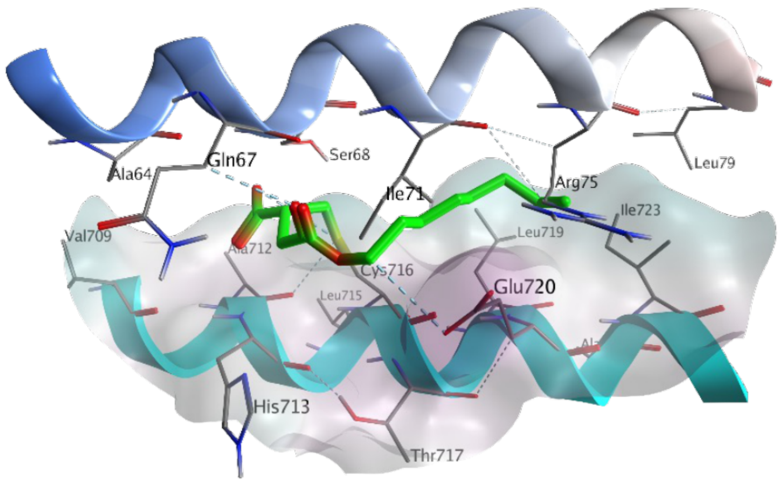

Figure S17. Modelling of 4-OI binding to Cys716 and interaction with NEMO
Overlay of Cys716-bound 4-OI (green) and the α-helix of NEMO (blue) showing steric interactions of the 4-OI octyl moiety with Gln67, Ser68, Ile71 and Arg75 of NEMO. Lipophilicity protein surface: lipophilic (cyan), hydrophilic (violet), neutral (white), α-helices (cyan), β-sheets (yellow), loops (cyan).

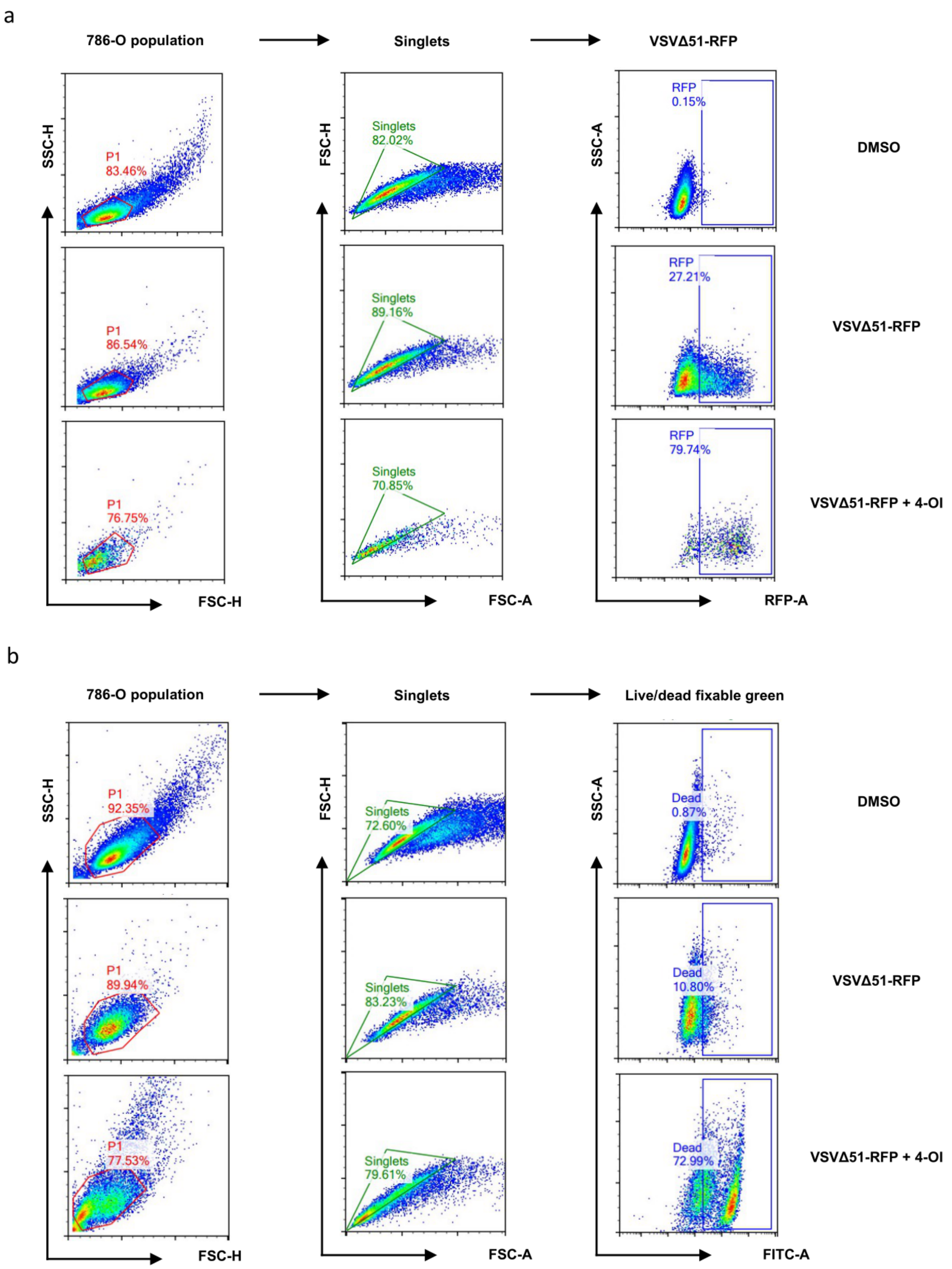

Figure S18. Gating strategies for flow cytometry

a Gating strategy for flow cytometric assessment of viral infection using RFP-expressing virus strain. Fluorescent flow cytometry data scale (RFP-A) is shown in logarithmic flavor ranging from 10^1-10^7

b Gating strategy for flow cytometric assessment of cellular viability. Fluorescent flow cytometry data scale (FITC-A) is shown in logarithmic flavor ranging from 10^3-10^9

Figure S19. Unprocessed immunoblots.

Fig. 4b

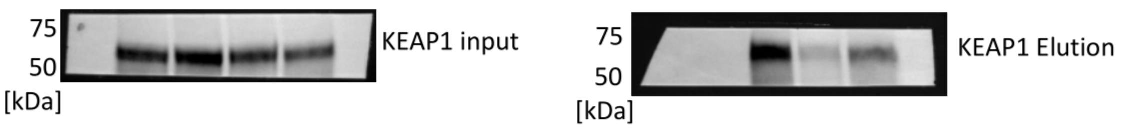

Fig. 4d

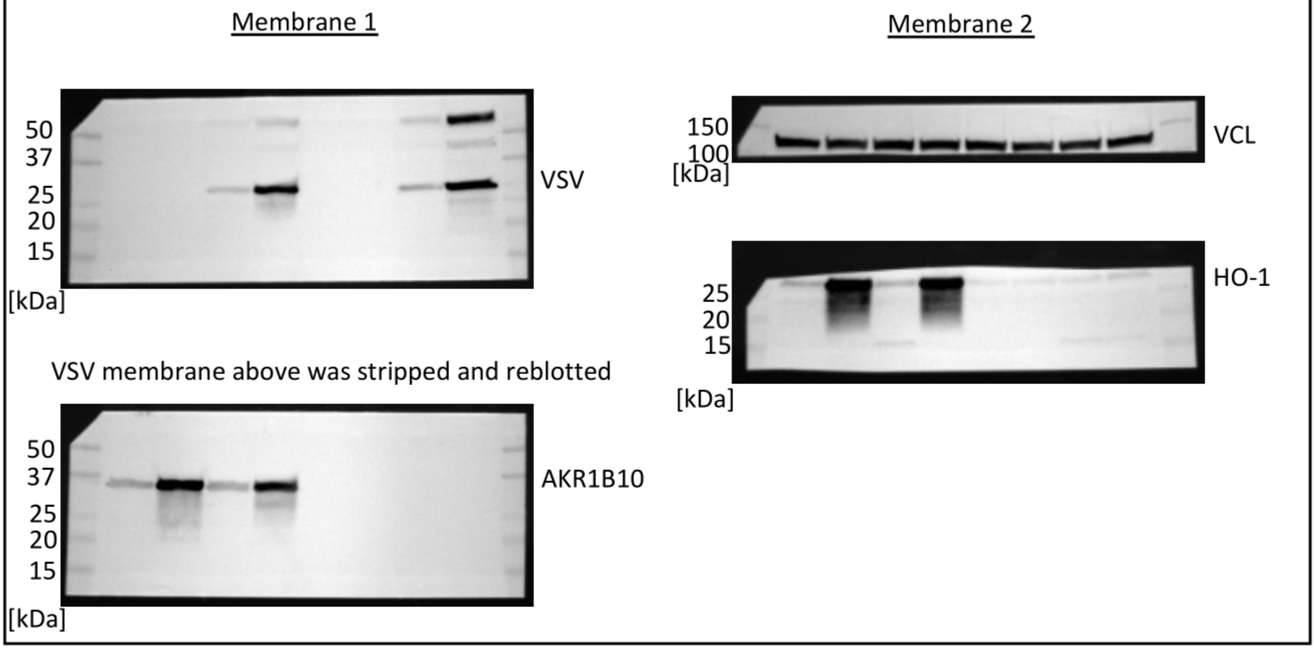

Fig. 4h

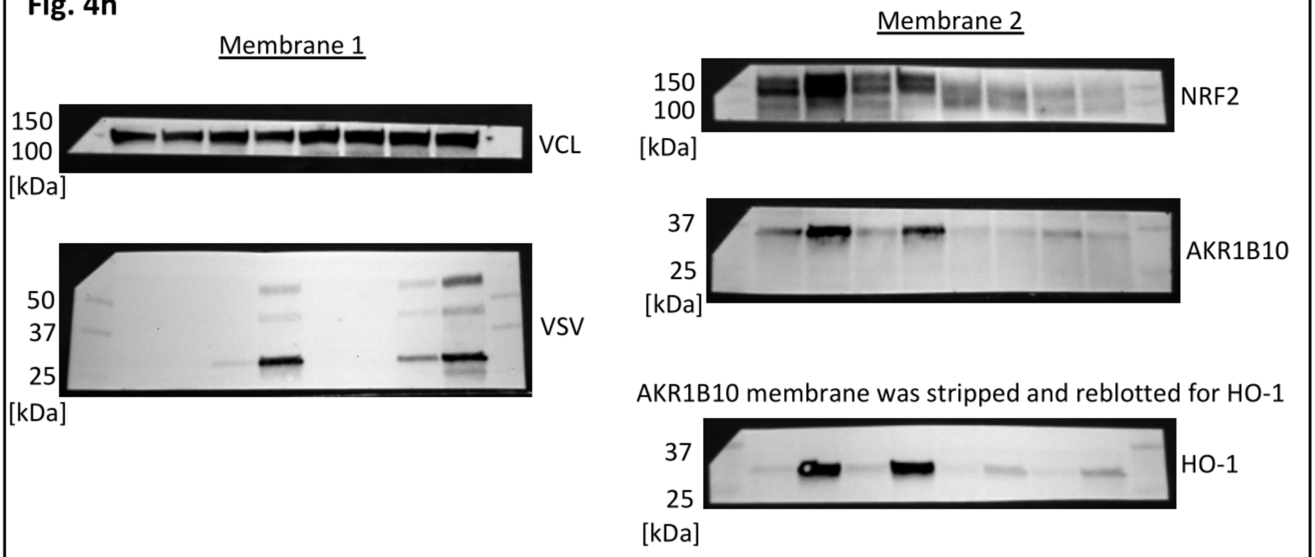

Fig. 4i

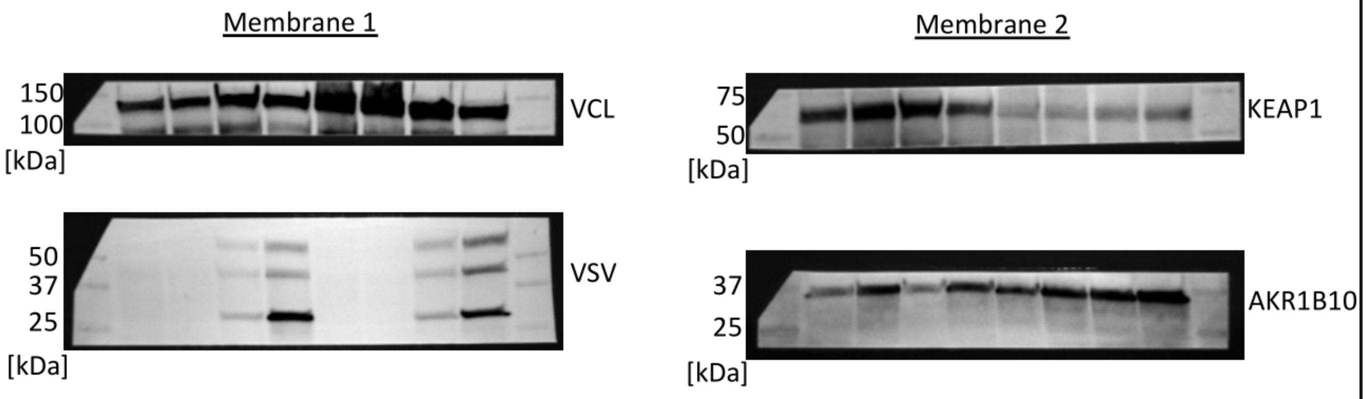
Membrane 1
150
100
[kDa]
VCL
50
37
25
[kDa]
VSV
Membrane 2
75
50
[kDa]
KEAP1
37
25
[kDa]
AKR1B10

Fig. 5e

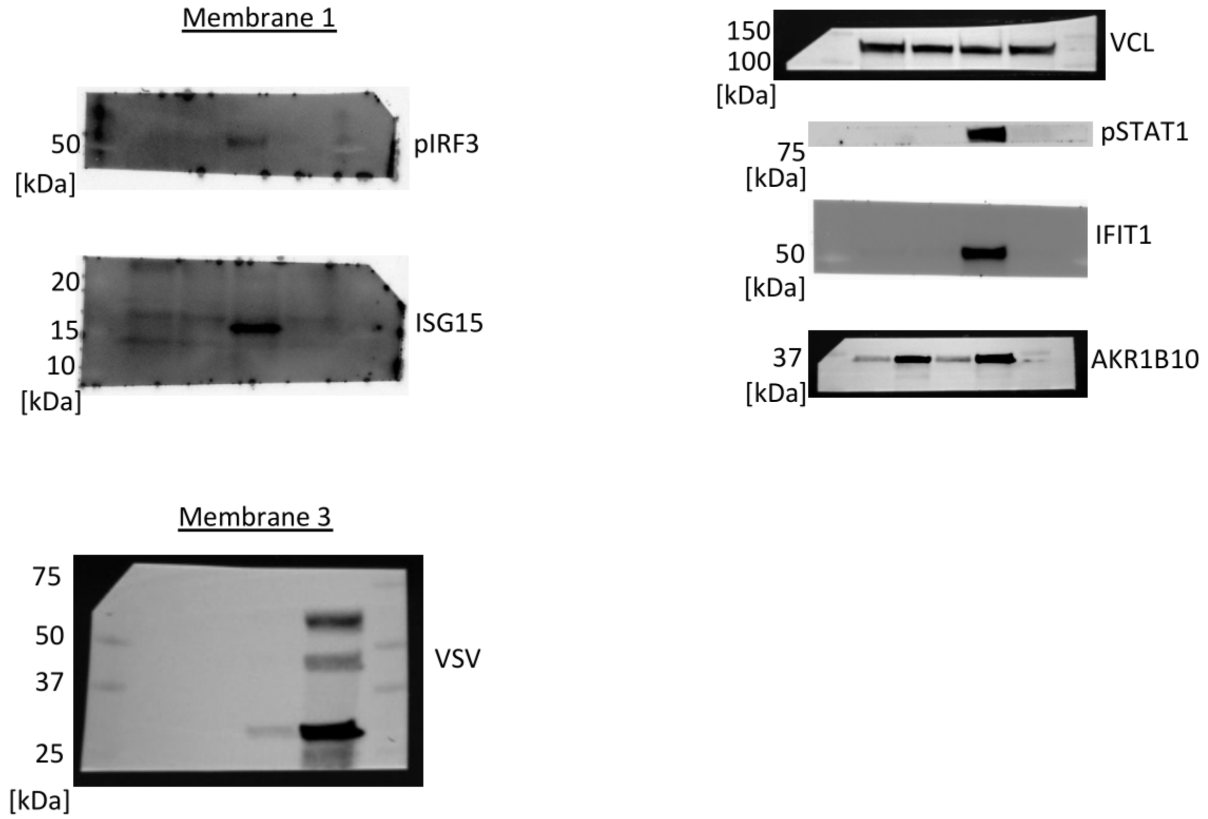
Membrane 1
50
[kDa]
pIRF3
20
15
10
[kDa]
ISG15
Membrane 3
75
50
37
25
[kDa]
VSV
Membrane 2
150
100
[kDa]
VCL
75
[kDa]
pSTAT1
50
[kDa]
IFIT1
37
[kDa]
AKR1B10

Fig. 5g

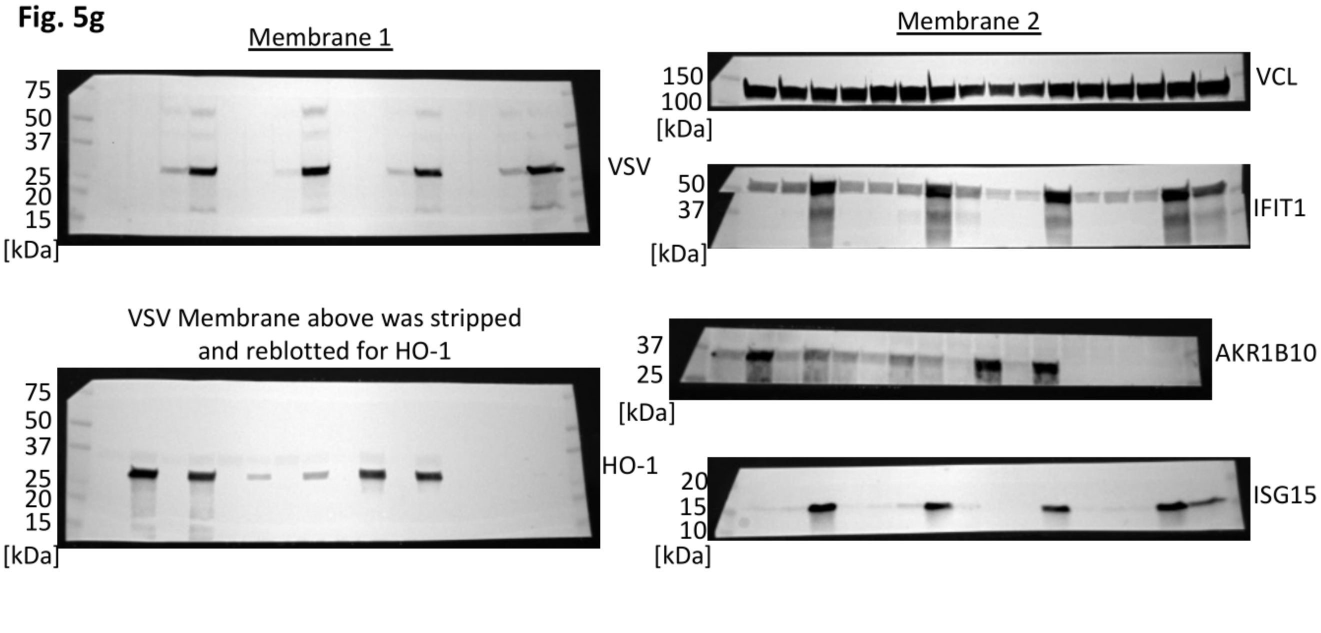
Membrane 1
75
50
37
25
20
15
[kDa]
VSV
VSV Membrane above was stripped and reblotted for HO-1
75
50
37
25
20
15
[kDa]
HO-1
Membrane 2
150
100
[kDa]
VCL
50
37
[kDa]
IFIT1
37
25
[kDa]
AKR1B10
20
15
10
[kDa]
ISG15

Fig. 5h

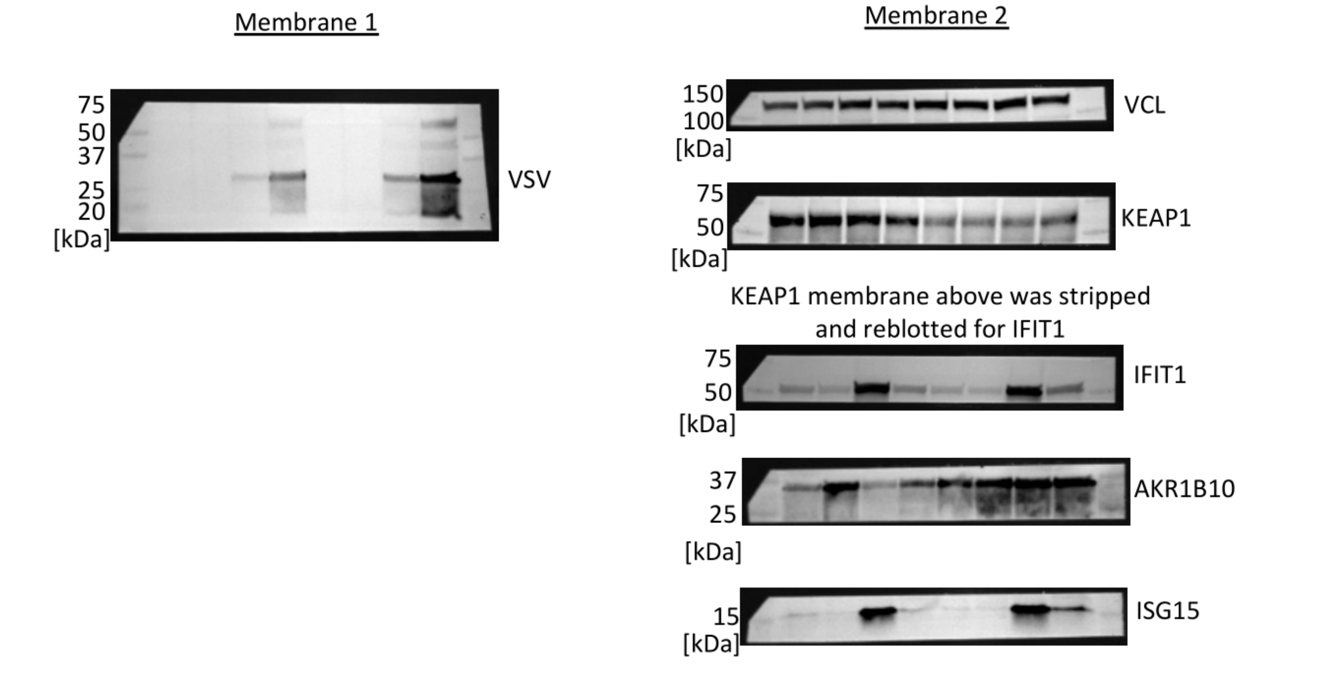
Membrane 1
75
50
37
25
20
[kDa]
VSV
Membrane 2
150
100
[kDa]
VCL
75
50
[kDa]
KEAP1
KEAP1 membrane above was stripped and reblotted for IFIT1
75
50
[kDa]
IFIT1
37
25
[kDa]
AKR1B10
15
[kDa]
ISG15

Fig. 5j

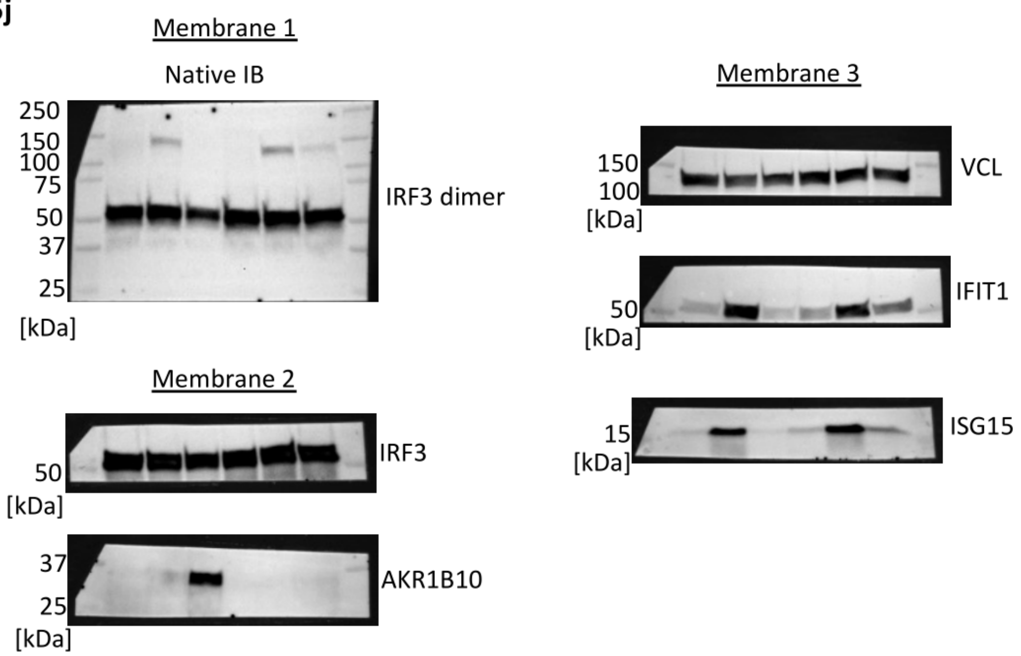
Membrane 1
Native IB
250
150
100
75
50
37
25
[kDa]
IRF3 dimer
Membrane 2
50
[kDa]
IRF3
37
25
[kDa]
AKR1B10
Membrane 3
150
100
[kDa]
VCL
50
[kDa]
IFIT1
15
[kDa]
ISG15

Fig. 7a

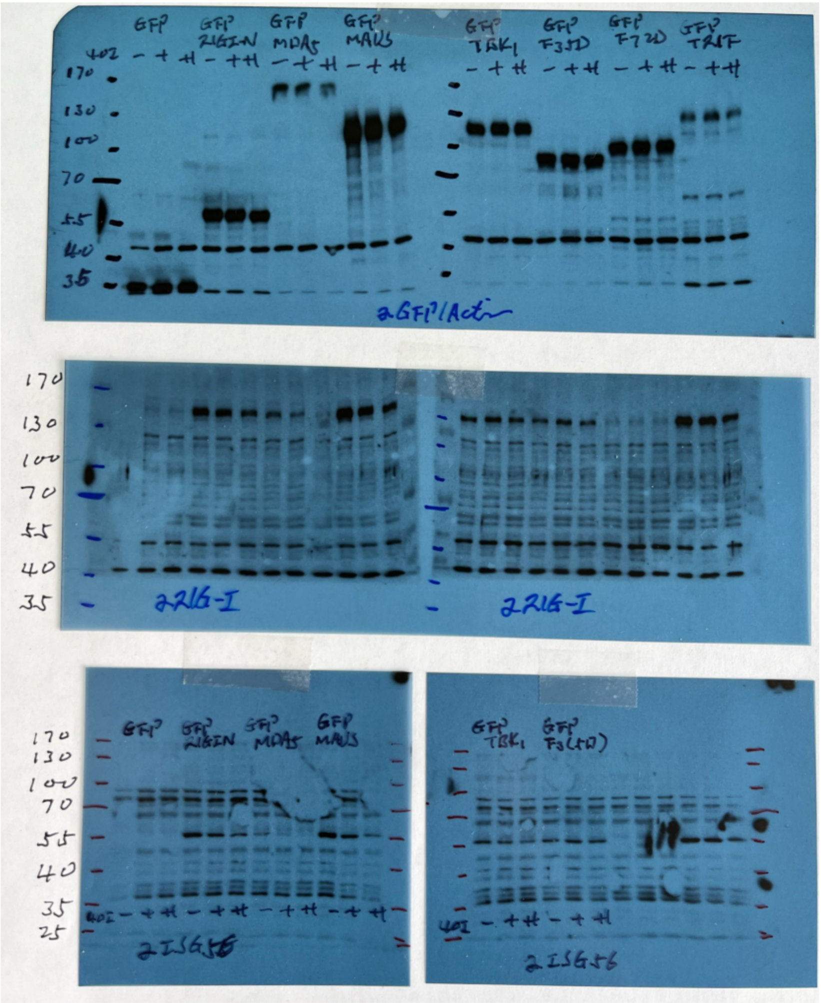
αGFP/Actin
αRIG-I
αRIG-I
αISG56
αISG56

Fig. 7d

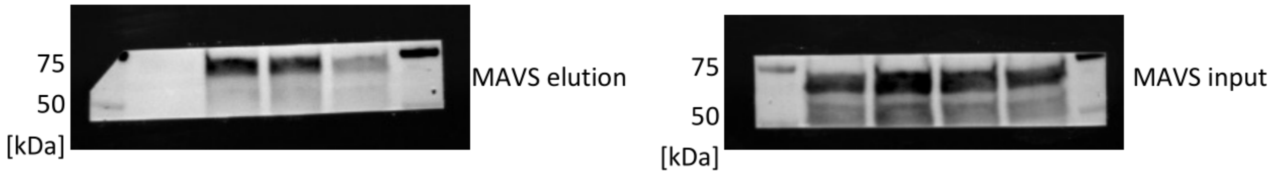
75
50
[kDa]
MAVS elution
75
50
[kDa]
MAVS input

Fig. 7e

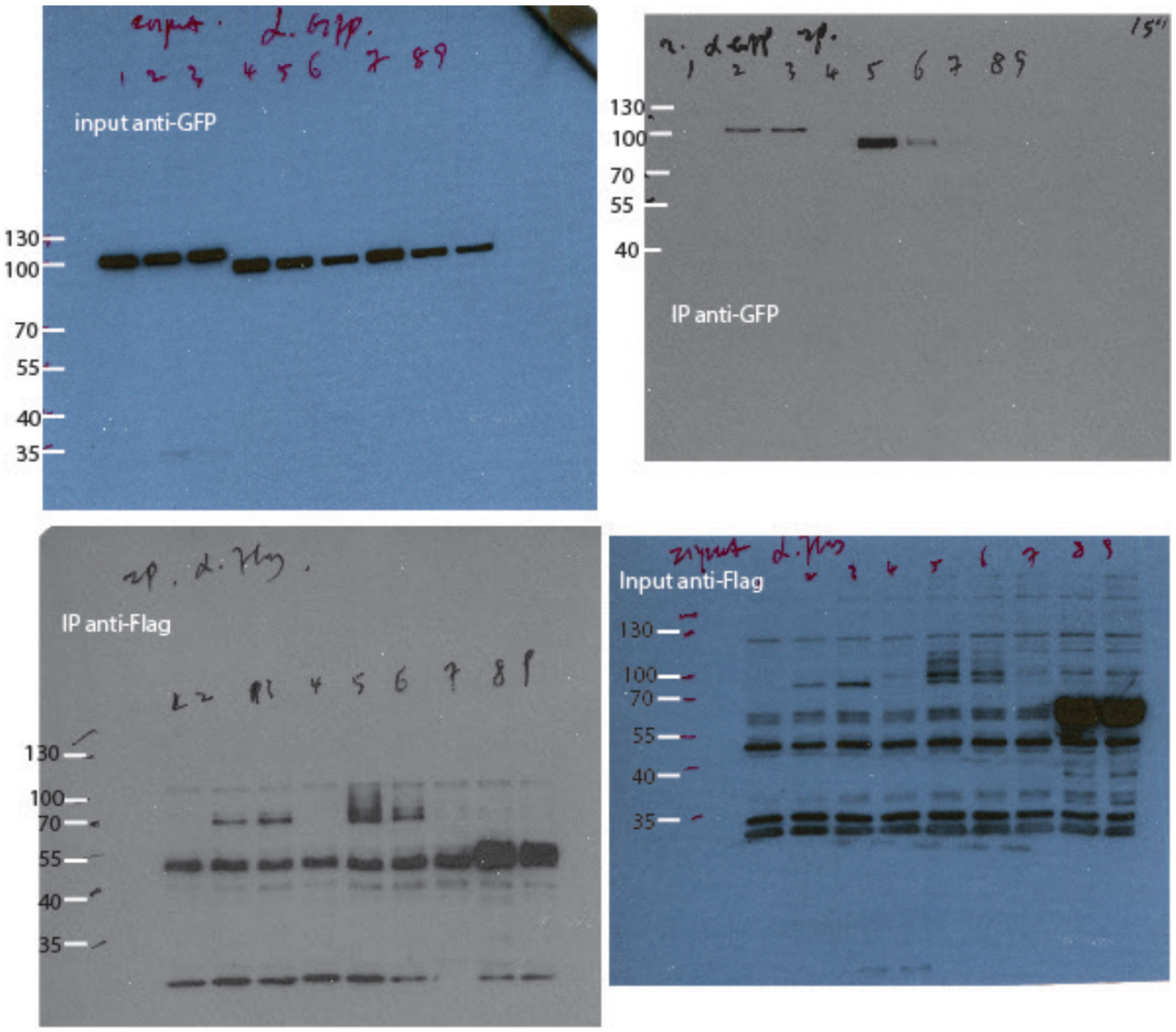
input anti-GFP
130
100
70
55
40
35
130
100
70
55
40
IP anti-GFP
IP anti-Flag
130
100
70
55
40
35
Input anti-Flag
130
100
70
55
40
35

Fig. 7f

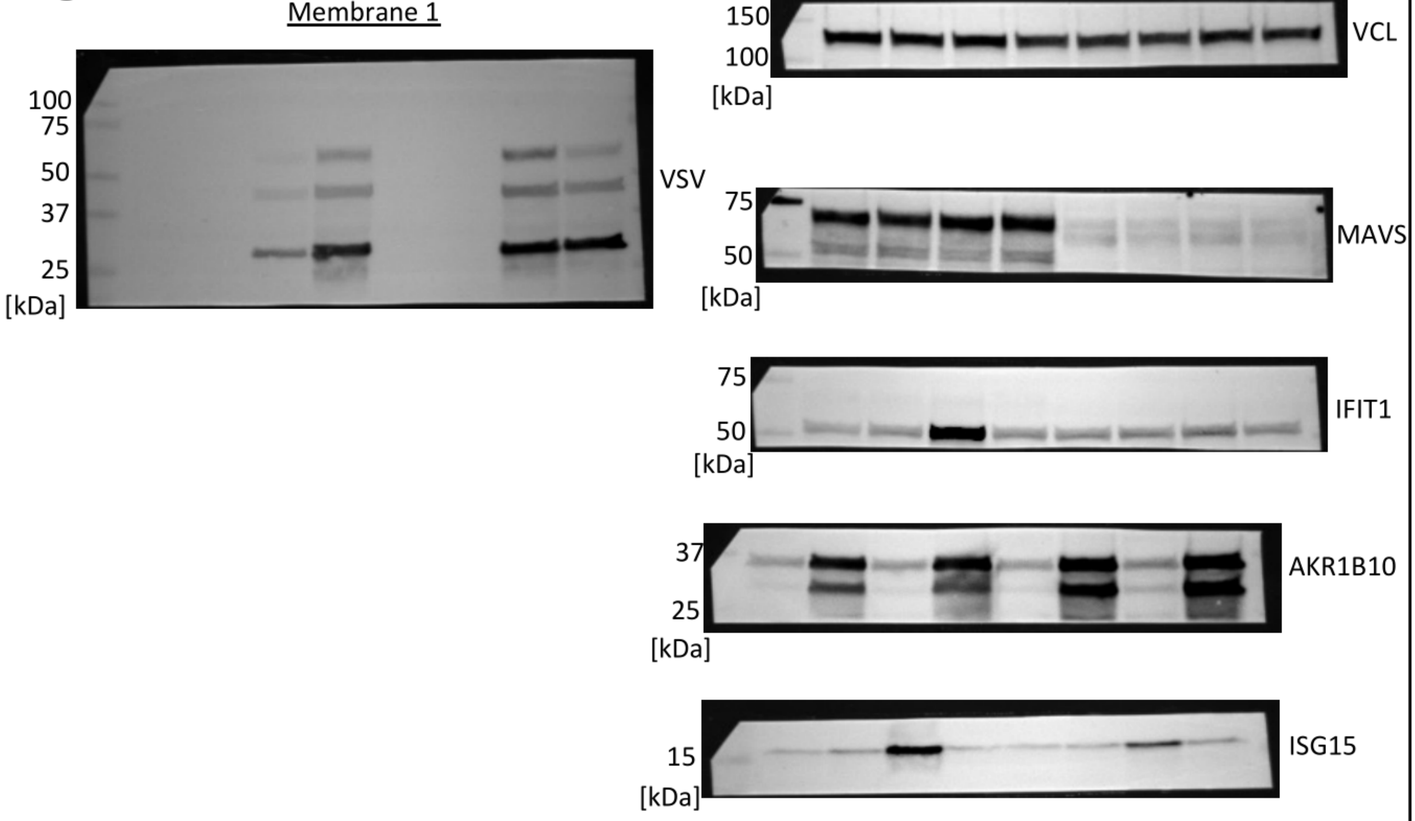
Membrane 1
100
75
50
37
25
[kDa]
VSV
Membrane 2
150
100
[kDa]
VCL
75
50
[kDa]
MAVS
75
50
[kDa]
IFIT1
37
25
[kDa]
AKR1B10
15
[kDa]
ISG15

Fig. 7h

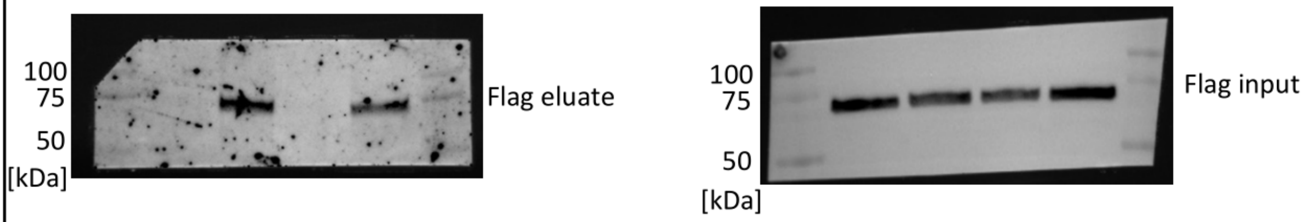
100
75
50
[kDa]
Flag eluate
100
75
50
[kDa]
Flag input

Fig. 7j

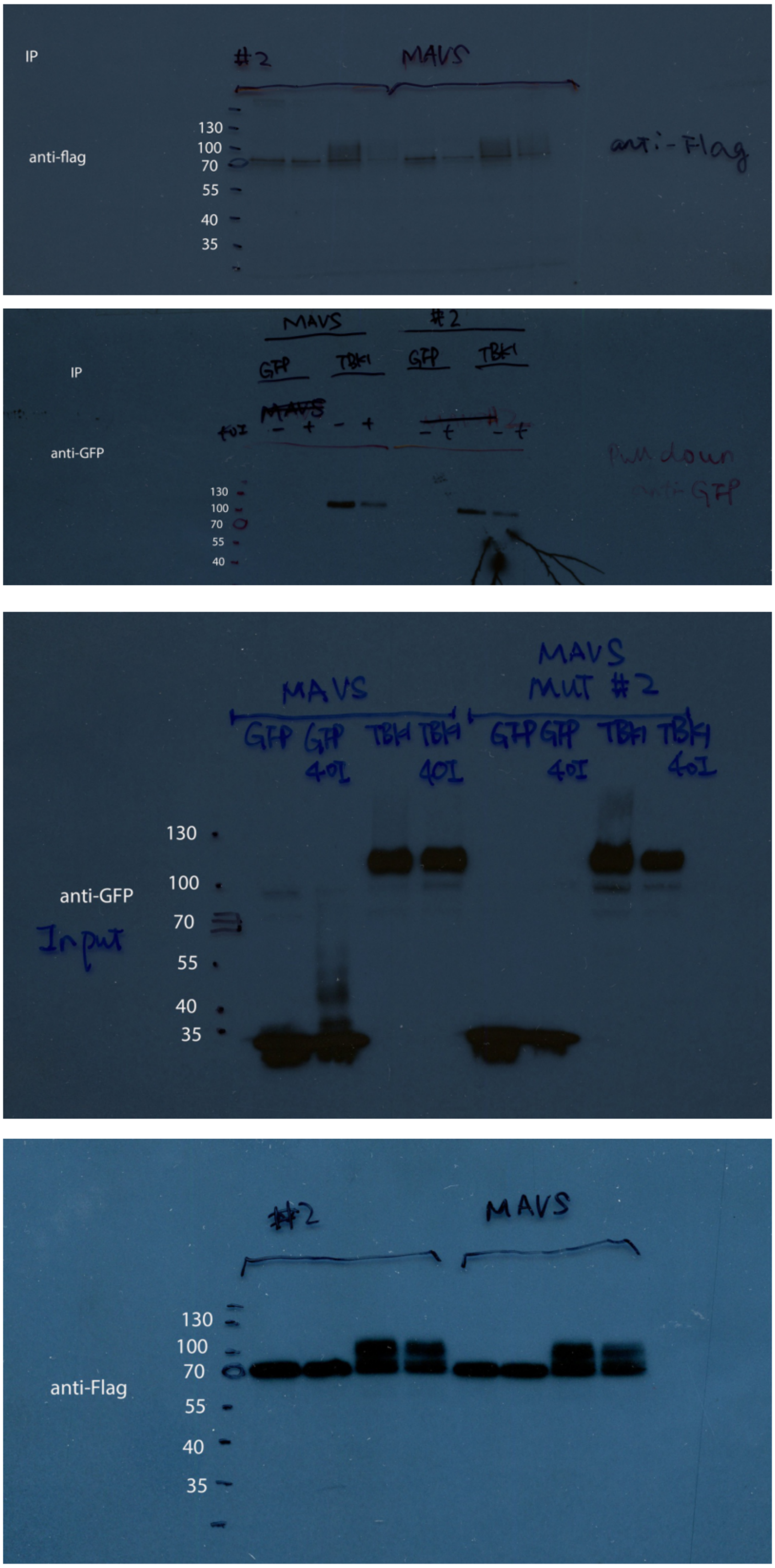
IP
anti-flag
130
100
70
55
40
35
IP
anti-GFP
130
100
70
55
40
anti-GFP
130
100
70
55
40
35
anti-Flag
130
100
70
55
40
35

Fig. 8f

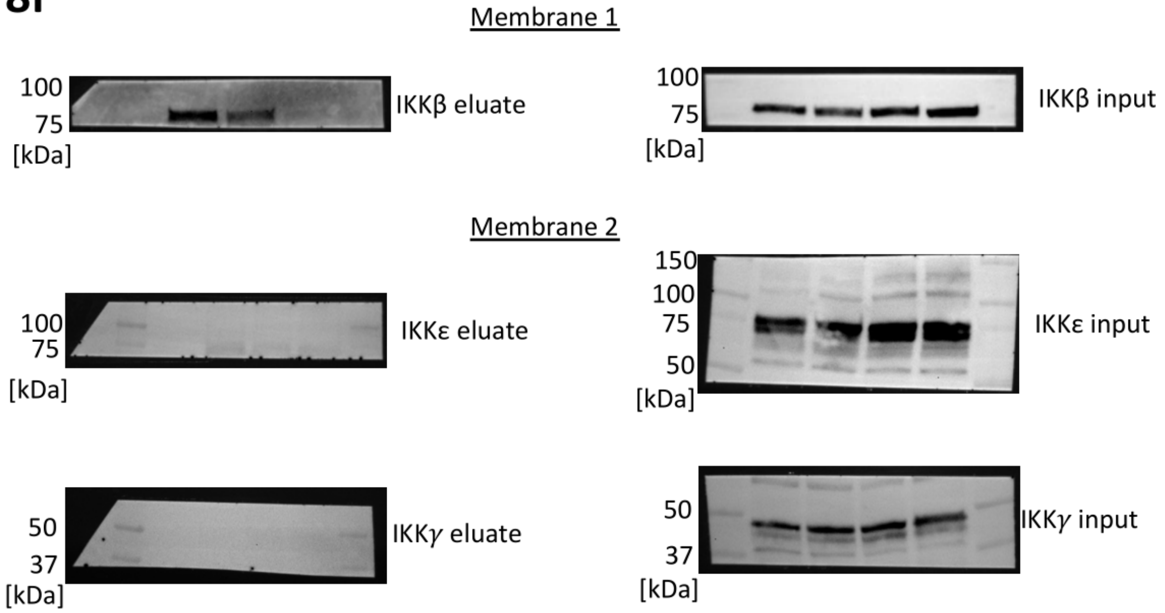
Membrane 1
100
75
[kDa]
IKKβ eluate
100
75
[kDa]
IKKβ input
Membrane 2
100
75
[kDa]
IKKε eluate
150
100
75
50
[kDa]
IKKε input
50
37
[kDa]
IKKγ eluate
50
37
[kDa]
IKKγ input

Fig. 8i

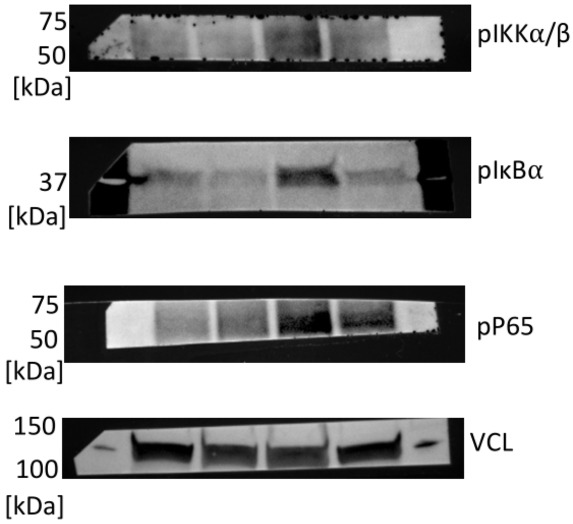
75
50
[kDa]
pIKKα/β
37
[kDa]
pIκBα
75
50
[kDa]
pP65
150
100
[kDa]
VCL

Fig. 8j

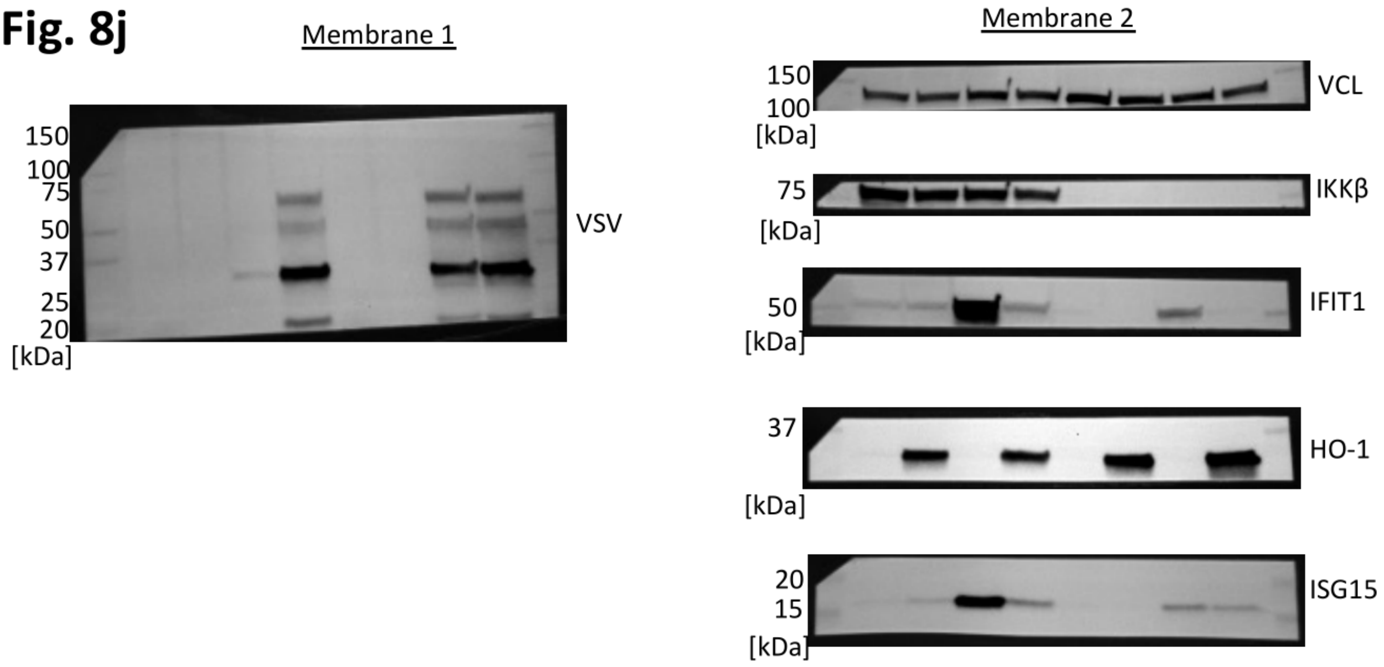

Fig. S8c

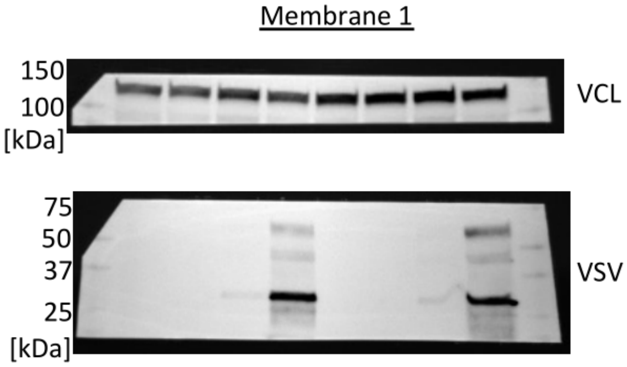

Fig. S9c

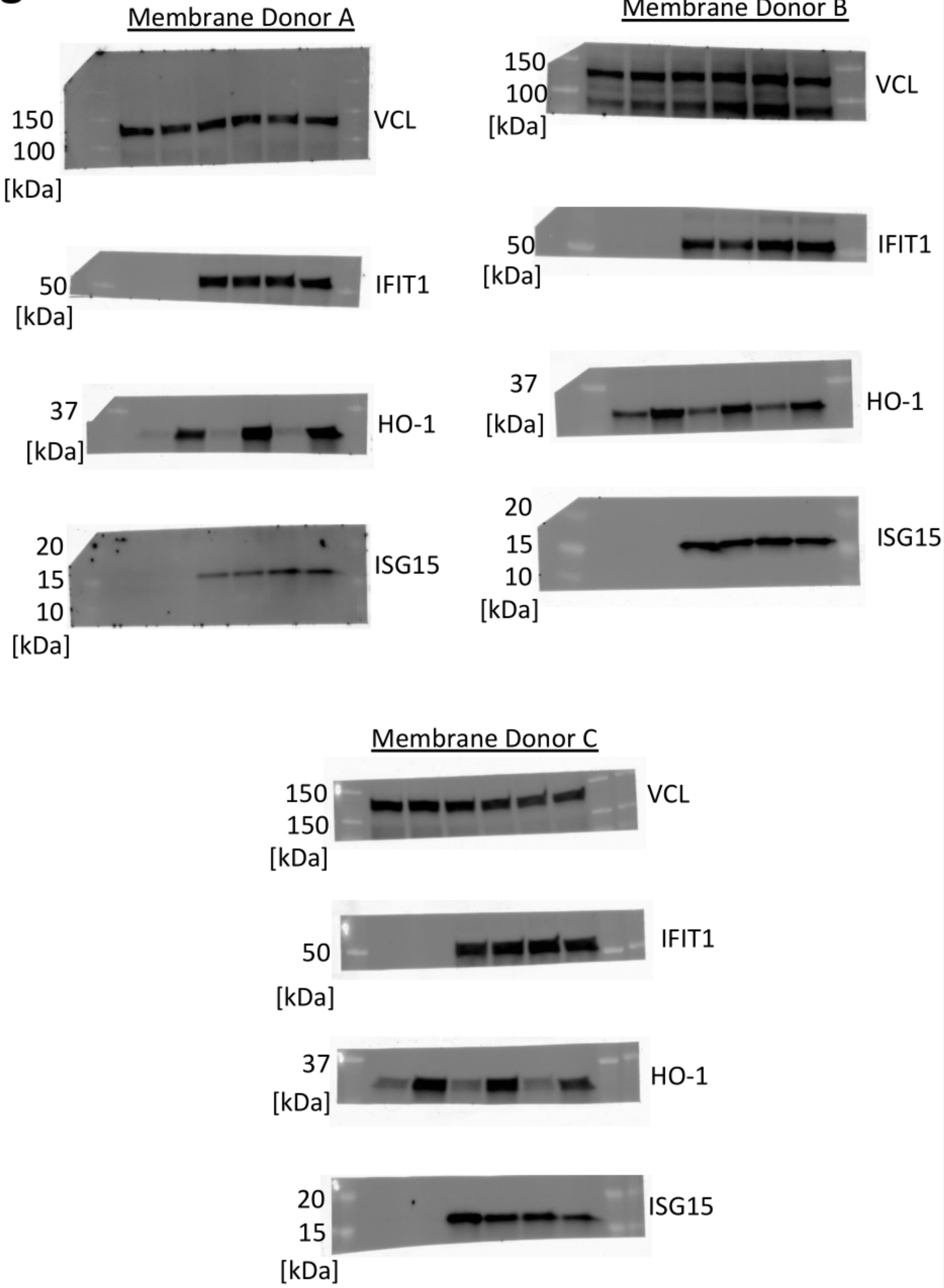
Membrane Donor A
150
100
[kDa]
VCL
50
[kDa]
IFIT1
37
[kDa]
HO-1
20
15
10
[kDa]
ISG15
Membrane Donor B
150
100
[kDa]
VCL
50
[kDa]
IFIT1
37
[kDa]
HO-1
20
15
10
[kDa]
ISG15
Membrane Donor C
150
150
[kDa]
VCL
50
[kDa]
IFIT1
37
[kDa]
HO-1
20
15
[kDa]
ISG15

Fig. S9d

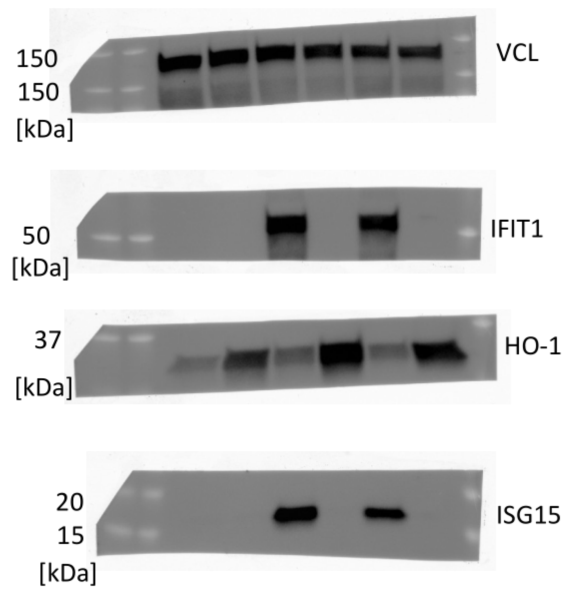

Fig. S12a

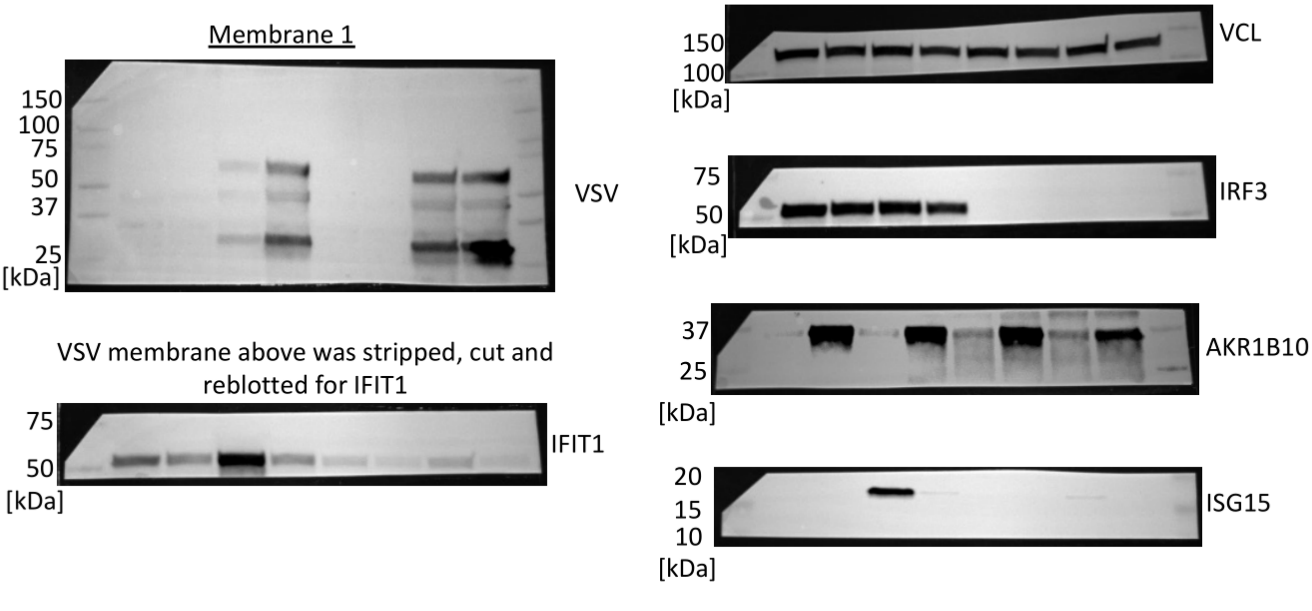

Fig. S12c

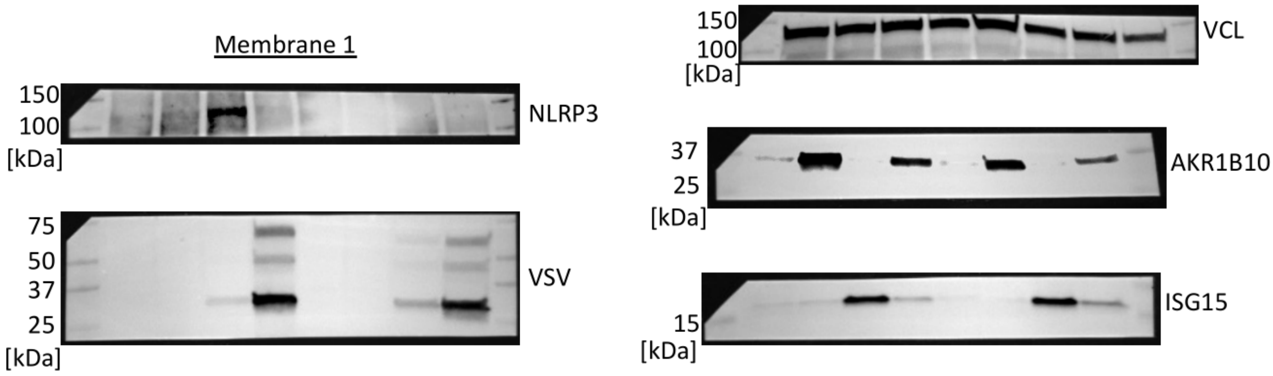

Fig. S13c

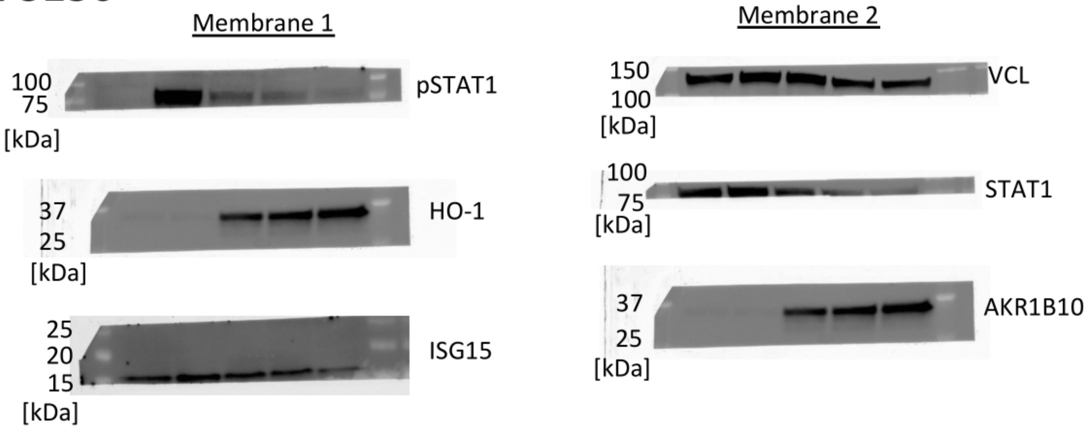

Fig. S13d

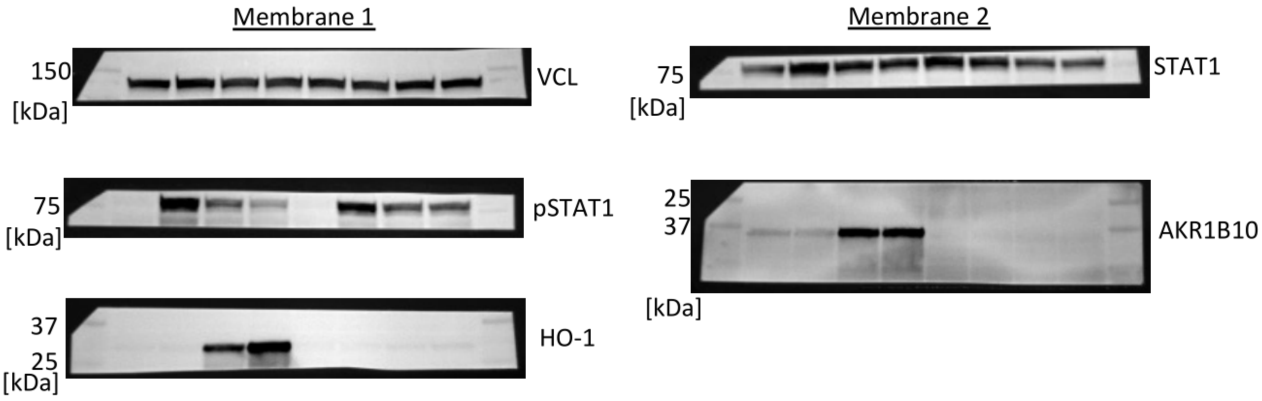

Fig. S13e

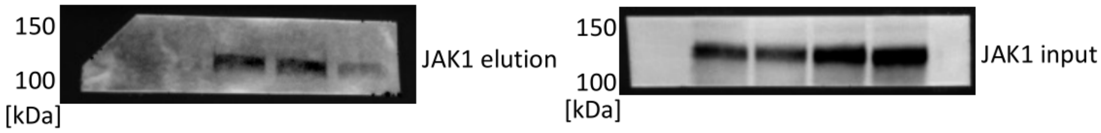

Fig. S13f

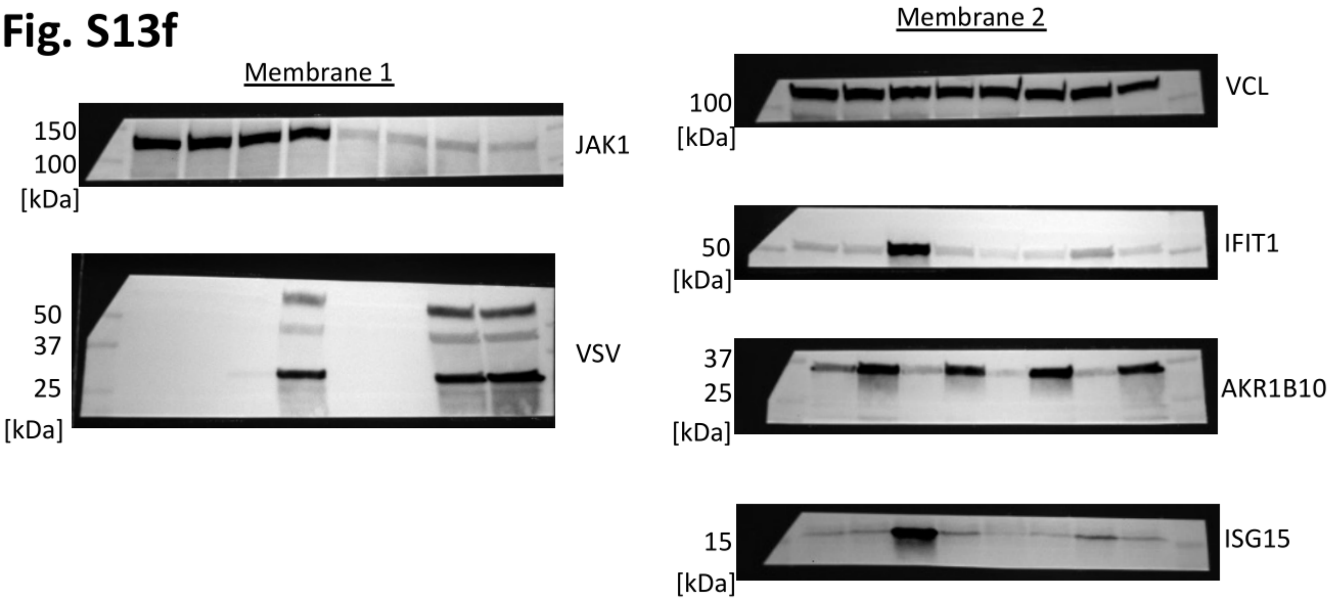

Fig. S15a

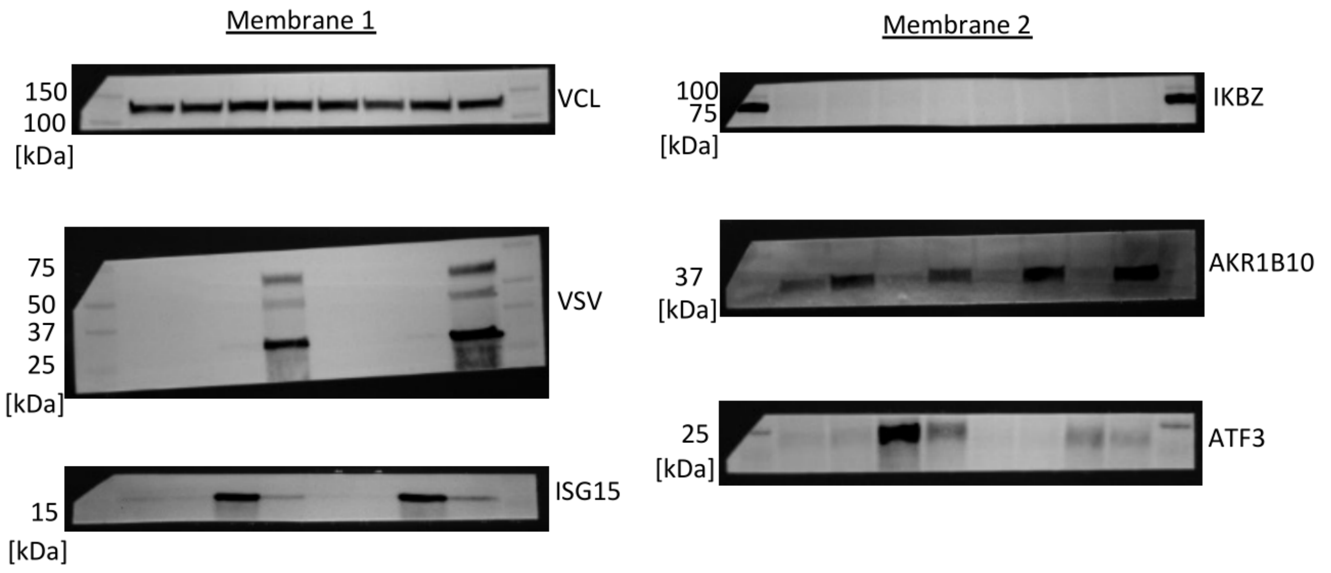

Fig. S15c

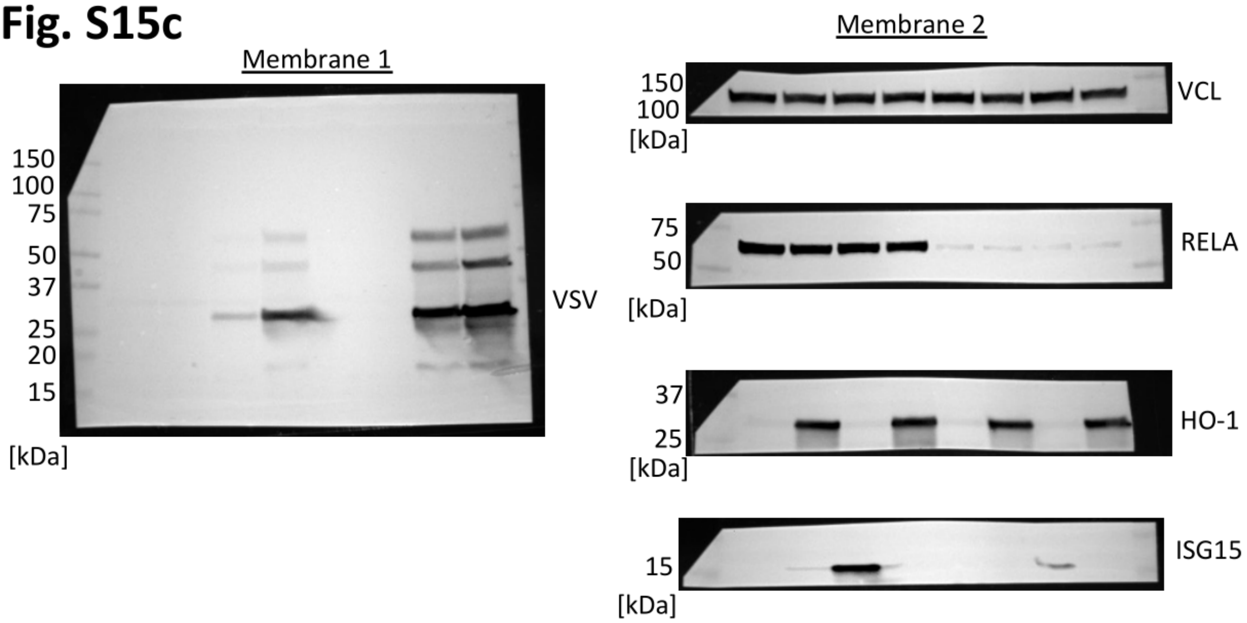

Fig. S15e

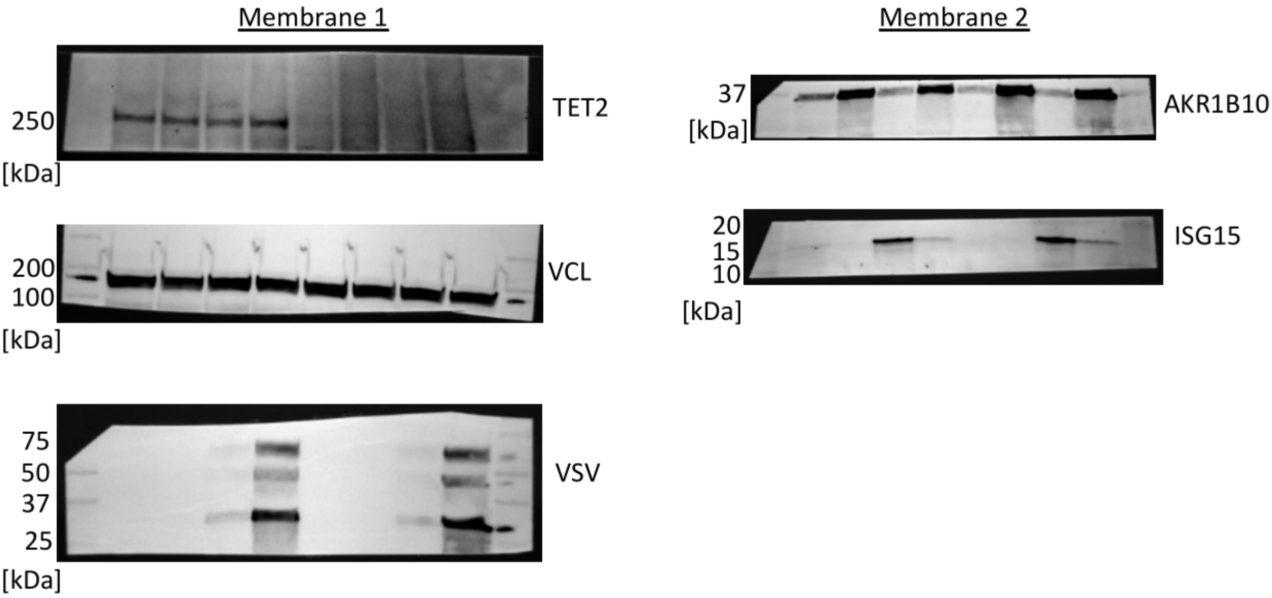

Supplementary Tables 1-6

No	Biobank ID (*)	Age	Sex	MMR status (**)	Tumor localization	UICC Classifi -cation	Prior Radiation Therapy	Prior Chemo-therapy	Normal organoids tested
P1	**O17**	78	male	MMRp	colon sigmoideum	IIIC	-	-	+
P2	**O11**	69	male	MMRp	colon sigmoideum	I	-	-	-
P3	**O23**	59	male	MMRp	rectum	IIIB	+	+	-
P4	**O29**	79	male	MMRp	colon descendens	IIIB	-	+	-
P5	**O06**	38	female	MMRp	colon sigmoideum	IIIA	-	-	+
P6	**O28**	68	male	MMRp	flexura hepatica	IVC	-	-	+
P7	**O09**	54	male	MMRp	rectum	IIIB	+	+	-
P8	**O13**	76	male	MMRp	colon descendens	IVB	+	+	-
P9	**O24**	55	male	MMRp	rectum	IVA	-	+	-
P10	**O07**	74	female	MMRd	caecum	IVB	-	-	-
P11	**O02**	78	male	MMRd	colon ascendens	IIA	-	-	-
P12	**O14**	46	male	MMRp	rectum	I	-	-	-

(*) part of the CRC organoid-stroma biobank (*under review*)

(**) organoids with >1000 somatic alterations detected by whole exome sequencing were defined as mismatch repair deficient (dMMR)

Table S1. Patient characteristics of colorectal organoids used in this study.

ID	Age (Years)	Sex	Tumor localization	Diagnosis
P1	78	Female	Left, occipital	Metastasis, malignant melanoma
P2	75	Male	Left, frontal	Newly diagnosed GBM, IDH-wt (WHO IV)
P3	60	Male	Right, parietal	Recurrent GBM, IDH-wt (WHO IV), radiochemotherapy

Table S2. Clinical data from all brain tumor patients.

Binding site	IKKβ Binding energy score (S) (kcal/mol)
Cys12	–6.8 to –7.6
Cys179	–7.0 to –8.1
Cys412	–8.2 to –10.0
Cys464	–7.7 to –9.0
Cys524	–6.5 to –7.1
Cys716	–5.1 to –5.7

Table S3. Binding energies of 4-OI to different cysteine-containing sites in IKKβ.

Fluorchrome	Target	Clone	Category number	Vendor	Dilution	Panel
**AF700**	CD8	53-6.7	56-0081-82	Thermofisher	1:100	T-cell
**AF700**	CD45.2	104	109822	Biolegend	1:400	Myeloid
**APC**	NKG2a	16A11	142807	Biolegend	1:100	T-cell
**APC**	XCR1	ZET	148206	Biolegend	1:200	Myeloid
**APC-Cy7**	PD1	29F.1A12	135224	Biolegend	1:50	T-cell
**APC-Cy7**	CD11c	N418	117324	Biolegend	1:100	Myeloid
**BV421**	CD62L	MEL-14	104436	Biolegend	1:100	T-cell
**BV421**	MHC-II	M5/114	562564	BD	1:400	Myeloid
**BV510**	Zombie Aqua		423101	Biolegend	1:800	All
**BV605**	CD69	H1.2F3	104530	Biolegend	1:100	T-cell
**BV605**	Ly6C	HK1.4	128036	Biolegend	1:500	Myeloid
**BV650**	NK1.1	PK136	564143	BD	1:100	T-cell
**BV650**	Siglec-H	440c	747672	BD	1:300	Myeloid
**BV711**	CD4	RM4-5	100549	Biolegend	1:200	T-cell
**BV711**	CD103	2E7	121435	Biolegend	1:200	Myeloid
**BV786**	CD44	IM7	103059	Biolegend	1:100	T-cell
**BV786**	Ly6G	1A8	127645	Biolegend	1:400	Myeloid
**FITC**	CD45.2	104	109806	Biolegend	1:200	T-cell
**FITC**	CD11b	ICRF44	562793	BD	1:500	Myeloid
**PE**	Foxp3	FJK-16s	12-5773-80	Thermofisher	1:200	T-cell
**PE**	CD86	GL-1	105007	Biolegend	1:400	Myeloid
**PECF594**	CD3	145-2C11	562286	BD	1:200	All
**PE-Cy7**	KLRG-1	2F1	25-5893-82	Invitrogen	1:200	T-cell
**PE-Cy7**	F4/80	BM8	123112	Biolegend	1:100	Myeloid
	CD16/CD32	2.4G2	553142	BD	1:250	All

Table S4. Listing of antibodies used in the *in vivo* immunophenotyping experiment.

Target	F/R	Sequence
VSV	Forward	CCTGATGACATTGAGTATACATCTCTT
VSV	Reverse	GGATCCTACTGCATAAGCGTACA
ISG15	Forward	GGAACGAAAGGGGCCACAGCA
ISG15	Reverse	CCTCCATGGGCCTTCCCTCGA
NFKB	Forward	CACCTAGCTGCCAAAGAAGG
NFKB	Reverse	GCAGGCTATTGCTCATCACA
CXCL9	Forward	TGGAGTTCGAGGAACCCTAGT
CXCL9	Reverse	AGGCAGGTTTGATCTCCGTT
Mzt2	Forward	TCGGTGCCCATATCTCTGTC
Mzt2	Reverse	CTGCTTCGGGAGTTGCTTTT
Ptp4a	Forward	AGCCCCTGTGGAGATCTCTT
Ptp4a	Reverse	AGCATCACAAACTCGAACCA
MX1	Forward	CTGGAAGCACTGTCTGGAGT
MX1	Reverse	GGCCTCTTCCACCTCTGAAG
HMOX1	Forward	GGTCAGGTGTCCAGAGAAGG
HMOX1	Reverse	ATGATTTCCTGCCAGTGAGG
IL6	Forward	AACGATGATGCACTTGCAGA
IL6	Reverse	CTCTGAAGGACTCTGGCTTTG
IFIT1	Forward	CTGGACAAGGTGGAGAAGGT
IFIT1	Reverse	AGGGTTTTCTGGCTCCACTT
IL1b	Forward	GACCTTCCAGGATGAGGACA
IL1b	Reverse	AGCTCATATGGGTCCGACAG
TNF	Forward	GAACTGGCAGAAGAGGCACT
TNF	Reverse	AGGGTCTGGGCCATAGAACT
IFNbeta	Forward	AGAAAGGACGAACATTCGGAAA
IFNbeta	Reverse	CCGTCATCTCCATAGGGATCTT
Viperin	Forward	TTGGGCAAGCTTGTGAGATTC
Viperin	Reverse	TGAACCATCTCTCCTGGATAAGG

Table S5. Listing of the primers used for the *in vivo* intratumoral qPCR experiments.

Name	Sequence
hsMx1-F	GTTTCCGAAGTGGACATCGCA
hsMx1-R	CTGCACAGGTTGTTCTCAGC
hsISG15-F	CGCAGATCACCCAGAAGATCG
hsISG15-R	TTCGTCGCATTTGTCCACCA
hsIFNB1-F	ATGACCAACAAGTGTCTCCTCC
hsIFNB1-R	GGAATCCAAGCAAGTTGTAGCTC
hsIFIT1-F	TTGATGACGATGAAATGCCTGA
hsIFIT1-R	CAGGTCACCAGACTCCTCAC
hsTNF-F	CCTCTCTCTAATCAGCCCTCTG
hsTNF-R	GAGGACCTGGGAGTAGATGAG
hsIL6-F	ACTCACCTCTTCAGAACGAATTG
hsIL6-R	CCATCTTTGGAAGGTTCAGGTTG
hsIL1b-F	ATGATGGCTTATTACAGTGGCAA
hsIL1b-R	GTCGGAGATTCGTAGCTGGA
hsHPRT1-F	CCTGGCGTCGTGATTAGTGAT
hsHPRT1-R	AGACGTTCAGTCCTGTCCATAA

Table S6. Listing of the primers used for qPCR on measles, reovirus and vaccinia virus-infected cells.

Supplementary Methods

Copies of NMR spectra

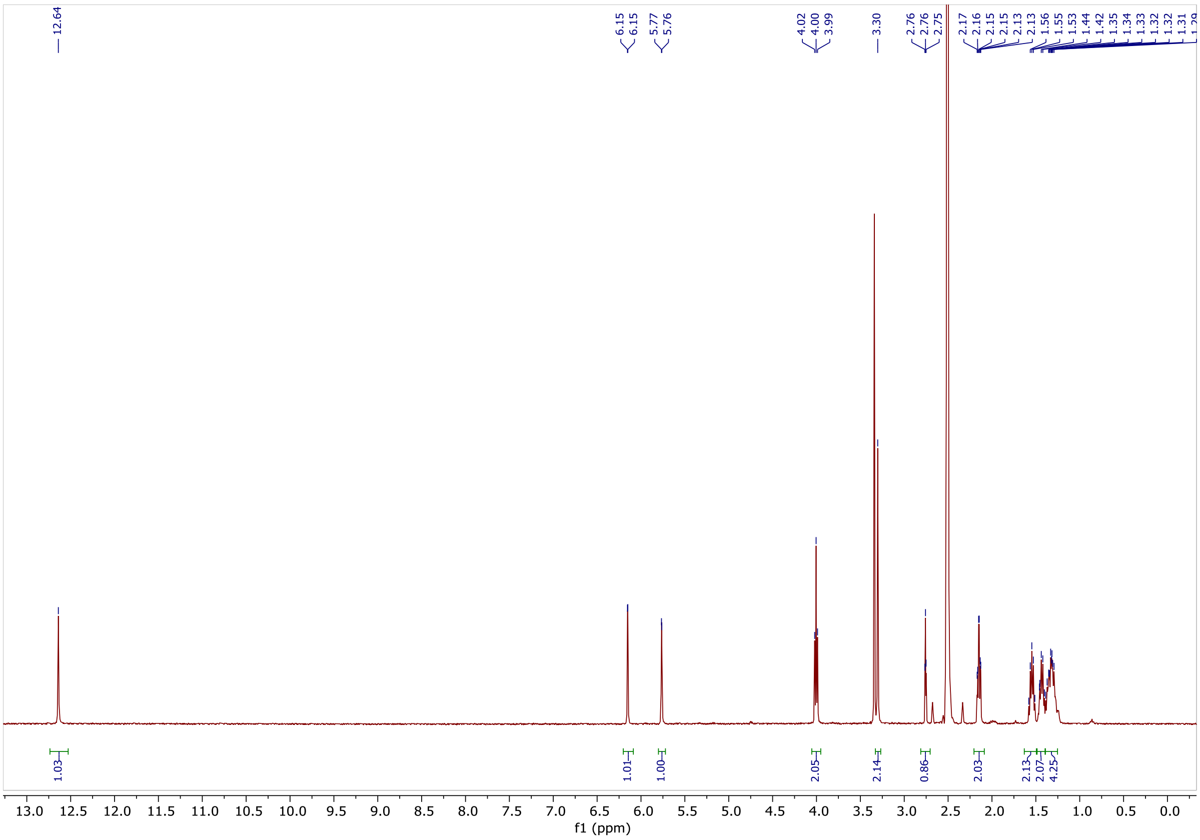

Figure: ^{1}H NMR spectrum (400 MHz, DMSO-d_6) of **4-OI-alk**

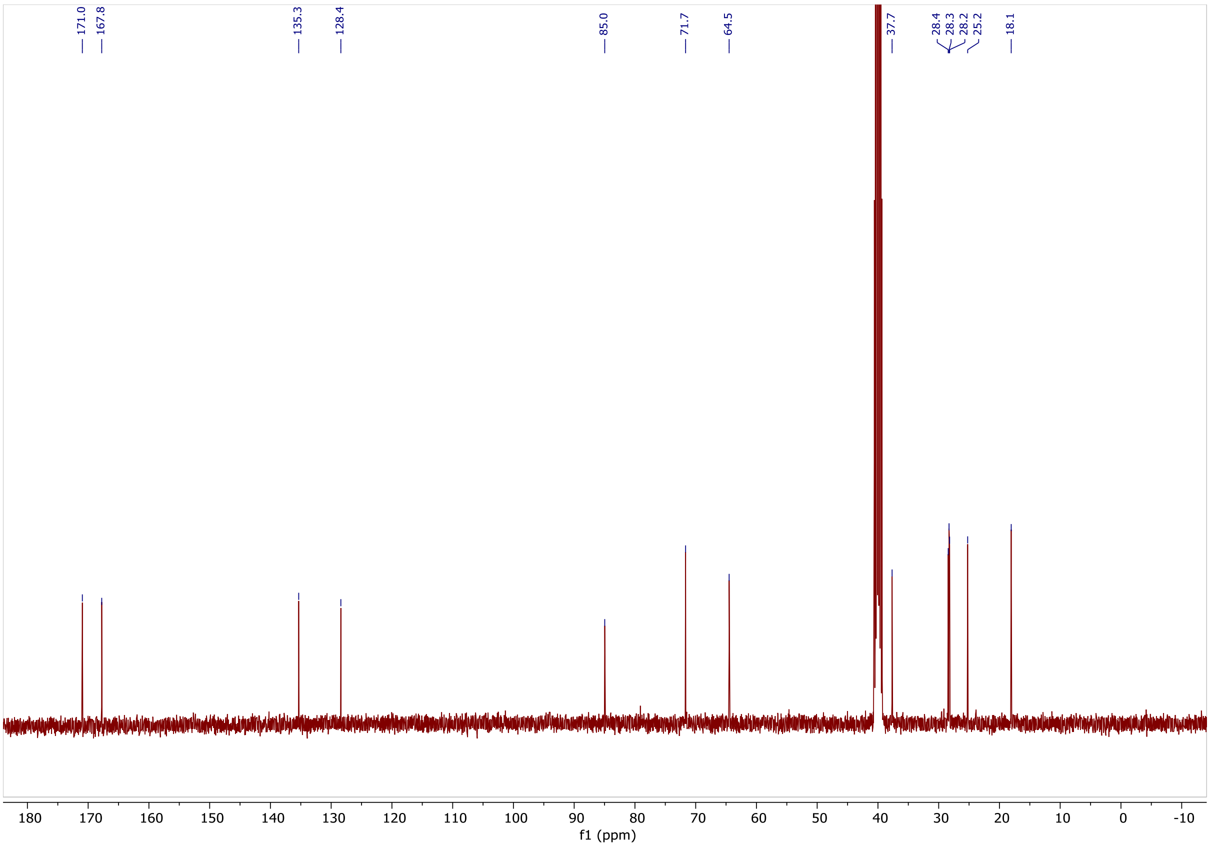

Figure: ^{13}C NMR spectrum (101 MHz, DMSO-d_6) of **4-OI-alk**

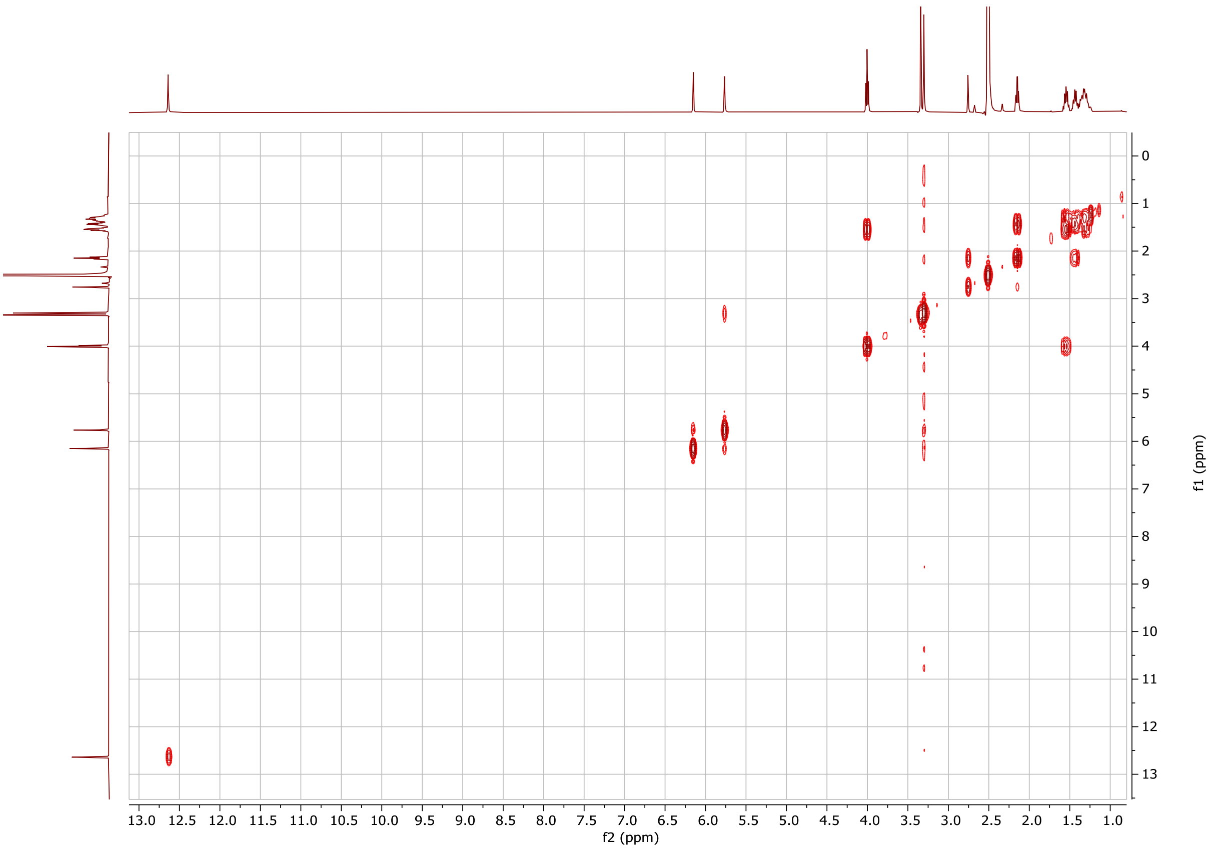

Figure: COSY spectrum (400 MHz, DMSO-d_6) of **4-OI-alk**

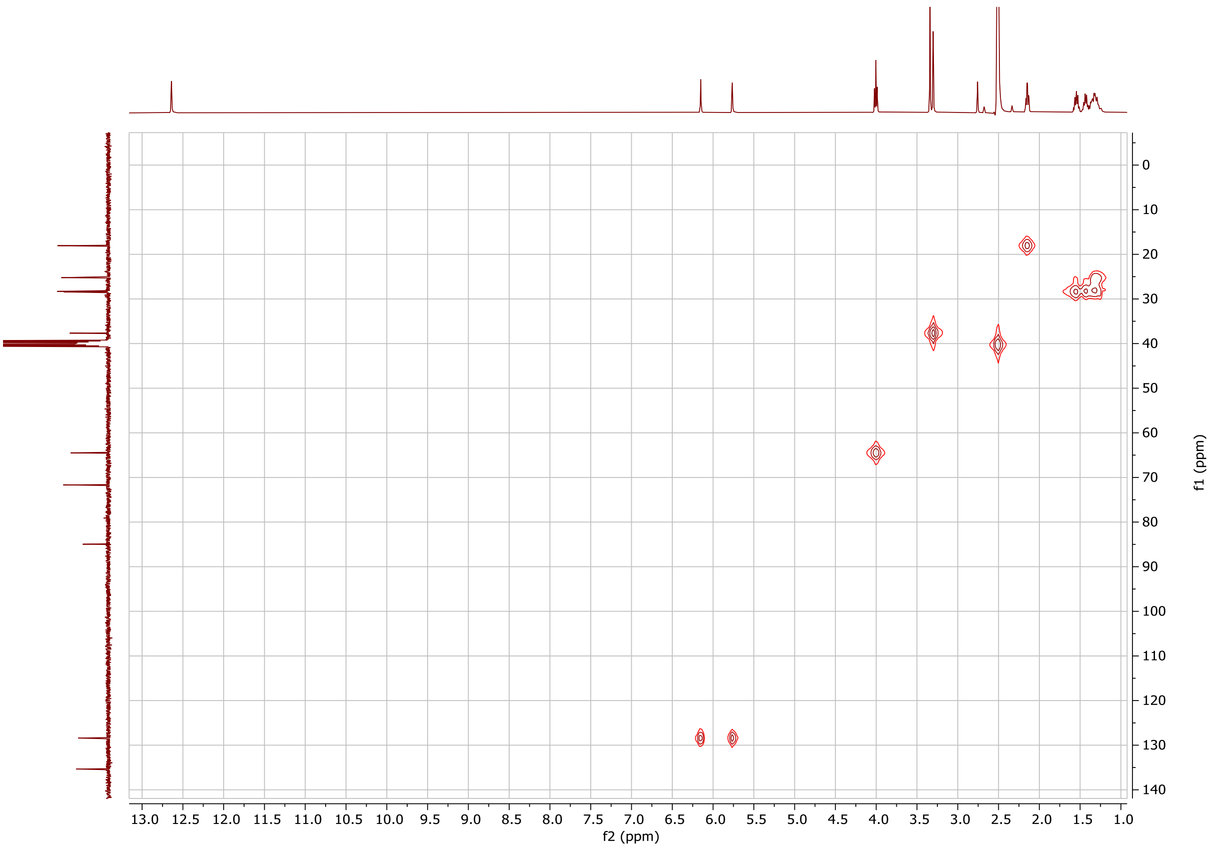

Figure: HSQC NMR spectrum (400/101 MHz, DMSO-d_6) of **4-OI-alk**

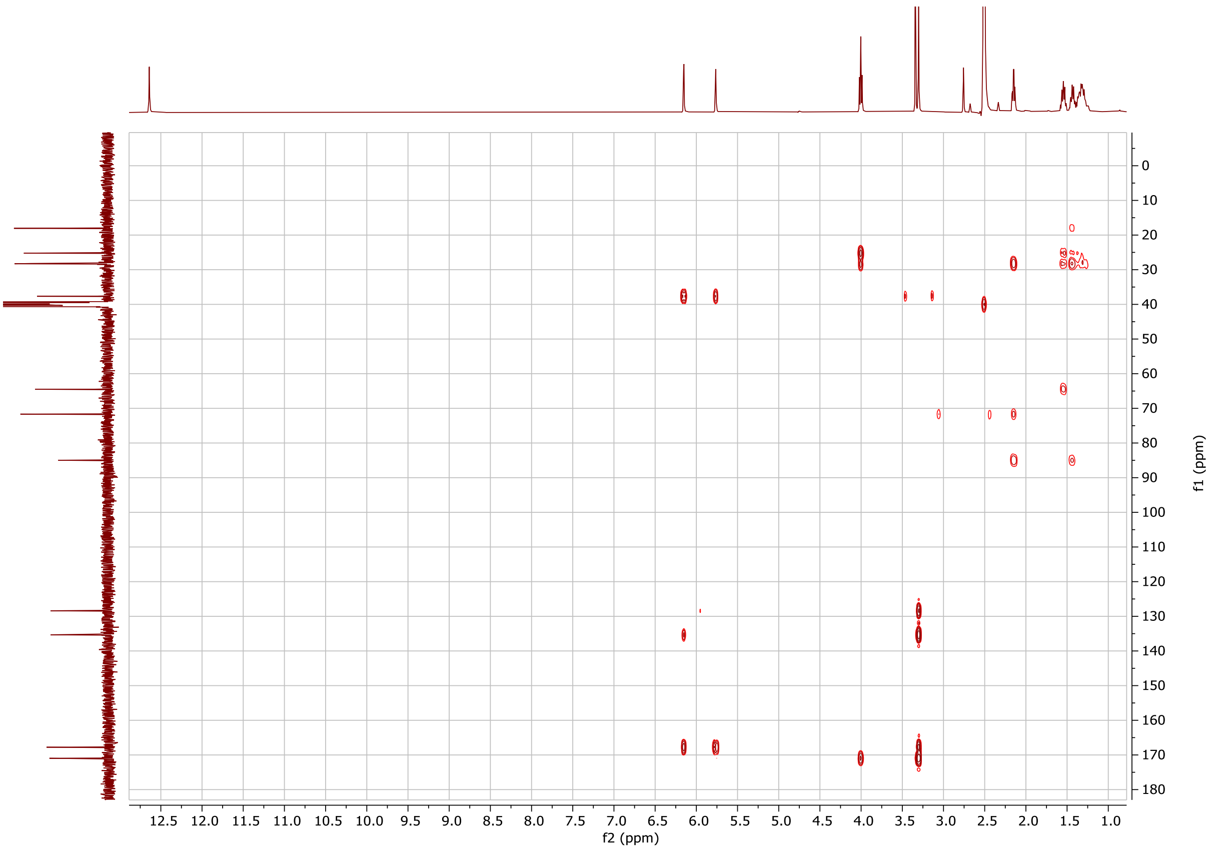

Figure : HMBC NMR spectrum (400/101 MHz, DMSO-d_6) of **4-OI-alk**

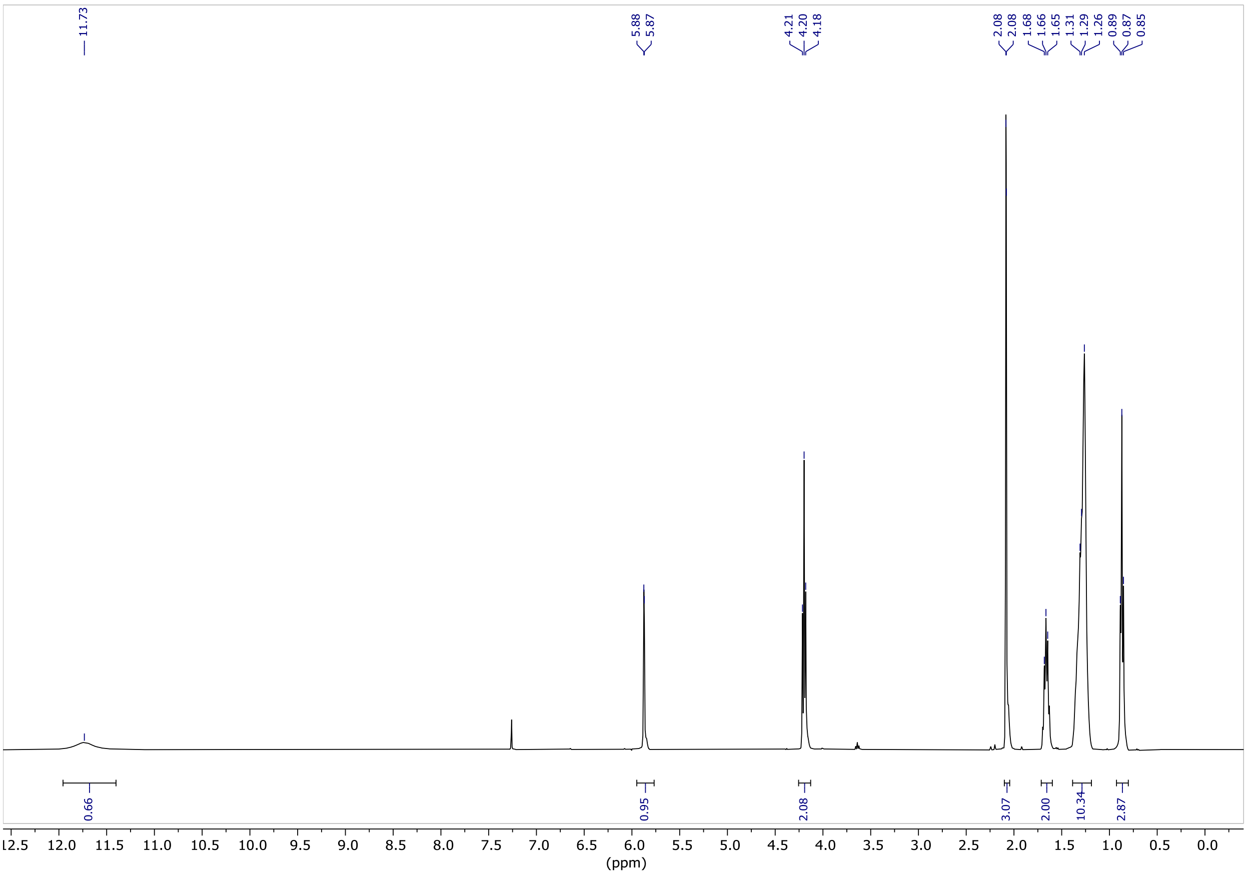

Figure: ^{1}H NMR spectrum (400 MHz, $CDCl_3$) of **1-OC (HS)**

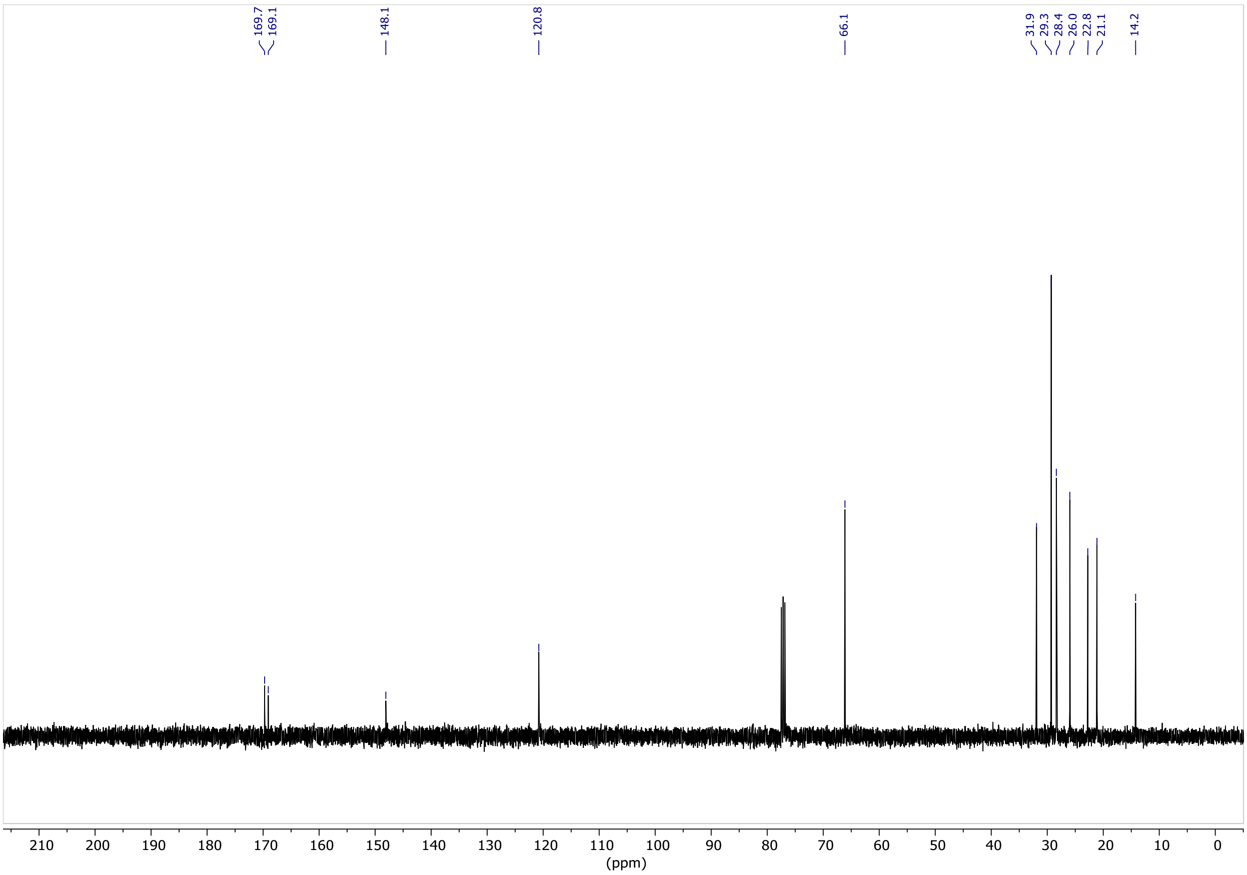

Figure: ^{13}C NMR spectrum (101 MHz, $CDCl_3$) of **1-OC (HS)**

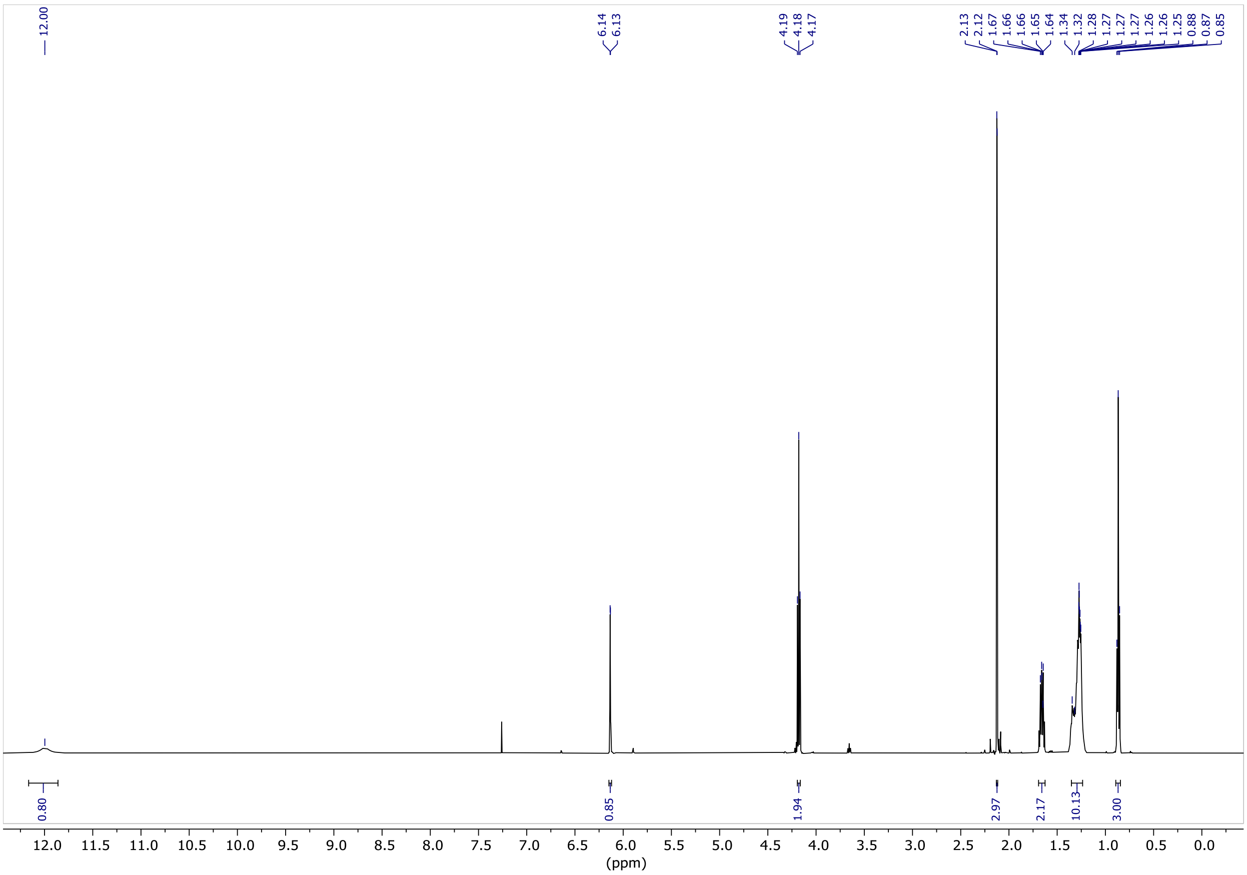

Figure: ^{1}H NMR spectrum (400 MHz, $CDCl_3$) of **4-OC (LS)**

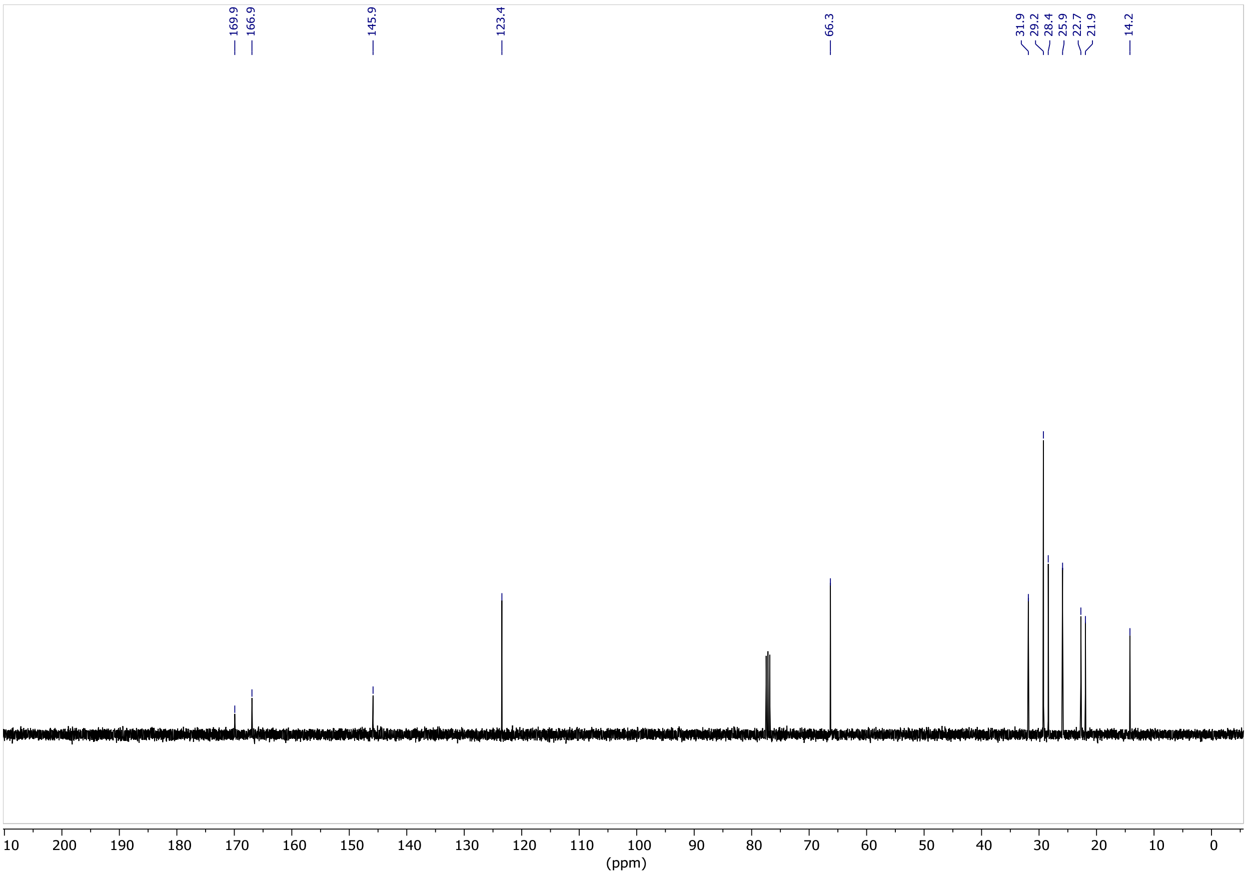

Figure: ^{13}C NMR spectrum (101 MHz, $CDCl_3$) of **4-OC (HS)**
