# Supplementary material for: Octyl itaconate enhances VSVΔ51 oncolytic virotherapy by multitarget inhibition of antiviral and inflammatory pathways
Source: Nat Commun. 2024 May 15;15:4096. doi: 10.1038/s41467-024-48422-x (PMC11096414; doi:10.1038/s41467-024-48422-x)
Supplement: Supplementary file 3 — Reporting Summary [file 41467_2024_48422_MOESM3_ESM.pdf]

nature portfolio

Corresponding author(s): David OLAGNIER

Last updated by author(s): Mar 27, 2024

Reporting Summary

Nature Portfolio wishes to improve the reproducibility of the work that we publish. This form provides structure for consistency and transparency in reporting. For further information on Nature Portfolio policies, see our Editorial Policies and the Editorial Policy Checklist.

Statistics

For all statistical analyses, confirm that the following items are present in the figure legend, table legend, main text, or Methods section.

n/a	Confirmed	
☐	☒	The exact sample size (*n*) for each experimental group/condition, given as a discrete number and unit of measurement
☐	☒	A statement on whether measurements were taken from distinct samples or whether the same sample was measured repeatedly
☐	☒	The statistical test(s) used AND whether they are one- or two-sided<br>*Only common tests should be described solely by name; describe more complex techniques in the Methods section.*
☐	☒	A description of all covariates tested
☐	☒	A description of any assumptions or corrections, such as tests of normality and adjustment for multiple comparisons
☐	☒	A full description of the statistical parameters including central tendency (e.g. means) or other basic estimates (e.g. regression coefficient) AND variation (e.g. standard deviation) or associated estimates of uncertainty (e.g. confidence intervals)
☐	☒	For null hypothesis testing, the test statistic (e.g. *F*, *t*, *r*) with confidence intervals, effect sizes, degrees of freedom and *P* value noted<br>*Give P values as exact values whenever suitable.*
☒	☐	For Bayesian analysis, information on the choice of priors and Markov chain Monte Carlo settings
☒	☐	For hierarchical and complex designs, identification of the appropriate level for tests and full reporting of outcomes
☒	☐	Estimates of effect sizes (e.g. Cohen's *d*, Pearson's *r*), indicating how they were calculated

Our web collection on statistics for biologists contains articles on many of the points above.

Software and code

Policy information about availability of computer code

Data collection: No software was used for data collection.

Data analysis: GraphPad Prism software (v.10.1.0) was used for statistical analysis.
GraphPad Prism software (v.10.1.0), Microsoft PowerPoint (v16.83) and Adobe Illustrator (v28.3) were used for graphical representation of the data.

For metabolomics, transition lists, retention time and raw data were loaded into Skyline (v23.1). Data were plotted using python v3.9, and the packages matplotlib v3.5.1, numpy v1.22.2, pandas v1.4.1, seaborn v0.12.0, statannotations v0.4.4.

Flow cytometry analyis was performed using BD FACS Diva (v8.0.1), FlowJo (v10.8.1) or NovoExpress (v1.6.2).

Confocal imaging was acquired using ZEN Black edition (v2.3 SP) and image analysis was done with Image J 1.53t Java 1.8.9_322 (64 bit).

For manuscripts utilizing custom algorithms or software that are central to the research but not yet described in published literature, software must be made available to editors and reviewers. We strongly encourage code deposition in a community repository (e.g. GitHub). See the Nature Portfolio guidelines for submitting code & software for further information.

Data

Policy information about availability of data

All manuscripts must include a data availability statement. This statement should provide the following information, where applicable:

- Accession codes, unique identifiers, or web links for publicly available datasets
- A description of any restrictions on data availability
- For clinical datasets or third party data, please ensure that the statement adheres to our policy

Sequencing data are uploaded to GEO with the accession number GSE232509.
Metabolomics data are uploaded to MassIVE under the accession number MSV000094355.
Source data are provided with this paper.
All data generated in this study are provided in the Supplementary Information/Source Data File.

Research involving human participants, their data, or biological material

Policy information about studies with human participants or human data. See also policy information about sex, gender (identity/presentation), and sexual orientation and race, ethnicity and racism.

Reporting on sex and gender	Table S1 and S2 report amongst other information the sex of the patient material used in the study.<br><br>(Figure 3 and S5). The organoid biobank used in this proposal has been established from a randomly selected patient cohort, which closely represents the clinical incidence of colorectal cancer. Therefore, the anticipated results will be representative for both male and female subjects although the incidence of colorectal cancer worldwide is higher in men with a relative distribution of about 54% vs. 46% in women which is likely linked to differences in other risk factors (e.g. lifestyle, awareness, screening).<br><br>(Figure S6). All patients included in the study provided informed consent for participation. Consent included permission to publish personal health information, including sex, age, diagnosis, and medical center. Patients were not compensated to participate in the study. Three patients were included in the study (Table S2). The sex of study participants was assigned based on the sex-specific individual Danish civil registration number and was considered in the study design or patient enrollment.
Reporting on race, ethnicity, or other socially relevant groupings	We did not report on race, ethnicity or any other socially relevant information on the patients.
Population characteristics	The covariate-relevant population characteristics of the human research participants include : age, sex, tumor localization, cancer grade classification, prior treatment and presence or not of the matching normal organoids in Table S1.<br><br>The covariate-relevant population characteristics of the human research participants include : age, sex, tumor localization, and diagnosis in Table S2.
Recruitment	The patients were selected based on diagnosis.
Ethics oversight	Resection samples from colorectal cancer patients were provided by the University Cancer Center Frankfurt (UCT). All materials and associated data (including age, sex, MMR status, tumor localisation, UICC classification, and prior radio-/chemotherapy) were collected after pseudonymization as part of the interdisciplinary Biobank and Database Frankfurt (iBDF) and the study was approved by the institutional review board of the UCT and the Ethical Committee at the University Hospital Frankfurt (Ethics vote: 4/09; project-number SGI-10-2022). Informed consent was obtained by the participants and included permission to publish personal health information. Patients were not compensated to participate in the study. The organoid biobank used in this proposal has been established from a randomly selected patient cohort, which closely represents the clinical incidence of colorectal cancer. Therefore, the anticipated results will be representative for both male and female subjects although the incidence of colorectal cancer worldwide is higher in men with a relative distribution of about 54% vs. 46% in women which is likely linked to differences in other risk factors (e.g. lifestyle, awareness, screening).<br><br>All procedures with human brain tumor tissue and data were approved by the Central Denmark Region Committee for Health Research Ethics (official name in Danish: De Videnskabsetiske Komitéer for Region Midtjylland); journal number: 1-10-72-82-17) and conducted in accordance with the ethical principles of the World Medical Association Declaration of Helsinki . Surgical specimens were obtained at Aarhus University Hospital (Denmark) from patients undergoing resection of primary or secondary brain tumors. The study specimens were surplus to diagnostic requirements. All patients included in the study provided informed consent for participation. Consent included permission to publish personal health information, including sex, age, diagnosis, and medical center. Patients were not compensated to participate in the study.

Note that full information on the approval of the study protocol must also be provided in the manuscript.

Field-specific reporting

Please select the one below that is the best fit for your research. If you are not sure, read the appropriate sections before making your selection.

- [x] Life sciences
- [ ] Behavioural & social sciences
- [ ] Ecological, evolutionary & environmental sciences

For a reference copy of the document with all sections, see nature.com/documents/nr-reporting-summary-flat.pdf

Life sciences study design

All studies must disclose on these points even when the disclosure is negative.

Sample size

In vitro:
For in vitro work, no sample size calculations were performed. Sample sizes were chosen based on previous experience with in vitro cell experiments. The number of experiments and replicates were based on standard practices. Details for each experiment are included in the figure legends.

In vivo:
For in vivo experiments performed in the Netherlands (Figure 6), group size was calculated using the PS: Power and Sample Size Calculation program (Vanderbilt University, V.3.1.6).
For in vivo experiments performed in Denmark (Figure 2), group sizes were determined to allow for detection of at least 50% reduction in tumor growth from 1000 mm3 to 500mm3 with 80% power at false positive rate of 0.05 (alpha).

Data exclusions

In vitro:
We had to exclude a few samples for technical reasons during flow cytometry (i.e. flow cytometer got stuck).
This is explicitly mentioned in the manuscript. “Samples were excluded when no viable cells were acquired”

In vivo (Figure 6):
Exclusion decision tree:
• Mouse 3316 (CDX-4-OI/PBS group) removed from whole experiment because it was a female when it arrived, instead of male.
• Mouse 3312, 3313 and 3323 (CDX-PBS group) removed from whole experiment due to reaching humane endpoints because of fighting wounds.
• Lymphoid panel: Spleen 3308 (CDX-4-OI/VSV group), Tumor 3327 (CDX/VSV group), TDLN 3327 (CDX/VSV) group removed from lymphoid panel due to loss of sample because of clogging Fortessa
• RT-qPCR analysis: mouse 3308 (CDX-4-OI/VSV group), 3318 (CDX-4-OI/VSV) excluded because of insufficient tumor material (smallest tumors)

Replication

All experiments were replicated at least once in an independent setup - All attempts to replicate experiments were successful.
The number of replicates is indicated within figure legends.

Randomization

In vitro:
Experiments performed in vitro were done using unbiased techniques. Therefore, randomization was not used.

In vivo experiments done in Denmark: After establishment of tumors, mice were distributed into treatment groups to approach equal median tumor sizes before beginning of treatment

Blinding

In vitro:
All experiments were performed in vitro using unbiased measuring techniques that are independent of the scientist performing the experiment. Therefore, blinding was not used

In vivo:
Blinding was not used for all in vivo experiments performed in the study

Reporting for specific materials, systems and methods

We require information from authors about some types of materials, experimental systems and methods used in many studies. Here, indicate whether each material, system or method listed is relevant to your study. If you are not sure if a list item applies to your research, read the appropriate section before selecting a response.

Materials & experimental systems

n/a	Involved in the study	
	☒	Antibodies
	☒	Eukaryotic cell lines
☒		Palaeontology and archaeology
	☒	Animals and other organisms
	☒	Clinical data
☒		Dual use research of concern
☒		Plants

Methods

n/a	Involved in the study	
☒		ChIP-seq
	☒	Flow cytometry
☒		MRI-based neuroimaging

Antibodies

Antibodies used

Flow cytometry (human cells):
Anti-Human-LDLR Antibody, Clone C7, BV421-conjugated (1:80) (BD744847)
IgG2 kappa Isotope Control BV421-conjugated (1:80) (BD569376)

Flow cytometry (murine cells):
AF700 CD8 53-6.7 56-0081-82 Thermofisher 1:100
AF700 CD45.2 104 109822 Biolegend 1:400
APC NKG2a 16A11 142807 Biolegend 1:100
APC XCR1 ZET 148206 Biolegend 1:200
APC-Cy7 PD1 29F.1A12 135224 Biolegend 1:50
APC-Cy7 CD11c N418 117324 Biolegend 1:100
BV421 CD62L MEL-14 104436 Biolegend 1:100
BV421 MHC-II M5/114 562564 BD 1:400
BV510 Zombie Aqua 423101 Biolegend 1:800
BV605 CD69 H1.2F3 104530 Biolegend 1:100
BV605 Ly6C HK1.4 128036 Biolegend 1:500
BV650 NK1.1 PK136 564143 BD 1:100
BV650 Siglec-H 440c 747672 BD 1:300
BV711 CD4 RM4-5 100549 Biolegend 1:200
BV711 CD103 2E7 121435 Biolegend 1:200
BV786 CD44 IM7 103059 Biolegend 1:100
BV786 Ly6G 1A8 127645 Biolegend 1:400
FITC CD45.2 104 109806 Biolegend 1:200
FITC CD11b ICRF44 562793 BD 1:500
PE Foxp3 FJK-16s 12-5773-80 Thermofisher 1:200
PE CD86 GL-1 105007 Biolegend 1:400
PECF594 CD3 145-2C11 562286 BD 1:200
PE-Cy7 KLRG-1 2F1 25-5893-82 Invitrogen 1:200
PE-Cy7 F4/80 BM8 123112 Biolegend 1:100
CD16/CD32 2.4G2 553142 BD 1:250 (Mouse BD FC Block)

Immunoblotting:
VSV antisera (a gift from Dr. Jean-Simon Diallo, 1:10.000)
anti-AKR1B10 (SC-365689, Santa Cruz,, 1:1000)
anti-Cleaved PARP (5625, Cell Signaling, 1:1000)
anti-GAPDH (sc-47724, Santa Cruz, 1:1000)
anti-HO1 (86806, Cell Signaling, 1:1000)
anti-IFIT1 (14769, Cell Signaling, 1:1000)
anti-IRF3 (11904, Cell Signaling, 1:1000)
anti-IKKβ (2684, Cell Signaling, 1:1000)
anti-ISG15 (2758, Cell Signaling, 1:1000)
anti-Jak1 (29261, Cell Signaling, 1:1000)
anti-KEAP1 (8047, Cell Signaling, 1:1000)
anti-MAVS (3993, Cell Signaling, 1:1000)
anti-NRF2 (ab62352, Abcam, 1:1000)
anti-NF-kB p65 (8242, Cell Signaling, 1:1000)
anti-P-IRF3 Ser396 (29047, Cell Signaling, 1:500)
anti-P-STAT1 Tyr701 (7649, Cell Signaling, 1:1000)
anti-P-IKKa/β (2697, Cell Signaling, 1:1000)
anti-P-IIkba(2859, Cell Signaling 1:1000)
anti-P-NF-kB p65 Ser536 (3033, Cell Signaling, 1:1000)
anti-DYKDDDDK Tag (D6W5B) (FLAG) (14793, Cell Signaling, 1:1000)

anti-TET2 (18950, Cell Signaling, 1:1000)
anti-ATF3 (33593, Cell Signaling, 1:1000)
anti-IkB-zeta (9244, Cell Signaling, 1:1000)
anti-IKKγ (2685, Cell Signaling, 1:1000)
anti-IKKε (2905, Cell Signaling, 1:1000)
anti-Vinculin (Sigma-Aldrich 1:10,000)
peroxidase conjugated F(ab)2 donkey anti-mouse IgG (H+L) (1:10000) (Jackson ImmunoResearch)
peroxidase conjugated F(ab)2 donkey anti-rabbit Ig (H+L) (1:10000) (Jackson ImmunoResearch)

anti-RIG-I (1:5000, EMD-Millipore, Cat# D14GG,)
anti-ISG56 (1:5000, PA3-848, Thermo Fischer Scientific)
anti-Actin (1:10000, EMD Millipore Cat# MAB1501)
anti-GFP (1:3000, Santa Cruz Biotechnology, Cat# SC-9996)
monoclonal FLAG antibody M2 (1:5000, Sigma Cat#F1804)
SeraCare Cat# 5450 0010 anti-Rabbit HRP, 1:5000
SeraCare Cat #5450 0011 anti-mouse HRP, 1:5000

Co-Immunoprecipitation
anti-GFP (1:3000, Santa Cruz Biotechnology, Cat# SC-9996)
anti-Flag antibodies (1:5000, Sigma Cat#F1804)
SeraCare Cat# 5450 0010 anti-Rabbit HRP, 1:5000
SeraCare Cat #5450 0011 anti-mouse HRP, 1:5000

Confocal microscopy:
VSV glycoprotein antibody (SAB4200695, Sigma, 1:200)
cleaved caspase3 antibody ((Asp175) 5A1E, Cell Signaling, 1:400)
IFIT1 antibody (14769, Cell Signaling, 1:800)
NRF2 antibody (12721, Cell Signaling, 1:200)
cleaved P65 antibody (8242, Cell Signaling, 1:200)
goat anti rabbit Alexa Fluor 488 nm fluorophore-conjugated secondary antibody (A11008, Invitrogen, 1:400)
goat antimouse Alexa Fluor 555 nm fluorophore-conjugated secondary antibody (A21424, Invitrogen, 1:400)

Validation

All the commercially available antibodies used throughout the study have been verified by their respective manufacturers.
Wherever possible, antibodies were validated in our hands using molecular size and/or CRISPR KO gene editing

Flow cytometry (humand cells):
Anti-Human-LDLR Antibody, Clone C7, BV421-conjugated (1:80) (BD744847) : validated by CRISPR KO (Fig. S8b) and the use of the isotype control below
IgG2 kappa Isotope Control BV421-conjugated (1:80) (BD569376)

Flow cytometry (murine cells):
Antibodies were validated and titrated on C57Bl/6 mouse splenocytes or blood leukocytes before using in panel
AF700 CD8 53-6.7 56-0081-82 Thermofisher 1:100
AF700 CD45.2 104 109822 Biolegend 1:400
APC NKG2a 16A11 142807 Biolegend 1:100
APC XCR1 ZET 148206 Biolegend 1:200
APC-Cy7 PD1 29F.1A12 135224 Biolegend 1:50
APC-Cy7 CD11c N418 117324 Biolegend 1:100
BV421 CD62L MEL-14 104436 Biolegend 1:100
BV421 MHC-II M5/114 562564 BD 1:400
BV510 Zombie Aqua 423101 Biolegend 1:800
BV605 CD69 H1.2F3 104530 Biolegend 1:100
BV605 Ly6C HK1.4 128036 Biolegend 1:500
BV650 NK1.1 PK136 564143 BD 1:100
BV650 Siglec-H 440c 747672 BD 1:300
BV711 CD4 RM4-5 100549 Biolegend 1:200
BV711 CD103 2E7 121435 Biolegend 1:200
BV786 CD44 IM7 103059 Biolegend 1:100
BV786 Ly6G 1A8 127645 Biolegend 1:400
FITC CD45.2 104 109806 Biolegend 1:200
FITC CD11b ICRF44 562793 BD 1:500
PE Foxp3 FJK-16s 12-5773-80 Thermofisher 1:200

PE CD86 GL-1 105007 Biolegend 1:400
PECF594 CD3 145-2C11 562286 BD 1:200
PE-Cy7 KLRG-1 2F1 25-5893-82 Invitrogen 1:200
PE-Cy7 F4/80 BM8 123112 Biolegend 1:100
CD16/CD32 2.4G2 553142 BD 1:250

Immunoblotting:
VSV antisera (a gift from Dr. Jean-Simon Diallo, 1:10.000) : validated by molecular size and by infection with/without VSV (Fig. 4d, 4h, 4i, 5e, 5g, 5h, 7f, 8j)
anti-AKR1B10 (SC-365689, Santa Cruz,, 1:1000) : validated by molecular size
anti-Cleaved PARP (5625, Cell Signaling, 1:1000) : validated by molecualr size
anti-GAPDH (sc-47724, Santa Cruz, 1:1000) : validated by molecular size
anti-HO1 (86806, Cell Signaling, 1:1000) : validated by molecular size
anti-IFIT1 (14769, Cell Signaling, 1:1000) : validated by molecular size
anti-IRF3 (11904, Cell Signaling, 1:1000): validated by molecular size and CRISPR KO (Fig. S12a)
anti-IKKβ (2684, Cell Signaling, 1:1000) : validated by molecular size and CRISPR KO (Fig. 8j)
anti-ISG15 (2758, Cell Signaling, 1:1000) : validated by molecular size
anti-Jak1 (29261, Cell Signaling, 1:1000) : validated by molecular size and CRISPR KO (Fig. S13f)
anti-KEAP1 (8047, Cell Signaling, 1:1000) : validated by molecular size and CRISPR KO (Fig. 4i)
anti-MAVS (3993, Cell Signaling, 1:1000): validated by molecular size and CRISPR KO (Fig. 7f)
anti-NRF2 (ab62352, Abcam, 1:1000) : validated by molecular size and CRISPR KO (Fig. 4d, 4h)
anti-NF-kB p65 (8242, Cell Signaling, 1:1000) : validated by molecular size and CRISPR KO (Fig. S15c)
anti-P-IRF3 Ser396 (29047, Cell Signaling, 1:500) validated by molecular size
anti-P-STAT1 Tyr701 (7649, Cell Signaling, 1:1000) : validated by molecular size
anti-P-IKKa/β (2697, Cell Signaling, 1:1000) : validated by molecular size
anti-P-IIkba(2859, Cell Signaling 1:1000) : validated by molecular size
anti-P-NF-kB p65 Ser536 (3033, Cell Signaling, 1:1000) : validated by molecular size
anti-DYKDDDDK Tag (D6W5B) (FLAG) (14793, Cell Signaling, 1:1000)
anti-TET2 (18950, Cell Signaling, 1:1000) : validated by molecular size and CRISPR KO (Fig. S15e)
anti-ATF3 (33593, Cell Signaling, 1:1000) : validated by molecular size and CRISPR KO (Fig. S15a)
anti-IkB-zeta (9244, Cell Signaling, 1:1000) : validated by molecular size
anti-IKKγ (2685, Cell Signaling, 1:1000) : validated by molecular size
anti-IKKε (2905, Cell Signaling, 1:1000): validated by molecular size
anti-Vinculin (Sigma-Aldrich 1:10,000) : validated by molecular size
peroxidase conjugated F(ab)2 donkey anti-mouse IgG (H+L) (1:10000) (Jackson ImmunoResearch)
peroxidase conjugated F(ab)2 donkey anti-rabbit Ig (H+L) (1:10000) (Jackson ImmunoResearch)

anti-RIG-I (1:5000, EMD-Millipore, Cat# D14GG): validated by molecular size
anti-ISG56 (1:5000, PA3-848, Thermo Fischer Scientific) : validated by molecular size
anti-Actin (1:10000, EMD Millipore Cat# MAB1501) : validated by molecular size
anti-GFP (1:3000, Santa Cruz Biotechnology, Cat# SC-9996) : validated by molecular size
monoclonal FLAG antibody M2 (1:5000, Sigma Cat#F1804) : validated by molecular size
SeraCare Cat# 5450 0010 anti-Rabbit HRP, 1:5000
SeraCare Cat #5450 0011 anti-mouse HRP, 1:5000

Co-Immunoprecipitation
anti-GFP (1:3000, Santa Cruz Biotechnology, Cat# SC-9996): validated by molecular size
anti-Flag antibodies (1:5000, Sigma Cat#F1804): validated by molecular size
SeraCare Cat# 5450 0010 anti-Rabbit HRP, 1:5000
SeraCare Cat #5450 0011 anti-mouse HRP, 1:5000

Confocal microscopy:
VSV glycoprotein antibody (SAB4200695, Sigma, 1:200) : validated by infection with/without VSV
cleaved caspase3 antibody ((Asp175) 5A1E, Cell Signaling, 1:400) : validated by absence of signal in presence of secondary antibody only
IFIT1 antibody (14769, Cell Signaling, 1:800) : validated by absence of signal in presence of secondary antibody only
NRF2 antibody (12721, Cell Signaling, 1:200) : validated by CRISPR KO (Fig. 4a)
cleaved P65 antibody (8242, Cell Signaling, 1:200) : validated by absence of signal in presence of secondary antibody only
goat anti rabbit Alexa Fluor 488 nm fluorophore-conjugated secondary antibody (A11008, Invitrogen, 1:400)
goat antimouse Alexa Fluor 555 nm fluorophore-conjugated secondary antibody (A21424, Invitrogen, 1:400)

Eukaryotic cell lines

Policy information about cell lines and Sex and Gender in Research

Cell line source(s)	CT26WT, B16F10 and NCI-H358 cells were a kind gift from Martin R. Jakobsen (Aarhus University).<br>HT-29 cells were provided by Lasse S. Christensen (Aarhus University).<br>Vero cells were from Søren R. Paludan (Aarhus University).<br>DK-MG and H4 cell were generously given by Anna-Liisa Levonen (Virtanen Institute, Kuopio, Finland).

	4T1 and HEK 293T cells were a gift from Tommy Alain (University of Ottawa).<br>786-O cells were purchased from ATCC.<br>Experiments with the human BxPC3 cells were performed in the Hiscott laboratory (Pasteur Institute, Rome). Cells were obtained from ATCC.<br>Experiments with the PANC1 cell line were performed in the Alain laboratory (Ottawa University/CHEO).
Authentication	CT26WT cells were authenticated at the beginning of the project. Genetic material from the CT26WT cells was examined for mouse short tandem repeat (STR) profile and interspecies contamination (Cellcheck 19- mouse, IDEXX BioAnalytics Germany). No interspecies contamination was detected. The cell-line was confirmed to have more than 80% identity match with reference cell-line ATCC# CRL 2638.<br><br>786-O cells were bought from ATCC at the beginning of the project. Cell lines from ATCC were authenticated by the vendor using short tandem repeat (STR) analysis, and were not validated further in our laboratory<br><br>All other lines were not authenticated
Mycoplasma contamination	All cell lines were tested routinely for Mycoplasma infection. All tests were negative
Commonly misidentified lines<br>(See ICLAC register)	HT-29; Vero; DK-MG; 786-0<br>HT-29, DK-MG and 786-O cell lines were selected in our study based on their basal infectivity profile to VSVD51<br>Vero cells were used as a standard model to determine viral titers by plaque assay.

Animals and other research organisms

Policy information about studies involving animals; ARRIVE guidelines recommended for reporting animal research, and Sex and Gender in Research

Laboratory animals	In vivo Canada: Five- to six-week-old female BALB/c mice were purchased from Charles River (Kingston, New York, USA).<br>In vivo Denmark: Five- to six-week-old female BALB/c mice were purchased from Janvier Labs (France)<br>In vivo the Netherlands: Six- to eight-week-old male BALB/cByJ mice were purchased from Charles River (France)
Wild animals	No wild animals were used in the study
Reporting on sex	In vivo Denmark and Canada (Figure 2):<br>In our survival and tumor growth studies, we chose to work exclusively with female subjects due to practical considerations surrounding animal handling and housing logistics. Females allowed for more efficient use of cage space, as we could comfortably house up to five individuals together, unlike males, who typically require individual cages due to territorial behaviors. We believed that minimizing stressors associated with isolation would lead to more reliable results and ensure the welfare of our experimental subjects.<br><br>In vivo the Netherlands (Figure 6):<br>In this experiment we chose to work exclusively with male subjects for practical/ethical reasons. Due to the stronger skin of male subjects, we expected fewer occasions of tumors breaking to the skin or ulcerations that led to exclusion of mice from experiment due to reaching humane endpoints, and hence reduced power.
Field-collected samples	No field collected samples were used in the study
Ethics oversight	1-Experiments were performed in accordance with the University of Ottawa Animal Care and Veterinary Service guidelines for animal care under the protocol CHEOe-3084-R2 A1.<br><br>2- Experiments were performed at Aarhus University, Department of Biomedicine in accordance with the protocol 2023-15-0201-01489 approved by the Danish Experimental Animal Expectorate.<br><br>3-Experiments were performed in accordance with the institutional Animal Welfare Body of the LUMC and carried out under project license AVD1160020187004, issued by the competent authority on animal experiments in The Netherlands (named CCD). Experiment was performed following the Dutch Act on Animal Experimentation and EU Directive 2010/63/EU ("On the protection of animals used for scientific purposes") at the animal facility.

Note that full information on the approval of the study protocol must also be provided in the manuscript.

Clinical data

Policy information about clinical studies
All manuscripts should comply with the ICMJEguidelines for publication of clinical research and a completedCONSORT checklist must be included with all submissions.

Clinical trial registration	We did not perform a clinical trial in this study.
Study protocol	1-Resection samples from colorectal cancer patients were provided by the University Cancer Center Frankfurt (UCT). All materials

were collected as part of the interdisciplinary Biobank and Database Frankfurt (iBDF) after prior written informed consent and the study was approved by the institutional review board of the UCT and the Ethical Committee at the University Hospital Frankfurt (Ethics vote: 4/09; project-numbers: SGI-06-2015 and SGI-10-2022).

2-All procedures with human brain tumor tissue and data were approved by the Central Denmark Region Committee for Health Research Ethics (official name in Danish: De Videnskabsetiske Komitéer for Region Midtjylland); journal number: 1-10-72-82-17) and conducted in accordance with the ethical principles of the World Medical Association Declaration of Helsinki 66. Surgical specimens were obtained at Aarhus University Hospital (Denmark) from patients undergoing resection of primary or secondary brain tumors. The study specimens were surplus to diagnostic requirements. All patients included in the study provided informed consent for participation.

Data collection

1-Resection samples from colorectal cancer patients were provided by the University Cancer Center Frankfurt (UCT). All materials were collected as part of the interdisciplinary Biobank and Database Frankfurt (iBDF) after prior written informed consent and the study was approved by the institutional review board of the UCT and the Ethical Committee at the University Hospital Frankfurt (Ethics vote: 4/09; project-numbers: SGI-06-2015 and SGI-10-2022).

2-All procedures with human brain tumor tissue and data were approved by the Central Denmark Region Committee for Health Research Ethics (official name in Danish: De Videnskabsetiske Komitéer for Region Midtjylland); journal number: 1-10-72-82-17) and conducted in accordance with the ethical principles of the World Medical Association Declaration of Helsinki 66. Surgical specimens were obtained at Aarhus University Hospital (Denmark) from patients undergoing resection of primary or secondary brain tumors. The study specimens were surplus to diagnostic requirements. All patients included in the study provided informed consent for participation.

Outcomes

N/A

Plants

Seed stocks

N/A

Novel plant genotypes

N/A

Authentication

N/A

Flow Cytometry

Plots

Confirm that:

- [x] The axis labels state the marker and fluorochrome used (e.g. CD4-FITC).
- [x] The axis scales are clearly visible. Include numbers along axes only for bottom left plot of group (a 'group' is an analysis of identical markers).
- [x] All plots are contour plots with outliers or pseudocolor plots.
- [x] A numerical value for number of cells or percentage (with statistics) is provided.

Methodology

Sample preparation

1-The percentage of virus RFP-positive cells was quantified using flow cytometry. Briefly, 786-O or CT26WT cells were seeded in a 12-well plate (2.5 × 105 /well) and treated the next day with 4-OI (75μM) for 24 hours following an infection with VSVΔ51 at an MOI of 0.01. After one hour of infection, the supernatant was removed to eliminate unbound virus and replenished with complete growth medium containing 4-OI with the same concentration. At 17-18 hours post infection, cells were harvested and the LIVE/DEAD Fixable Green Dead Cell Stain kit with 488 nm excitation (Invitrogen) was used

2-The percentage of VSVΔ51-RFP-positive cells within colon organoids was quantified using flow cytometry at 48 hours of post-infection. Organoids were mechanically disrupted by pipetting, washed with PBS and centrifuged for 5 minutes at 1200 rpm 4 °C. Then, the supernatant was removed and 1 mL of StemPro Accutase (Gibco) was added and incubation for 5 min at 37 °C with gentle vortexing was performed. Then, fresh DMEM media was quickly added and enzymatically digested organoids were centrifuged again for 5 minutes at 1500 rpm 4 °C. After one more wash in PBS, the pellet was resuspended in 4% PFA in PBS and filtered once or twice through 40 μm cell strainer using round bottom tubes with cell strainer cap (Stem cell Technologies).

3-At the dedicated timepoint, spleen, tumor draining lymph node (T-DLN) and tumor was collected for flowcytometry analysis and representative part of tumor was snap frozen for RNA analysis. Organs were dissociated into single-cell suspension as described in 74. Cells were incubated with Zombie Aqua Fixable Viability dye (Biolegend) in PBS for 20 minutes

	followed by incubation with 2.4G2 FcR blocking antibodies (clone 2.4G2, BD Biosciences in FACS buffer (PBS, 0.5% BSA and 1% sodium azide) for 20min on ice followed by incubation with a mix of conjugated antibodies (Table S4) in FACS buffer for 30 minutes on ice. If necessary, cells were fixed and stained for nuclear proteins using the Foxp3 / Transcription Factor Staining Buffer Set (eBiosciences) according to manufacturers' instructions. After completing staining protocol, samples were fixed in 1% paraformaldehyde and measured using a BD LSRFortessa X20 cell analyzer (BD Biosciences) at the Flow cytometry Core Facility (FCF) of Leiden University Medical Center (LUMC) in Leiden, Netherlands. Samples were excluded from analysis if no viable cells were acquired. Four markers had to be excluded from analysis due to low signal, which may be due to aberrant expression in Balb/C hosts compared to C57Bl/6. Flow cytometry data was analyzed using FlowJo™ Software Version 10 (Becton, Dickinson and Company). Opt-SNE plots were generated using standard settings in OMIQ data analysis software (Omiq, Inc. www.omiq.ai). The gating strategy is described in Fig. S11.
Instrument	1-Flow cytometry analysis was performed using a NovoCyte Quanteon instrument, and data were analyzed using NovoExpress Software<br><br>2-Flow cytometry counts were performed using BD LSRFortessa cell analyzer (BD Biosciences), and was analyzed with BD FACSDiva Software at the Flow cytometry core of the Biomedicine Department at Aarhus University.<br><br>3-After completing staining protocol, samples were measured using a BD LSRFortessa X20 cell analyzer (BD Biosciences) at the Flow cytometry Core Facility (FCF) of Leiden University Medical Center (LUMC) in Leiden, Netherlands.
Software	1-Flow cytometry analysis was performed using a NovoCyte Quanteon instrument, and data were analyzed using NovoExpress Software<br><br>2-Flow cytometry counts were performed using BD LSRFortessa cell analyzer (BD Biosciences), and was analyzed with BD FACSDiva Software.<br><br>3-Flow cytometry data using a BD LSRFortessa X20 cell analyzer (BD Biosciences) were analyzed using FlowJo™ 122 Software Version 10 and OMIQ (https://www.omiq.ai/) is used to create opt-TSNE plots.
Cell population abundance	FACS sort is not performed. For flowcytometry data the relevant gating strategy is provided, as can be seen in Fig. S11. Low abundant cell population were not analyzed. Markers with low abundant signal were not included in analysis.
Gating strategy	Gating strategy is provided in different supplementary figures

☒ Tick this box to confirm that a figure exemplifying the gating strategy is provided in the Supplementary Information.
